# Supplementary material for: Selective conversion of CO2 to isobutane-enriched C4 alkanes over InZrOx-Beta composite catalyst
Source: Nat Commun. 2023 May 6;14:2627. doi: 10.1038/s41467-023-38336-5 (PMC10164185; doi:10.1038/s41467-023-38336-5)
Supplement: Supplementary file 1 — Supplementary Information [file 41467_2023_38336_MOESM1_ESM.pdf]

Supplementary Information

Selective Conversion of CO_2 to Isobutane-enriched C_4 alkanes over $InZrO_x$-Beta Composite Catalyst

Han Wang[1,2,3], Sheng Fan[1,2,3], Shujia Guo[1,2], Sen Wang[1,*], Zhangfeng Qin[1,*], Mei Dong[1], Huaqing Zhu[1], Weibin Fan[1] & Jianguo Wang[1,2,*]

[1]*State Key Laboratory of Coal Conversion, Institute of Coal Chemistry, Chinese Academy of Sciences, P.O. Box 165, Taiyuan, Shanxi 030001, P. R. China*

[2]*University of Chinese Academy of Sciences, Beijing 100049, P. R. China*

[3]*These authors contributed equally to this work: Han Wang, Sheng Fan*

[*] *Corresponding authors. Tel.: +86-351-4046092; Fax: +86-351-4041153. E-mail address: wangsen@sxicc.ac.cn (S. Wang); qzhf@sxicc.ac.cn (Z. Qin); iccjgw@sxicc.ac.cn (J. Wang)*

Table of Contents

Supplementary Methods

Supplementary Figures

Supplementary Tables

Supplementary References

Supplementary Methods

1. Catalyst Preparation.

$InZrO_x$(HT). The $InZrO_x$(HT) oxide was prepared by hydrothermal method. 2.2562 g of $In(NO_3)_3·H_2O$ and 0.8050 g of $Zr(NO_3)_4·5H_2O$ with an In/Zr molar ratio of 4 were first dissolved in 15 mL deionized water to obtain the solution A. Subsequently, 25.2 g of NaOH was dissolved into 70 mL deionized water to obtain the solution B. These two solutions were then mixed and stirred continuously for 0.5 h. After that, they were transferred into a stainless steel autoclave lined with Teflon and heated at 100 °C for 24 h. The resultant precipitate was centrifuged and washed with deionized water for three times, and then dried at 105 °C and calcined at 500 °C for 3 h.

$InZrO_x$(CP). The $InZrO_x$(CP) oxide was prepared by co-precipitation method. First, solution A was obtained by dissolving 2.2562 g of $In(NO_3)_3·H_2O$ and 0.8050 g of $Zr(NO_3)_4·5H_2O$ with an In/Zr molar ratio of 4 into 20 mL deionized water; solution B was obtained by dissolving 2.42 g of $(NH_4)_2CO_3$ into another 20 mL deionized water. Next, solution A and solution B were simultaneously dropped into a beaker containing 100 mL deionized water and stirred magnetically at 70 °C for 3 h. The resultant precipitate was then washed and centrifuged with deionized water, and then dried at 105 °C and calcined at 500 °C for 3 h.

$InZrO_x$(SG). The $InZrO_x$(SG) oxide was prepared by the sol-gel method. 2.2562 g of $In(NO_3)_3·H_2O$ and 0.8050 g of $Zr(NO_3)_4·5H_2O$ were first dissolved in 150 mL deionized water under magnetic stirring. 5.5735 g of glucose was then added into above solution. After stirring at 80 °C for 6 h, the obtained sol was dried at 100 °C for 12 h and then calcined at 500 °C for 3 h.

In_2O_3(CP). The In_2O_3(CP) oxide was also obtained by the co-precipitation method, as that of $InZrO_x$(CP), where 2.2562 g of $In(NO_3)_3·H_2O$ was used to prepare solution A and 1.77 g of $(NH_4)_2CO_3$ was used to prepare solution B.

Surface silica-modified $InZrO_x$(SCP) and In_2O_3(SCP-4). 25 mL of *n*-hexane containing 0.1389 g of TEOS were slowly added into 1 g of the $InZrO_x$(CP) or In_2O_3(CP) sample. The resultant mixture was stirred at 80 °C for 1 h. After centrifugation, the attained solid material was dried at 100 °C for 10 h and calcined at 500 °C for 3 h. The obtained

sample having a silica loading of 4 wt.% was denoted as $InZrO_x$(SCP-4) or In_2O_3(SCP-4). In contrast, $InZrO_x$(SCP-8) with a silica loading of 8 wt.% was prepared by the similar procedures, except that the TEOS quantity was increased to 0.2778 g.

H-Beta zeolite. H-Beta zeolites with a Si/Al ratio of 20, 30, 40, 60 and 100 were synthesized by the hydrothermal method, as describe previously.[1] Briefly, TEOS, aluminum powder, TEAOH, and deionized water were first mixed and the mixture was then stirred at 80 °C for 2 h to remove excess water and ethanol. After that, HF was added slowly into above solution to form a gel with the molar composition of $1SiO_2$: nAl_2O_3: (0.54+2n)TEAOH: (7+2n)H_2O: (0.54+2n)HF, with 1/2n being the designed Si/Al molar ratio. The synthesis gel was then transferred into a Teflon-lined stainless steel autoclave and crystallized at 140 °C for 72 h at a rotation rate of 15 rpm. After washing with deionized water and centrifuging, the solid samples were dried at 100 °C for 12 h and then calcined at 560 °C for 10 h. The H-form Beta zeolite (H-Beta) was prepared through ion-exchange of the as-synthesized Beta sample with NH_4NO_3 solution (1 M) twice at 80 °C for 5 h., which was then dried at 100 °C for 12 h and calcined at 560 °C for 10 h.

2. Catalyst Characterization

The X-ray diffraction (XRD) patterns were collected on a Bruker D8 Advance X-ray diffractometer (CuKα radiation, λ = 1.5418 Å, 40 kV and 40 mA), in the 2θ range of 5°–80° and with a scanning rate of 5° min^{-1}. In addition, the XRD patterns of reduced $InZrO_x$ oxides were collected in situ after reducing the oxide samples with H_2 (30 mL min^{-1}) at 400 °C for 2 h. The Rietveld refinement was made with the HighScore plus software.

The textural properties of various samples were measured by N_2 sorption on a Micromeritics TriStar II 3020 instrument at −196 °C. Prior to each measurement, the catalyst sample was degassed at 300 °C under vacuum for 8 h. The total surface area was obtained from the adsorption branch isotherm in the relative pressure range of 0.05–0.25 by the BET method, whilst the pore volume was calculated from the desorption isotherm by the t-plot method and the pore size distribution was analyzed by the BJH method.

The X-ray photoelectron spectra (XPS) of O (1s), Si (2p), In (3d) and Zr (3d) were measured at 5×10^{-7} Pa on an AXIS ULTRA DLD instrument with an Al Kα monochromator X-ray source ($h\nu$ = 1486.6 eV); the binding energies were calibrated by adventitious carbon deposit C (1s) with the binding energy of 284.6 eV. In the in situ XPS

measurement, the sample was first placed in an auxiliary chamber and pretreated at 400 °C for 2 h in a H_2 flow (30 mL min^{-1}); after that, it was then quickly transferred to the measurement chamber purging with Ar and then evacuated to high vacuum.

Field emission-scanning electron microscopy (FE-SEM) images were taken on a JEOL JSM-7001F instrument. High-resolution transmission electron microscopy (HRTEM) and TEM images were measured on a field emission-transmission electron microscope (JEM-2100F, JEOL). The mean size of nanoparticles (NP) was estimated by randomly counting more than 100 particles shown in the TEM images. Aberration-corrected high-angle annular dark-field scanning TEM (Aberration-corrected HAADF-STEM) and the EDX elemental mapping images were acquired on Thermo Scientific, Themis Z equipped with an EDX detector.

H_2 temperature-programmed reduction (H_2-TPR) was carried out on a Micromeritics AutoChem II 2920. 0.1 g of catalyst sample was loaded into a U-shaped quartz reactor and pretreated at 300 °C for 1 h in a He flow (30 mL min^{-1}) to remove any surface impurities. The catalyst sample was cooled to room temperature and exposed to a 10% H_2/Ar flow (30 mL min^{-1}). After that, the catalyst sample was then heated to 800 °C at a ramp of 10 °C min^{-1}, during which the quantity of hydrogen desorbed upon heating in the effluent was recorded using a thermal conductivity detector (TCD), to get the H_2-TPR profiles.

CO_2 temperature-programmed desorption (CO_2-TPD) were also carried out on the Micromeritics AutoChem II 2920 apparatus. 0.05 g of catalyst sample was first loaded into a U-shaped quartz reactor and pretreated at 300 °C for 60 min in the He flow. It was then cooled to 50 °C to allow a saturated adsorption of CO_2. After that, the sample was swept with the He flow and the CO_2-TPD profiles were then recorded at 50–600 °C with a heating rate of 10 °C min^{-1}, by measuring the quantity of desorbed CO_2 with the TCD.

NH_3 temperature-programmed desorption (NH_3-TPD) was performed on the Micromeritics AutoChem II 2920. First, 0.1 g of catalyst sample was treated at 550 °C for 30 min in the He flow and then cooled to 120 °C. After saturated with NH_3, He flow was used to purge the unabsorbed NH_3. Finally, the NH_3-TPD profiles were acquired by heating the sample from 120 to 550 °C at a heating rate of 10 °C min^{-1}.

The elemental composition of H-Beta zeolite (including the Si/Al ratio) was measured by an inductively coupled plasma-atomic emission spectrometer (ICP-AES, iCAP6300).

The in situ diffuse reflectance infrared Fourier transform (DRIFT) spectra were recorded on a Bruker Vertex 80 infrared spectrometer equipped with a liquid nitrogen cooled MCT detector and an in situ high-temperature chamber. The catalyst sample was placed in the in situ chamber and pretreated at 200 °C for 0.5 h with an Ar flow (30 mL min^{-1}). After that, the background spectrum was collected. Subsequently, the catalyst sample was exposed to a H_2 and CO_2 mixture flow (H_2/CO_2 = 3, 40 mL min^{-1}) at 200 °C; the IR spectra in the range of 4000–1000 cm^{-1} were then recorded every 1 min up to 10 min at a resolution of 4 cm^{-1} by accumulating 128 scans.

TG and DTA analyses were conducted on a Rigaku Thermo Plus Evo TG 8120 thermogravimetric analyzer by ramping the temperature from room temperature to 800 °C at 10 °C min^{-1}. The weight loss in region I (<350 °C) was attributed to the desorption of water and decomposition of light hydrocarbons, whereas the weight loss in region II (350–800 °C) was due to the combustion of coke species. The coke deposition rate (in h^{-1}) was calculated as the relative weight loss in region II divided by the reaction time.

The residual species trapped in the zeolite catalyst were identified by GC-MS. 30 mg spent zeolite powder was dissolved with 1 mL of HF (20 wt%) solution, which was then extracted with 1 mL of CH_2Cl_2. The resultant solution was then analyzed on a Shimadzu QP-2010 GC-MS equipped with a 5 MS or DB-1 capillary column.

$^{12}C/^{13}C$ methanol switching experiment was conducted at 315 °C and atmospheric pressure in a U-type quartz tube having an inner diameter of 4 mm, with Ar as the carrier gas. 100 mg of H-Beta(40) was placed in the constant temperature zone of the reactor between two quartz sand layers. The zeolite catalyst was first pretreated at 500 °C for 2 h in an Ar flow (50 mL min^{-1}) and then cooled to 315 °C. After that, ^{12}C-methanol was fed into the reactor via a methanol vapor saturator under ice-water-bath for 25 min, followed by switching to ^{13}C-methanol stream for 1 min.

3. DFT Calculation

The theoretical calculations were carried out using the Gaussian 09 package by density functional theory including dispersion correction (DFT-D). The H-Beta zeolite was modeled by an extended 79 T cluster, which contains two vertical 12-membered ring channels. The framework Al atom was located at the T9 position and the charge-balancing proton was sited in O12 position.[2,3] The ONIOM (ωB97X-D/6-31G(d,p):PM6) method

was used for geometry optimizations and frequency calculations. To obtain accurate interaction energies, single-point calculations were carried out by ωB97X-D/6-311G(2df,2p) method. All atoms in the high layer of cluster and the reactant molecules were allowed to fully relax, while the other atoms in low layer were constrained to prevent unphysical deformations. The free energy barriers ($\Delta G^{\neq}_{\text{int}}$), rate constants ($k$), enthalpy barriers ($\Delta H^{\neq}_{\text{int}}$), and entropy losses ($-T\Delta S_{int}{}^{\neq}$) were obtained. The rate constant at 588 K was calculated by classical transition-state theory:

$$k = \frac{k_B T}{h}\exp\left(-\Delta G^{\neq}_{\text{int}}/RT\right) = \frac{k_B T}{h}\exp\left(\Delta S^{\neq}_{\text{int}}/R\right)\exp\left(-\Delta H^{\neq}_{\text{int}}/RT\right) \quad (1)$$

where k_B is Boltzmann's constant, h is Planck's constant, and $\Delta G^{\neq}_{\text{int}}, \Delta H^{\neq}_{\text{int}}$, and $\Delta S^{\neq}_{\text{int}}$ are the changes in standard molar Gibbs free energy, enthalpy, and entropy between the reactants and the transition state (TS), respectively.

4. Catalytic Reaction Tests

The catalytic reaction tests for CO_2 hydrogenation were carried out in a stainless steel tubular fixed-bed reactor with an inner diameter of 10 mm. 0.6 g of InZrO$_x$-Beta composite catalyst (20–40 mesh) was placed in the middle of tube reactor. The catalyst sample was first pretreated with H_2 flow (30 mL min^{-1}) at 400 °C for 2 h. After the reactor temperature was decreased to 315 °C, the H_2 and CO_2 mixture with an H_2/CO_2 molar volume ratio of 3 was introduced into the reactor. The reaction was carried out at 315 °C, 3.0 MPa, and GHSV of 1200 mL g^{-1} h^{-1}, unless otherwise specified. The effluents were analyzed online by an Agilent 7890A gas chromatograph (GC) equipped with one TCD, two flame ionization detectors (FID), and two capillary columns (J&W 127–7031, 30 m × 530 μm × 0.25 μm; Agilent 19095P-S25, 50 m × 530 μm × 15 μm).

The conversion of CO_2 (X_{CO_2}) and selectivity to CO (S_{CO}) were calculated as follows:

$$X_{CO_2} = \left(N_{CO_2,in} - N_{CO_2,out}\right) / N_{CO_2,in} \times 100\% \quad (2)$$

$$S_{CO} = N_{CO,out} / \left(N_{CO_2,in} - N_{CO_2,out}\right) \times 100\% \quad (3)$$

where $N_{CO_2,in}$ is the molar quantity of CO_2 fed into the reactor, while $N_{CO,out}$ and $N_{CO_2,out}$ are

the molar quantities of CO and CO_2, respectively, in the effluent discharged from the reactor.

The selectivities to CH_3OH, DME and C_iH_{ix} (S_{CH_3OH}, S_{DME} and $S_{C_iH_{ix}}$) in the hydrocarbon products were calculated as follows:

$$S_{CH_3OH} = N_{CH_3OH} / \left(N_{CH_3OH} + 2N_{DME} + \sum i \cdot S_{C_iH_{ix}}\right) \times 100\% \tag{4}$$

$$S_{DME} = 2N_{DME} / \left(N_{CH_3OH} + 2N_{DME} + \sum i \cdot N_{C_iH_{ix}}\right) \times 100\% \tag{5}$$

$$S_{C_iH_{ix}} = i \cdot N_{C_iH_{ix}} / \left(N_{CH_3OH} + 2N_{DME} + \sum i \cdot N_{C_iH_{ix}}\right) \times 100\% \tag{6}$$

where N_{CH_3OH}, N_{DME}, and $N_{C_iH_{ix}}$ are the molar quantity of CH_3OH, DME and C_iH_{ix} formed in the reaction, respectively, and i is the number of carbon atoms in one C_iH_{ix} molecule.

For comparison, the reaction of methanol-to-olefins (MTO) was also conducted at 315 °C and atmospheric pressure in a U-type quartz tube having an inner diameter of 4 mm with H_2 as the carrier gas. 300 mg of H-Beta (Si/Al = 40) was placed in the constant temperature zone of the reactor between two quartz sand layers. The zeolite catalyst was first pretreated at 500 °C for 2 h in a H_2 flow (9 mL min^{-1}). Methanol (≥ 99.95%, supplied by Sinopharm Chem) was then pumped via a preheater at 150 °C for gasification into the reactor with a methanol weighted hourly space velocity (WHSV) of 0.05 h^{-1}. The methanol conversion (X_{CH_3OH}) was calculated as follows:

$$X_{CH_3OH} = \left(N_{CH_3OH,in} - N_{CH_3OH,out} - 2N_{DME}\right) / N_{CH_3OH,in} \times 100\% \tag{7}$$

The selectivity to C_iH_{ix} product ($S_{C_iH_{ix}}$) was calculated as follows:

$$S_{C_iH_{ix}} = i \cdot N_{C_iH_{ix}} / \sum i \cdot N_{C_iH_{ix}} \times 100\% \tag{8}$$

where $N_{CH_3OH,in}$ and $N_{CH_3OH,out}$ are the molar quantities of methanol fed into the reactor and unreacted methanol in the product, respectively; N_{DME} and $N_{C_iH_{ix}}$ are the molar quantities of DME and C_iH_{ix} formed in the reaction, respectively, and i is the number of carbon atoms in one C_iH_{ix} molecule.

The reaction tests with co-feeding water were conducted in a fixed-bed reactor equipped a high-pressure plunger pump, following the same procedures as reported by Wu and co-workers.[4]

It should be mentioned that for all catalytic reaction tests, a carbon mole balance of better that 95% was achieved.

Supplementary Figures

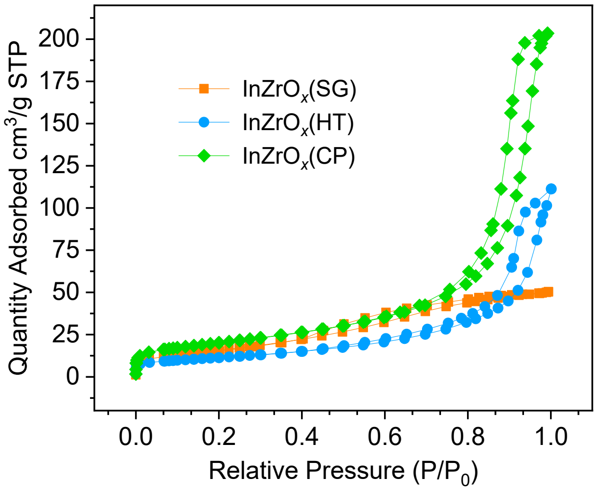

Supplementary Fig. 1. Texture properties. N_2 sorption isotherms of various $InZrO_x$ oxides.

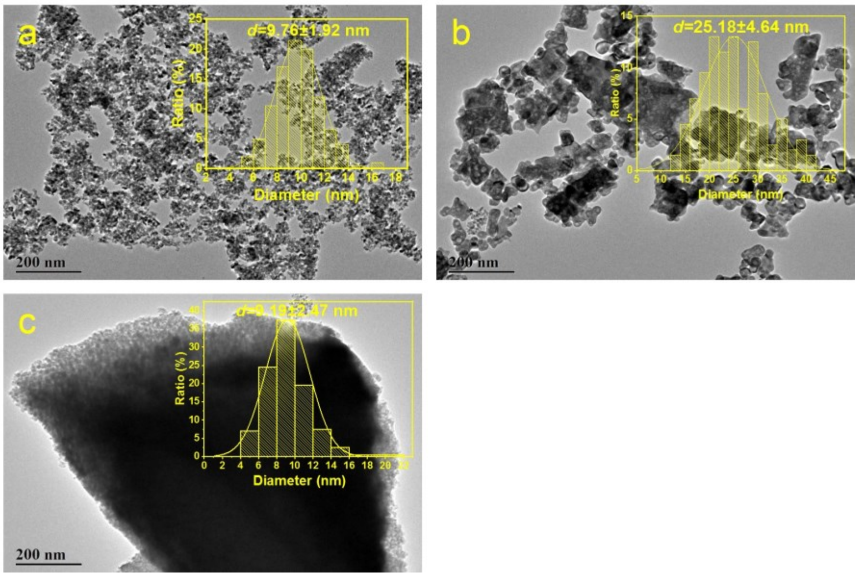

Supplementary Fig. 2. Catalyst morphology. TEM images and the corresponding particle size distributions estimated by counting 200 particles of $InZrO_x$(CP) (**a**), $InZrO_x$(HT) (**b**), and $InZrO_x$(SG) (**c**).

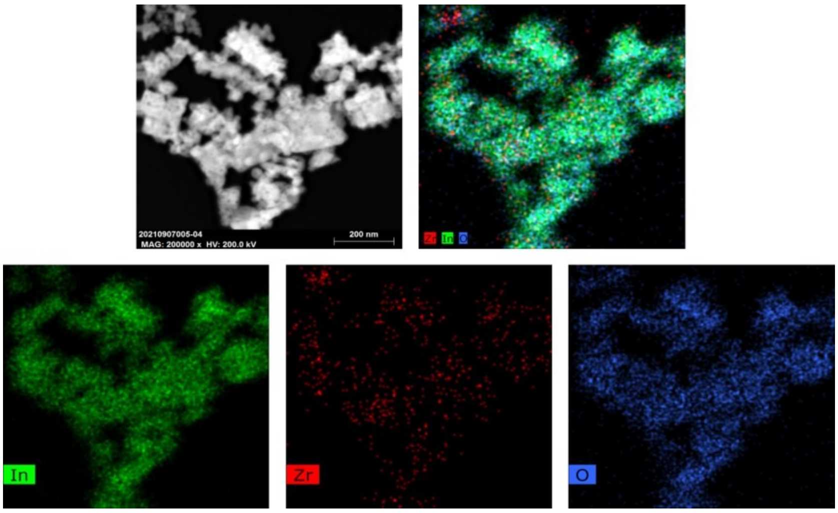

Supplementary Fig. 3. Morphology and elemental distribution. STEM-EDX elemental mapping of $InZrO_x$(HT).

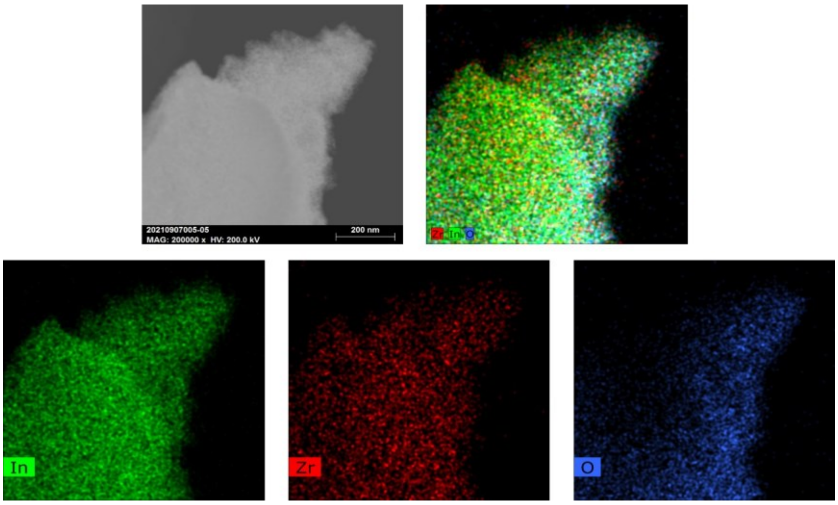

Supplementary Fig. 4. Morphology and elemental distribution. STEM-EDX elemental mapping of $InZrO_x$(SG).

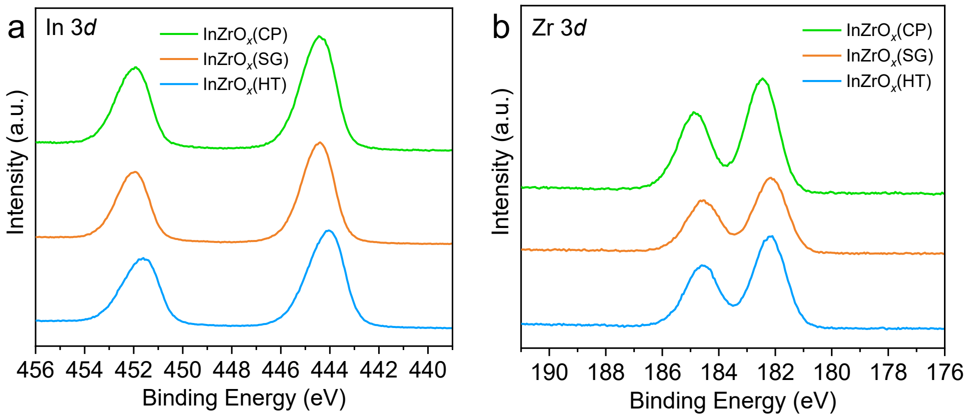

Supplementary Fig. 5. Surface electronic properties. In 3*d* (**a**) and Zr 3*d* (**b**) XPS spectra of various $InZrO_x$ oxides.

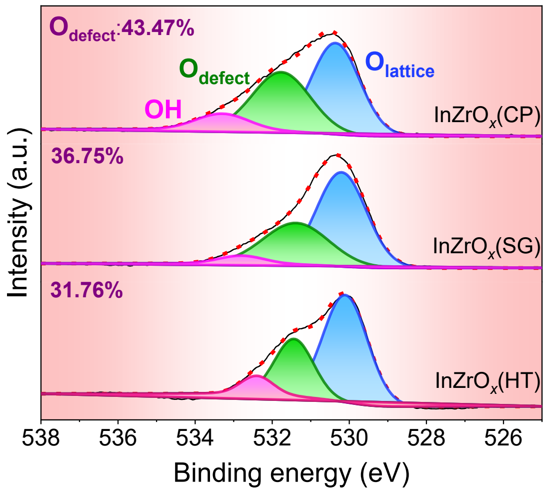

Supplementary Fig. 6. Concentration of surface oxygen vacancies. In situ O 1*s* XPS spectra of various $InZrO_x$ oxides after holding at 400 °C in H_2 atmosphere for 2 h.

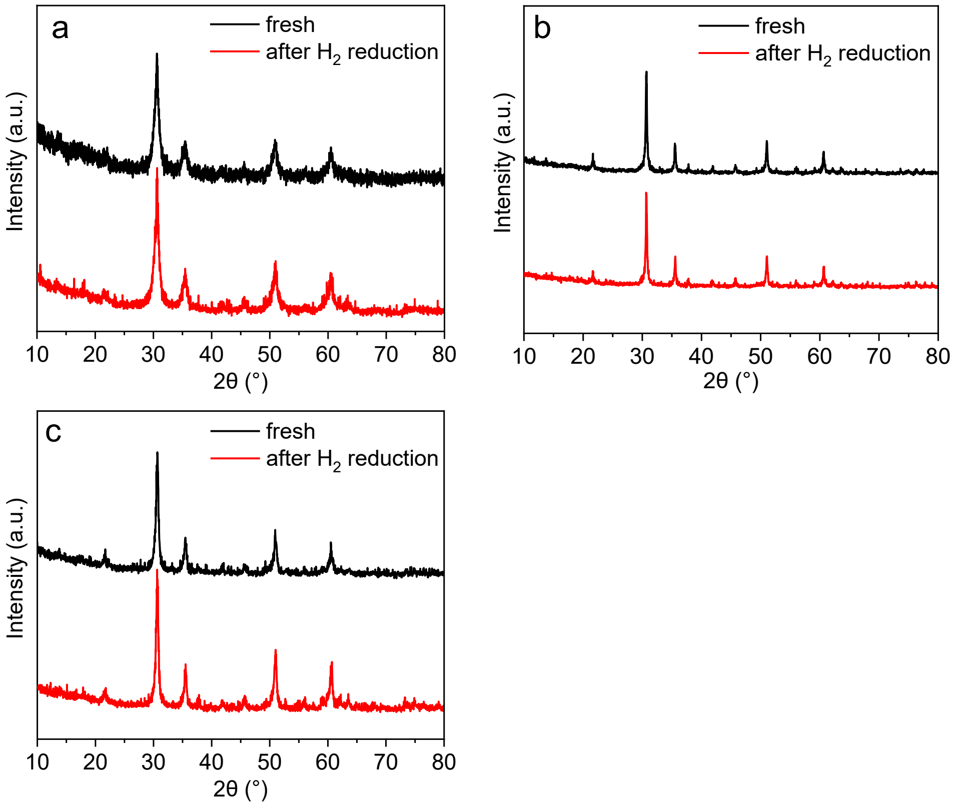

Supplementary Fig. 7. Crystal structure of various InZrO$_x$ composite oxides. In situ XRD patterns of InZrO$_x$(SG) (**a**), InZrO$_x$(HT) (**b**) and InZrO$_x$(CP) (**c**) at 400 °C.

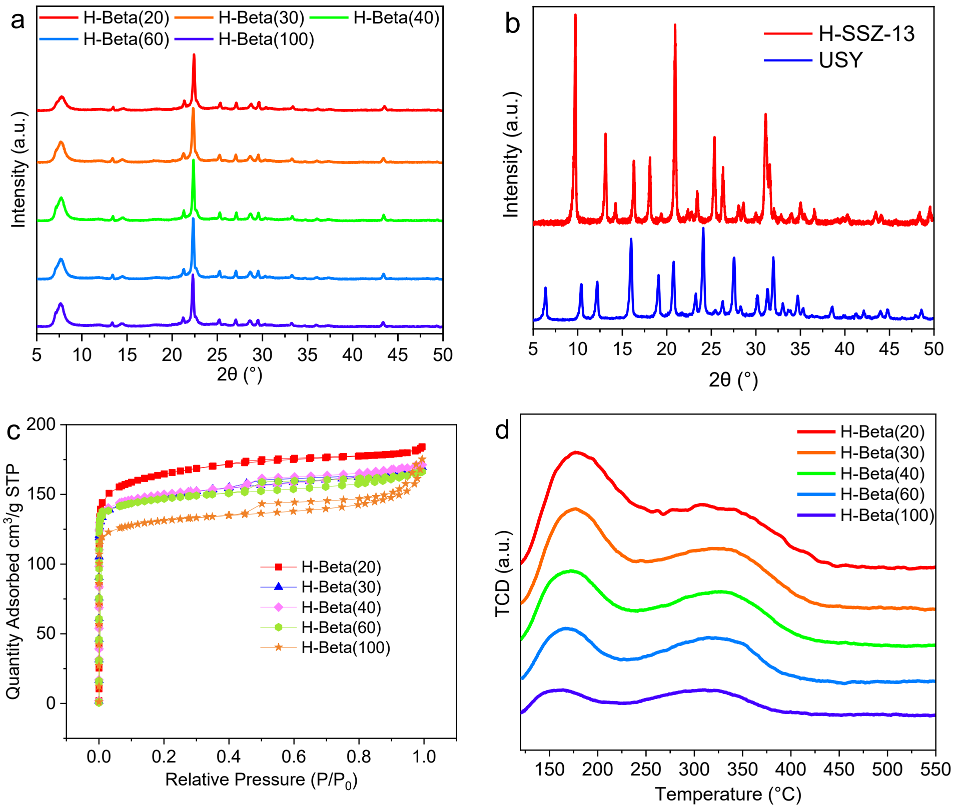

Supplementary Fig. 8. Crystal structure and acidic properties of various zeolites. XRD patterns of H-Beta with different Si/Al ratios (**a**) and H-SSZ-13 and USY (**b**), N_2 sorption isotherms (**c**) and NH_3-TPD profiles (**d**) of H-Beta zeolites with different Si/Al ratios.

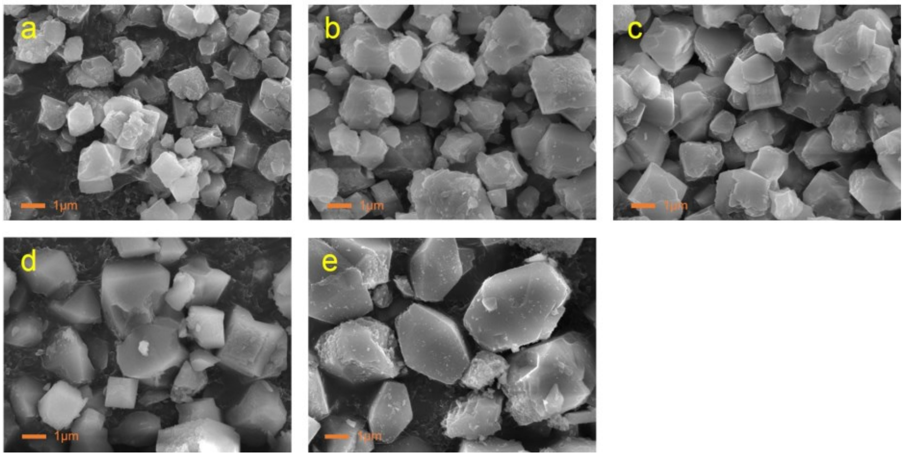

Supplementary Fig. 9. Morphology of various H-Beta zeolites. SEM images of H-Beta with different Si/Al ratios: H-Beta(20) (**a**), H-Beta(30) (**b**), H-Beta(40) (**c**), H-Beta(60) (**d**), and H-Beta(100) (**e**).

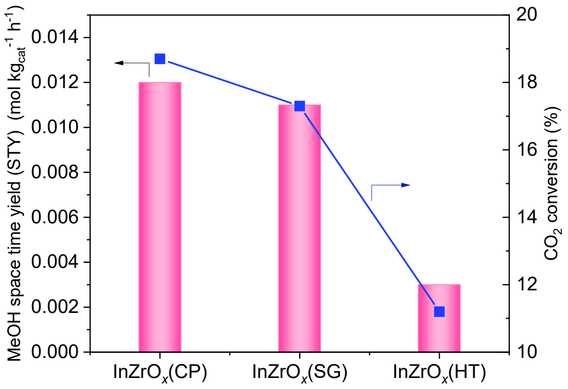

Supplementary Fig. 10. Catalytic performance of $InZrO_x$ oxides in the hydrogenation of CO_2 to methanol. CO_2 conversion and methanol space time yield (STY) for the CO_2 hydrogenation to methanol over various $InZrO_x$ oxides at 24 h on stream. Reaction conditions: 315 °C, 3.0 MPa, 2400 mL g_{cat}^{-1} h^{-1} and H_2/CO_2 = 3:1.

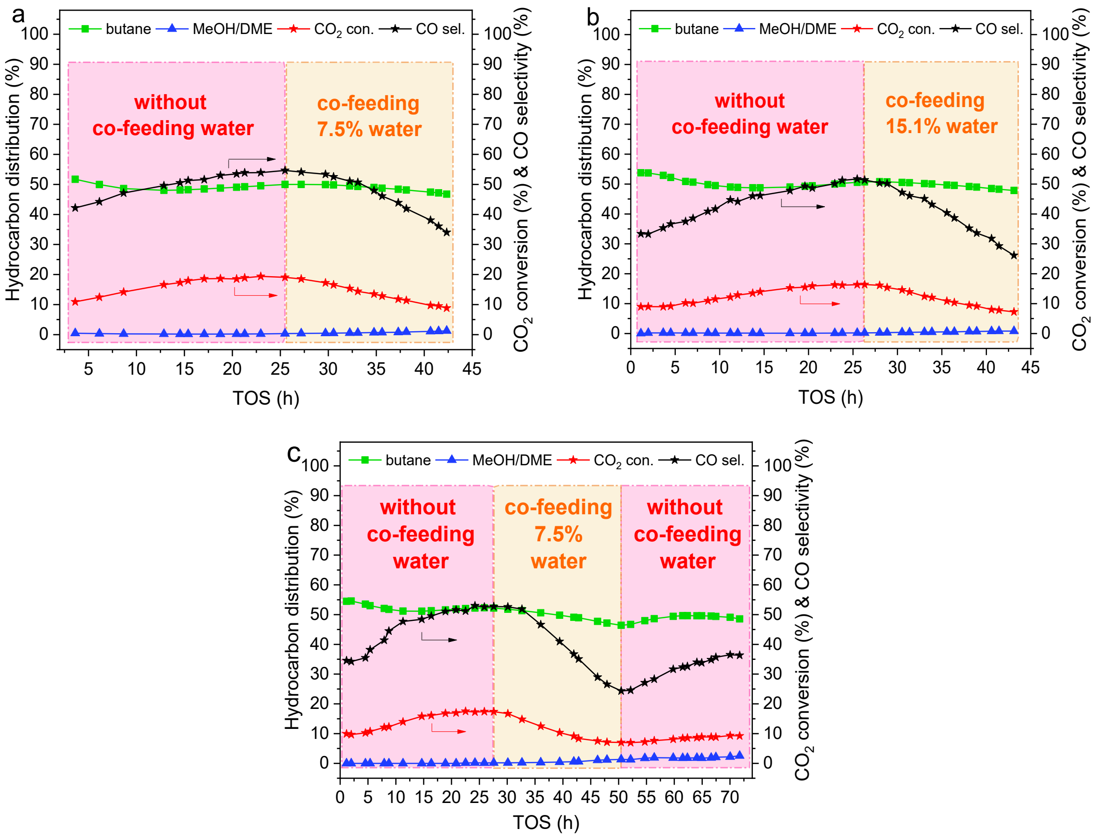

Supplementary Fig. 11. Catalytic performance of InZrO$_x$-Beta in the CO_2 hydrogenation with co-feeding water. Influence of co-feeding water on the catalytic performance of InZrO$_x$(CP)-Beta(40) in the CO_2 hydrogenation. Reaction conditions: 315 °C, 3.0 MPa, 1200 mL g^{-1} h^{-1} and H_2/CO_2 = 3. The content of co-feeding water in the feed is 7.5% (**a, c**) and 15.1 % (**b**).

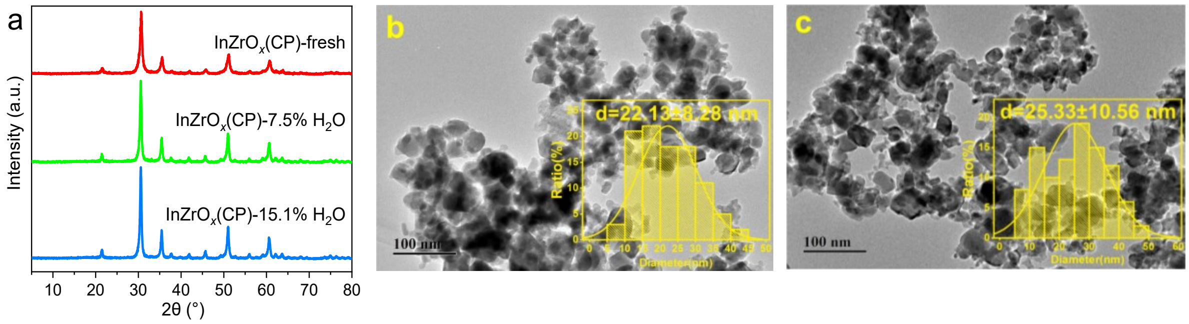

Supplementary Fig. 12. Crystal structure and morphology of the spent InZrO*x*(CP) oxide. XRD patterns (**a**), TEM images, and the corresponding particle size distributions estimated by counting 100 particles of the spent InZrO*x*(CP) oxide after the CO_2 hydrogenation with co-feeding different contents of water: (**b**) co-feeding 7.5% water (InZrO*x*(CP)-7.5%H_2O); (**c**) co-feeding 15.1% water (InZrO*x*(CP)-15.1%H_2O).

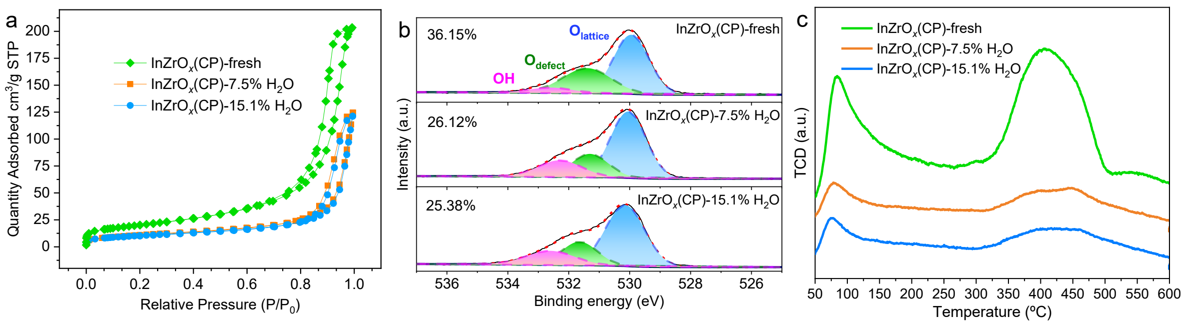

Supplementary Fig. 13. Texture properties and surface oxygen defects of the spent InZrO*x*(CP) oxide. N_2 sorption isotherms (**a**), O 1*s* XPS spectra (**b**), and CO_2-TPD profiles (**c**) of InZrO*x*(CP)-fresh, InZrO*x*(CP)-7.5% H_2O, and InZrO*x*(CP)-15.1% H_2O.

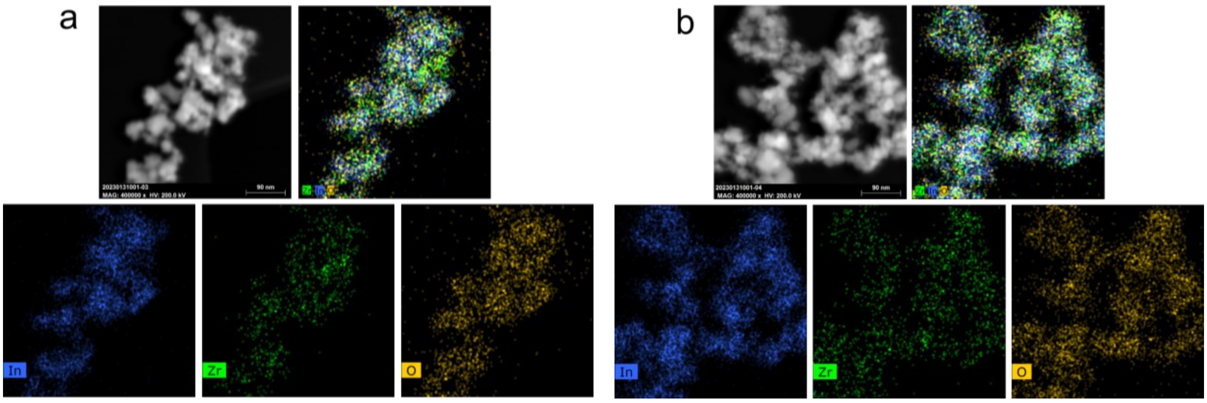

Supplementary Fig. 14. Elemental distributions. STEM-EDX elemental mapping of the spent $InZrO_x$(CP) oxide after the CO_2 hydrogenation with co-feeding 7.5% (**a**) and 15.1% (**b**) water.

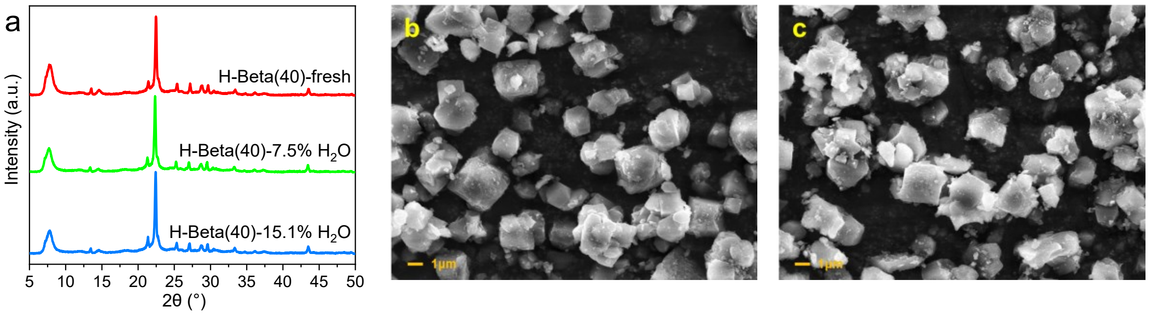

Supplementary Fig. 15. Crystal structure and morphology of the spent H-Beta zeolite. XRD patterns (**a**) and SEM images (**b,c**) of the fresh and spent H-Beta(40) zeolites after the CO_2 hydrogenation with co-feeding different contents of water. (**b**) co-feeding 7.5% water; (**c**) co-feeding 15.1% water.

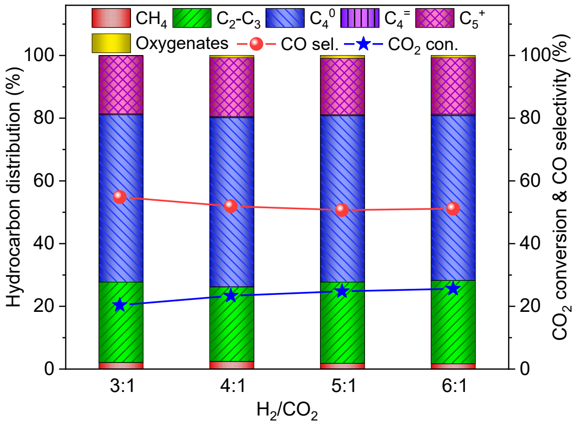

Supplementary Fig. 16. Catalytic performance of InZrO$_x$-Beta in CO_2 hydrogenation, influence of H_2/CO_2 ratio. Influence of the H_2/CO_2 ratio on the catalytic performance of the InZrO$_x$(CP)-Beta(40) composite catalyst in the CO_2 hydrogenation to butane. Reaction conditions: 315 °C, 3.0 MPa, and 1200 mL g_{cat}^{-1} h^{-1}.

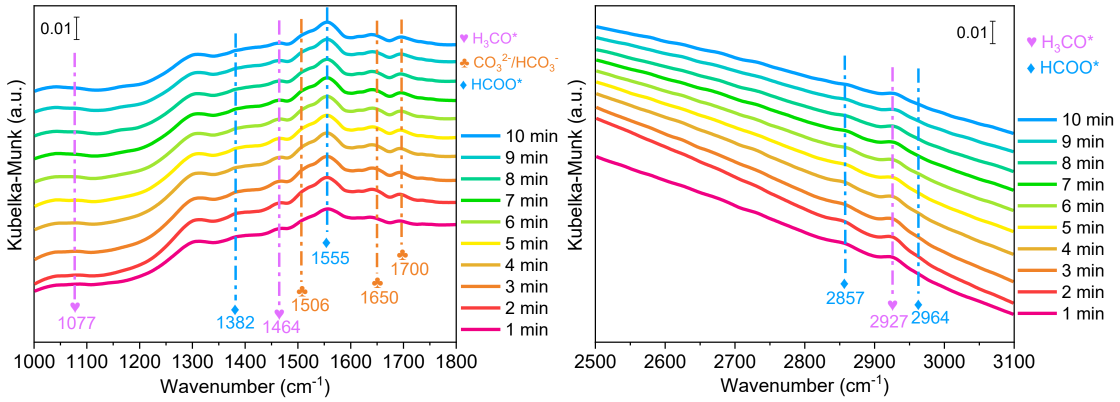

Supplementary Fig. 17. In situ DRIFT spectra. Time-dependent in situ DRIFT spectra for the hydrogenation of CO_2 on InZrO$_x$(SG).

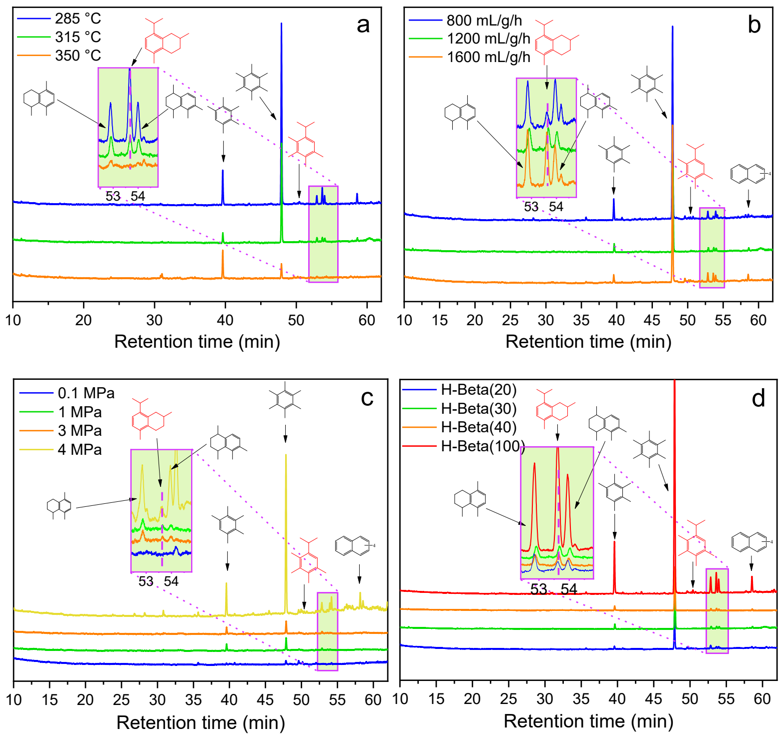

Supplementary Fig. 18. Retained species on the spent InZrO$_x$-Beta catalysts. GC-MS chromatogram of the residual species in the H-Beta zeolites separated from the spent InZrO$_x$(CP)-Beta composite catalysts after catalyzing the CO_2 hydrogenation reaction under different conditions: (**a**) InZrO$_x$(CP)-Beta(40), under different temperatures; (**b**) InZrO$_x$(CP)-Beta(40), under different space velocities; (**c**) InZrO$_x$(CP)-Beta(40), under different pressures; (**d**) InZrO$_x$(CP)-Beta(m), InZrO$_x$(CP) composed with H-Beta(m) of different Si/Al ratios (m = 20, 30, 40 and 100). General reaction conditions of 315 °C, 3.0 MPa, and 1200 mL g^{-1} h^{-1} are used unless specially declared; the shadow inserts are the enlargement of GC-MS chromatograms at a retention time of 52.5–55.0 min.

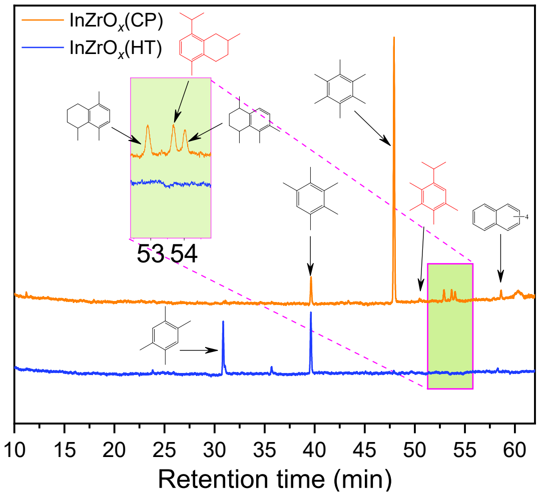

Supplementary Fig. 19. Retained species on the spent InZrO$_x$-Beta catalysts. GC-MS chromatogram of the residual species in the H-Beta zeolite separated from the spent InZrO$_x$(CP)-Beta(40) and InZrO$_x$(HT)-Beta(40) composite catalysts after catalyzing the CO_2 hydrogenation (The shadow insert is the enlargement of GC-MS chromatogram at a retention time of 52.5–55.0 min).

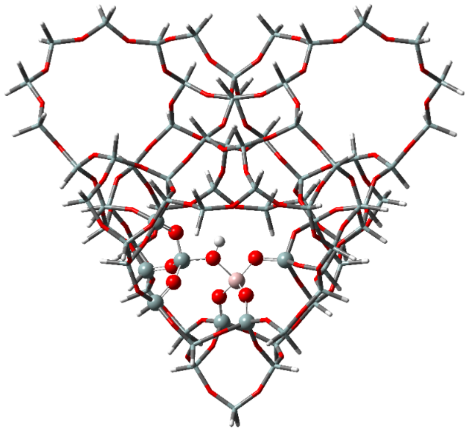

Supplementary Fig. 20. Cluster model of H-Beta. 73T cluster model of H-Beta zeolite. Atom coloring: cyan (Si), red (O), white (H), and pink (Al).

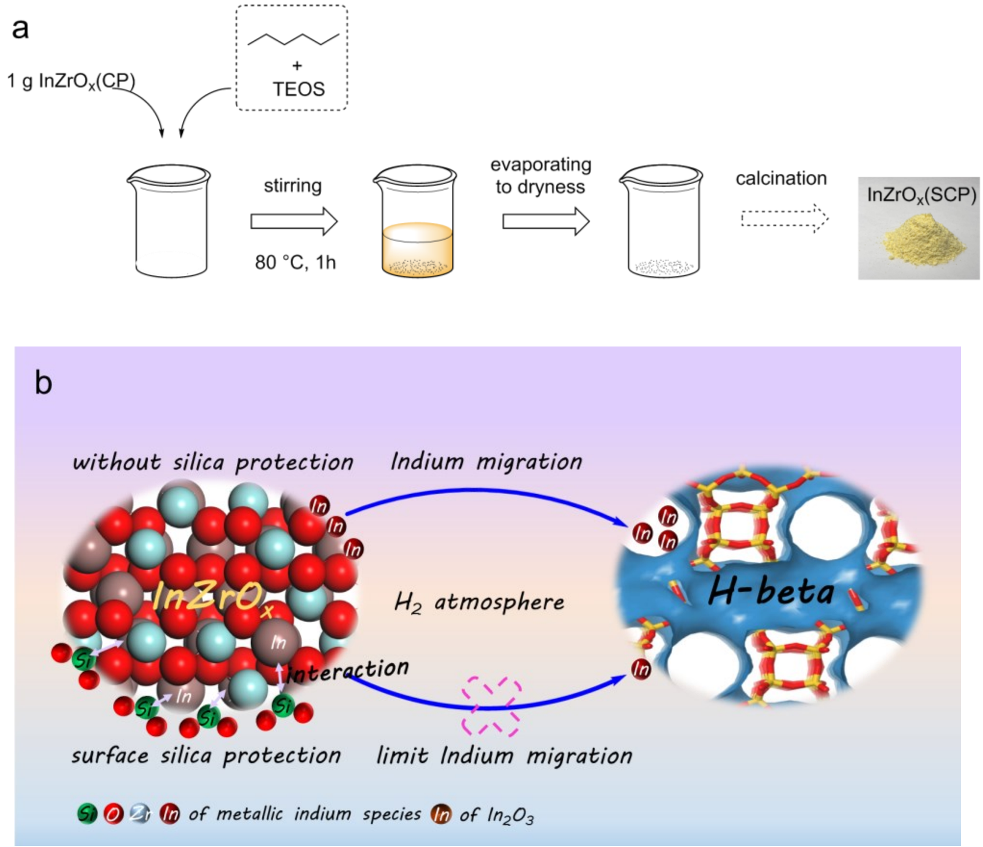

Supplementary Fig. 21. Surface silica protection strategy. Preparation process diagram of silica modified InZrO$_x$(CP) (**a**) and schematic diagram of the function of silica modification in inhibiting the migration of indium species (**b**).

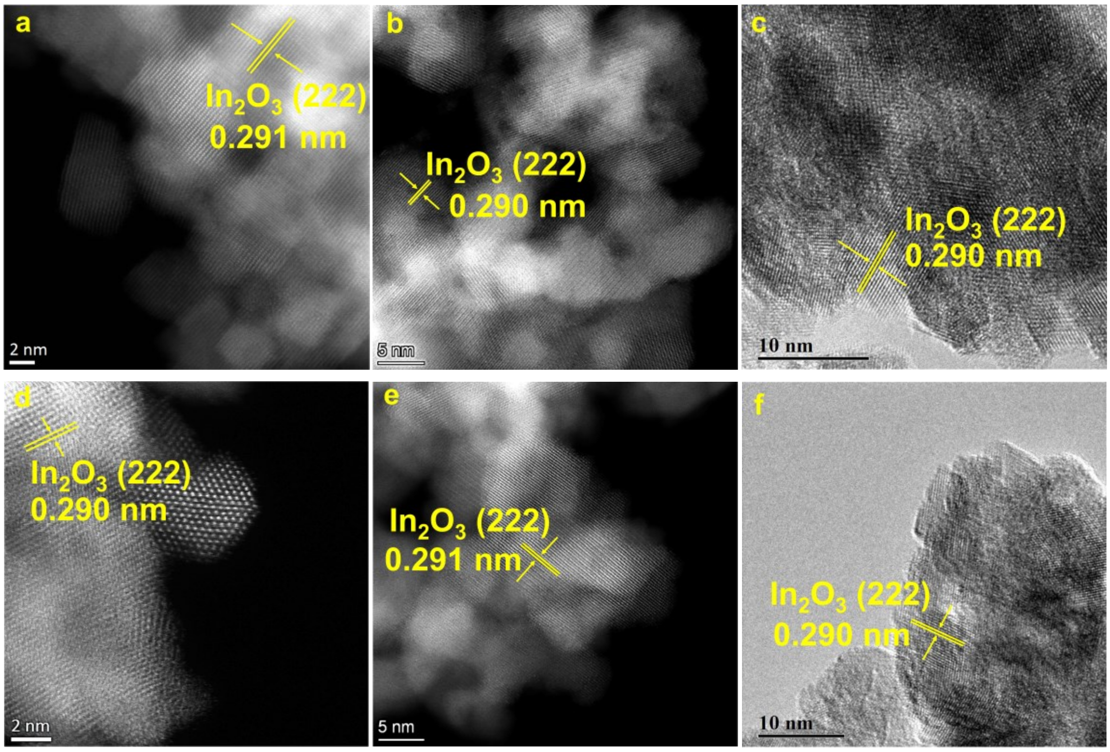

Supplementary Fig. 22. Crystal structure and morphology of various InZrO$_x$ catalysts prepared with surface silica protection strategy. Aberration-corrected HAADF-STEM images of InZrO$_x$(SCP-4) (**a,b**) and InZrO$_x$(SCP-8) (**d–e**); HRTEM images of InZrO$_x$(SCP-4) (**c**) and InZrO$_x$(SCP-8) (**f**).

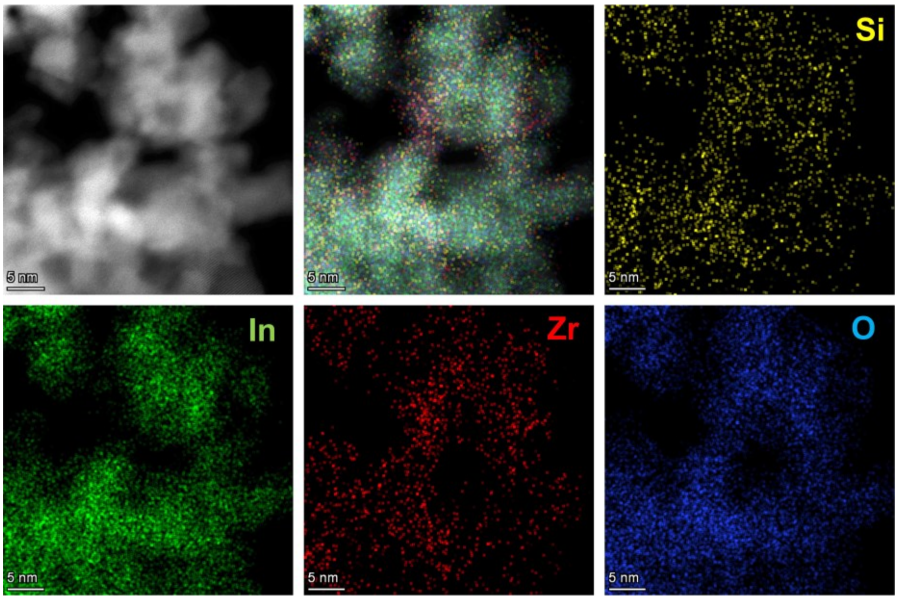

Supplementary Fig. 23. Elemental distribution. STEM and energy-dispersive X-ray (EDX) mapping images of Si (yellow), In (green), Zr (red) and O (blue) elements in $InZrO_x$(SCP-4).

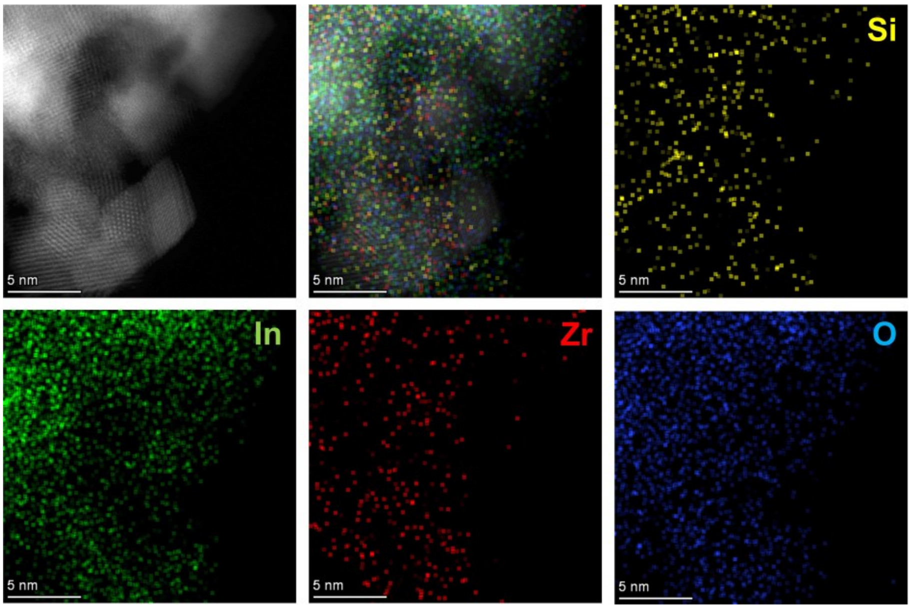

Supplementary Fig. 24. Elemental distribution. STEM and energy-dispersive X-ray (EDX) mapping images of Si (yellow), In (green), Zr (red) and O (blue) elements in $InZrO_x$(SCP-8).

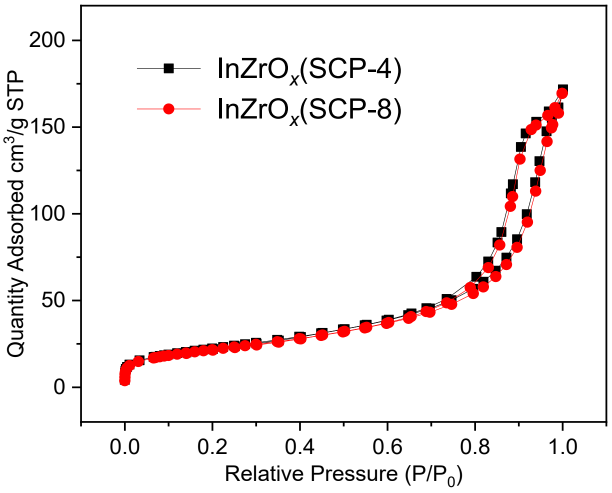

Supplementary Fig. 25. Texture properties. N_2 sorption isotherms of InZrO$_x$(CP), InZrO$_x$(SCP-4) and InZrO$_x$(SCP-8).

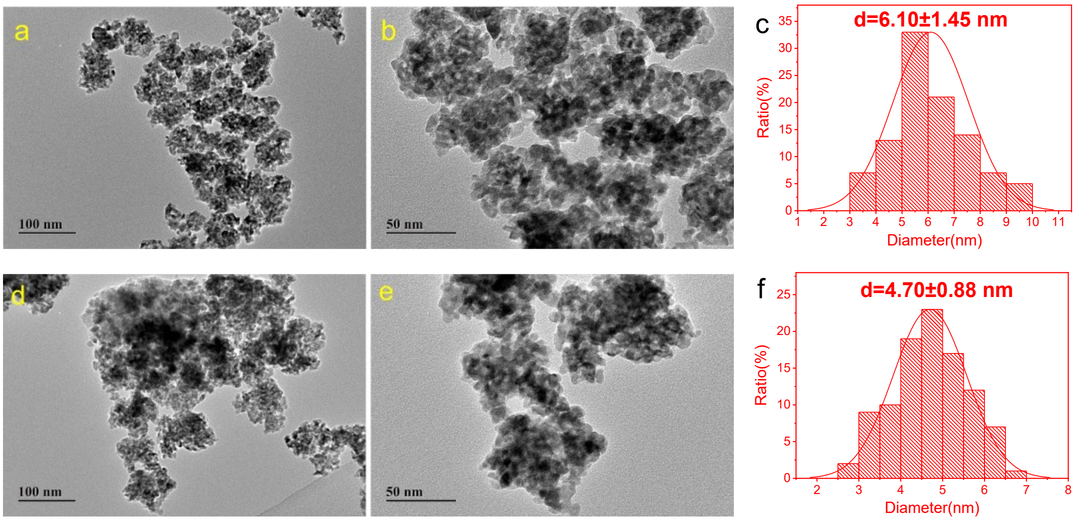

Supplementary Fig. 26. Morphology and particle size distribution. TEM images and corresponding particle size distributions estimated by counting 100 particles of $InZrO_x$(SCP-4) (**a**–**c**) and $InZrO_x$(SCP-8) (**d**–**f**).

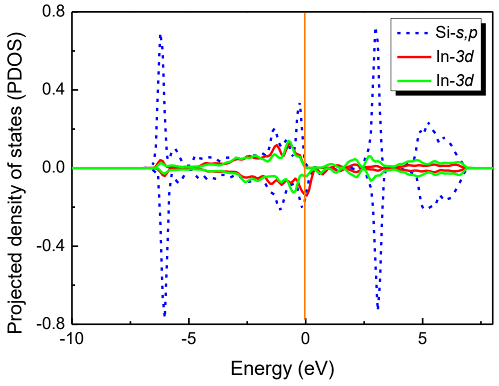

a

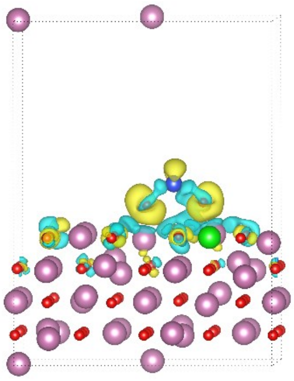

b

Supplementary Fig. 27. Projected density of states and charge difference density. Projected density of states (PDOS) of Si and In atoms (**a**) and charge difference density (CDD) (**b**) for the surface silica-modified $InZrO_x$(CP) oxide. The accumulation and depletion charge regions are shown in yellow and cyan, respectively, in the CDD plot.

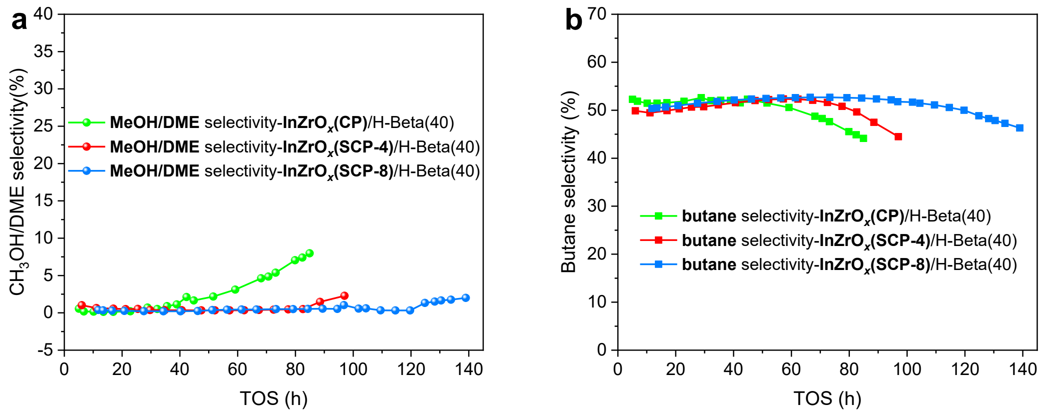

Supplementary Fig. 28. Catalytic performance of InZrO*x*-Beta in the CO_2 hydrogenation. Selectivity to CH_3OH/DME (**a**) and butane (**b**) over the InZrO*x*(CP)-Beta(40), InZrO*x*(SCP-4)-Beta(40) and InZrO*x*(SCP-8)-Beta(40) composite catalysts. Reaction conditions: 315 °C, 3.0 MPa, 1200 mL g^{-1} h^{-1}, and H_2/CO_2 = 6.

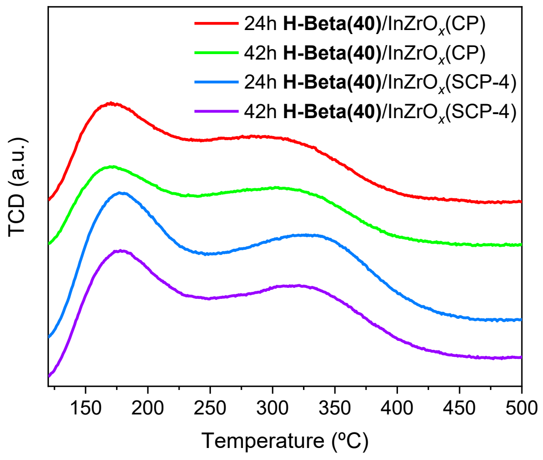

Supplementary Fig. 29. Acidic properties of the spent H-Beta zeolites. NH_3-TPD profiles of the H-Beta zeolites separated from the spent InZrO$_x$(CP)-Beta(40) and InZrO$_x$(SCP-4)-Beta(40) composite catalysts after carrying out the CO_2 hydrogenation reaction for 24 and 42 h.

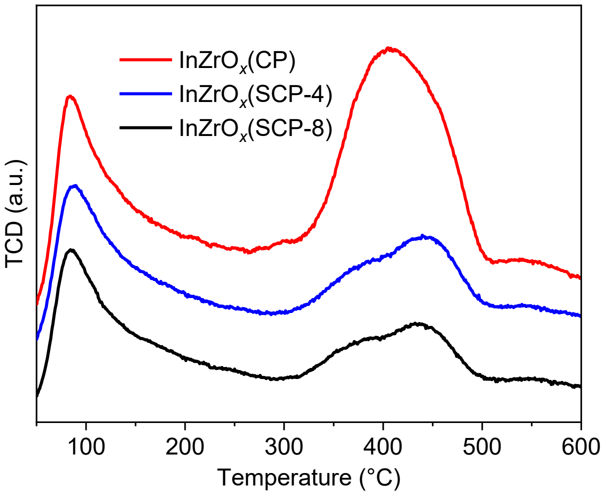

Supplementary Fig. 30. CO_2 adsorption properties. CO_2-TPD profiles of InZrO$_x$(CP), InZrO$_x$(SCP-4) and InZrO$_x$(SCP-8).

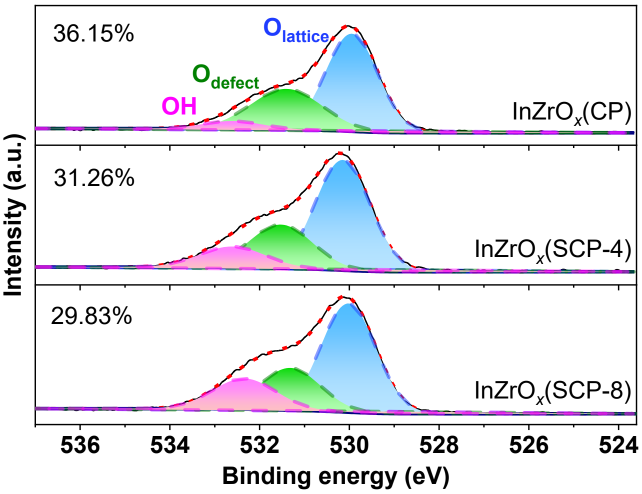

Supplementary Fig. 31. Concentration of surface oxygen vacancies. O 1*s* XPS of $InZrO_x$(CP), $InZrO_x$(SCP-4) and $InZrO_x$(SCP-8).

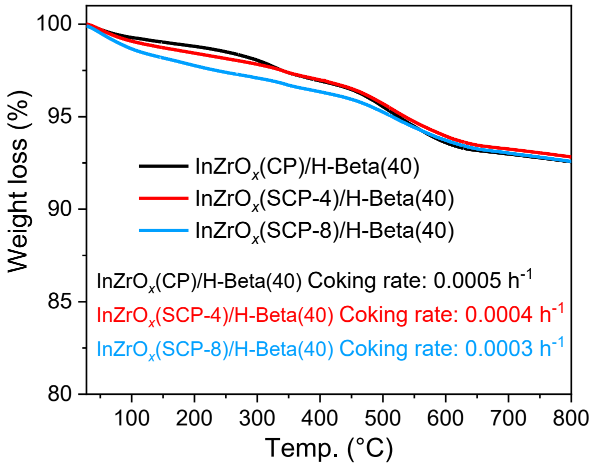

Supplementary Fig. 32. Coke deposition on the spent InZrO$_x$-Beta catalysts. TGA curves of the spent InZrO$_x$(CP)-Beta(40), InZrO$_x$(SCP-4)-Beta(40) and InZrO$_x$(SCP-8)-Beta(40) catalysts after the CO_2 hydrogenation reaction test.

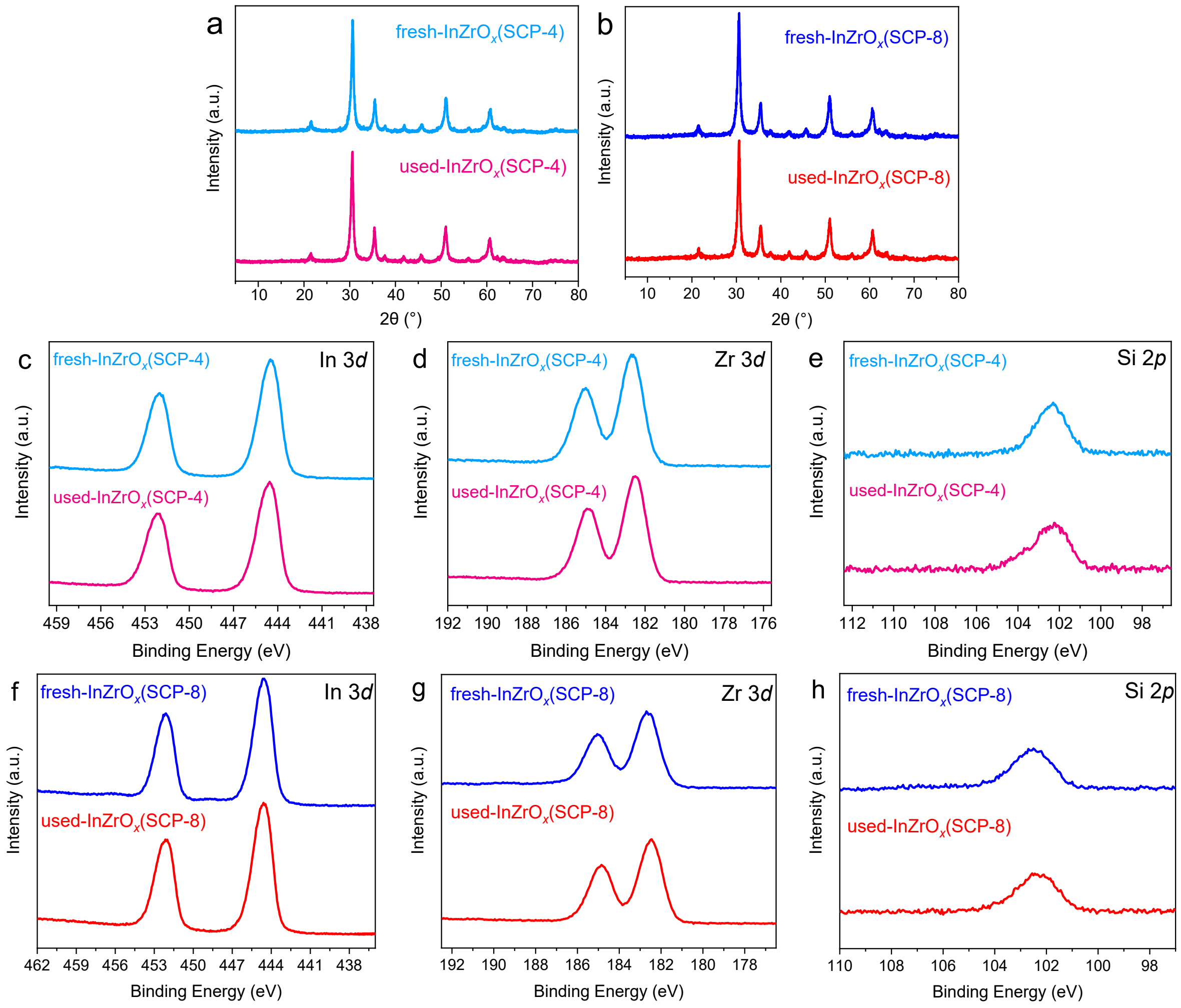

Supplementary Fig. 33. Crystal structure and surface electronic properties. XRD patterns (**a**) and In 3*d* (**c**), Zr 3*d* (**d**) and Si 2*p* (**e**) XPS of the fresh and spent $InZrO_x$(SCP-4) oxides after carrying out the CO_2 hydrogenation for 100 h; XRD patterns (**b**) and In 3*d* (**f**), Zr 3*d* (**g**) and Si 2*p* (**h**) XPS of the fresh and spent $InZrO_x$(SCP-8) oxides after conducting the CO_2 hydrogenation reaction for 140 h.

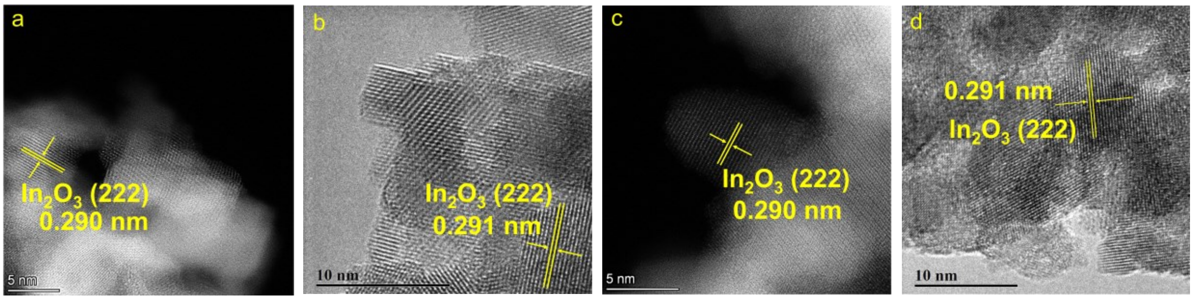

Supplementary Fig. 34. Crystal structure and morphology. Aberration-corrected HAADF-STEM and HR-TEM images of the spent InZrO$_x$(SCP-4) oxide after conducting the CO_2 hydrogenation reaction for 100 h (**a,b**) and the spent InZrO$_x$(SCP-8) oxide after conducting the CO_2 hydrogenation reaction for 140 h (**c,d**).

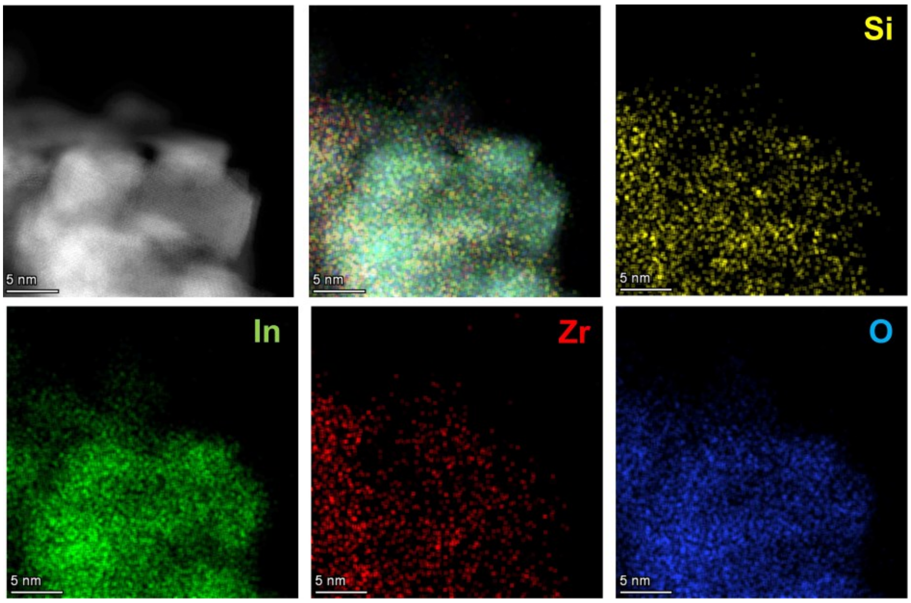

Supplementary Fig. 35. Elemental distributions. STEM and energy-dispersive X-ray (EDX) elemental mapping of Si (yellow), In (green), Zr (red) and O (blue) of the spent $InZrO_x$(SCP-4) oxide after carrying out the CO_2 hydrogenation reaction for 100 h.

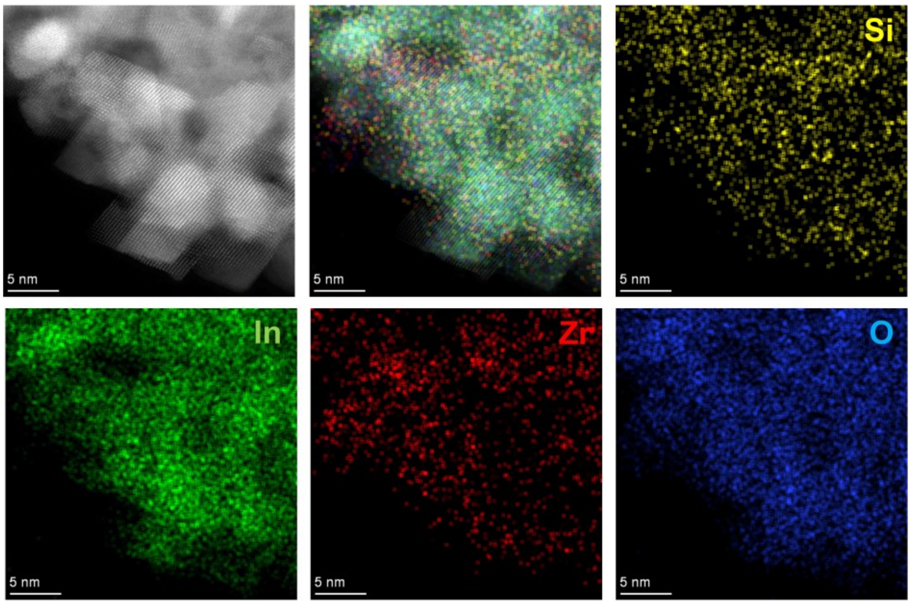

Supplementary Fig. 36. Elemental distributions. STEM and energy-dispersive X-ray (EDX) elemental mapping of Si (yellow), In (green), Zr (red) and O (blue) of the spent $InZrO_x$(SCP-8) oxide after carrying out the CO_2 hydrogenation reaction for 140 h.

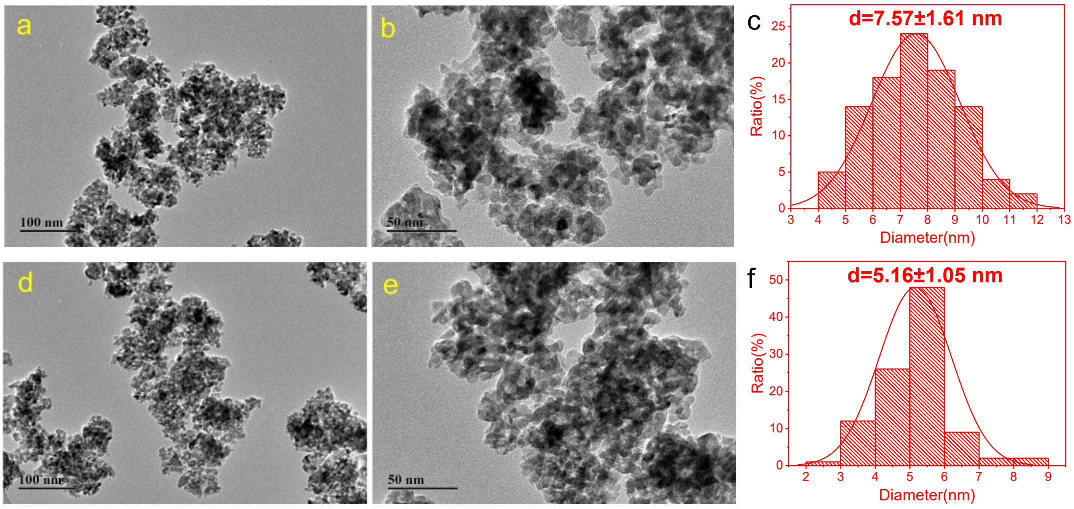

Supplementary Fig. 37. Morphology and particle size distribution. TEM images and the corresponding particle size distributions estimated by counting 100 particles of the spent $InZrO_x$(SCP-4) oxide after conducting the CO_2 hydrogenation reaction for 100 h (**a–c**) and the spent $InZrO_x$(SCP-8) oxide after conducting the CO_2 hydrogenation reaction for 140 h (**d–f**).

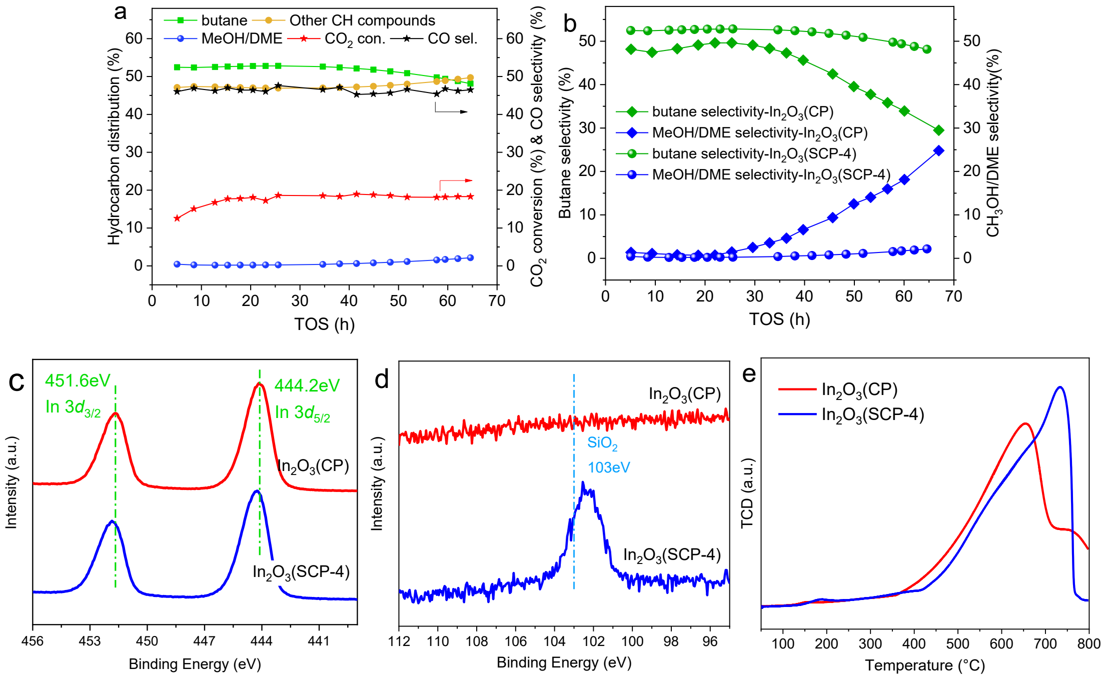

Supplementary Fig. 38. Catalytic performance of In_2O_3-Beta in the CO_2 hydrogenation, related to surface properties. CO_2 conversion and product distribution for the CO_2 hydrogenation to butane over In_2O_3(SCP-4)-Beta(40) (**a**); Selectivity to butane and CH_3OH/DME over In_2O_3(CP)-Beta(40) and In_2O_3(SCP-4)-Beta(40) (**b**). Reaction conditions: 315 °C, 3.0 MPa, 1200 mL g^{-1} h^{-1} and H_2/CO_2 = 6. The catalyst lifetime is defined as the time on stream when the selectivity to unconverted methanol and DME reaches 2% for the CO_2 hydrogenation over the bifunctional catalyst; In 3*d* (**c**) and Si 2*p* (**d**) XPS spectra of fresh In_2O_3(CP) and In_2O_3(SCP-4); H_2-TPR profiles (**e**) of fresh In_2O_3(CP) and In_2O_3(SCP-4).

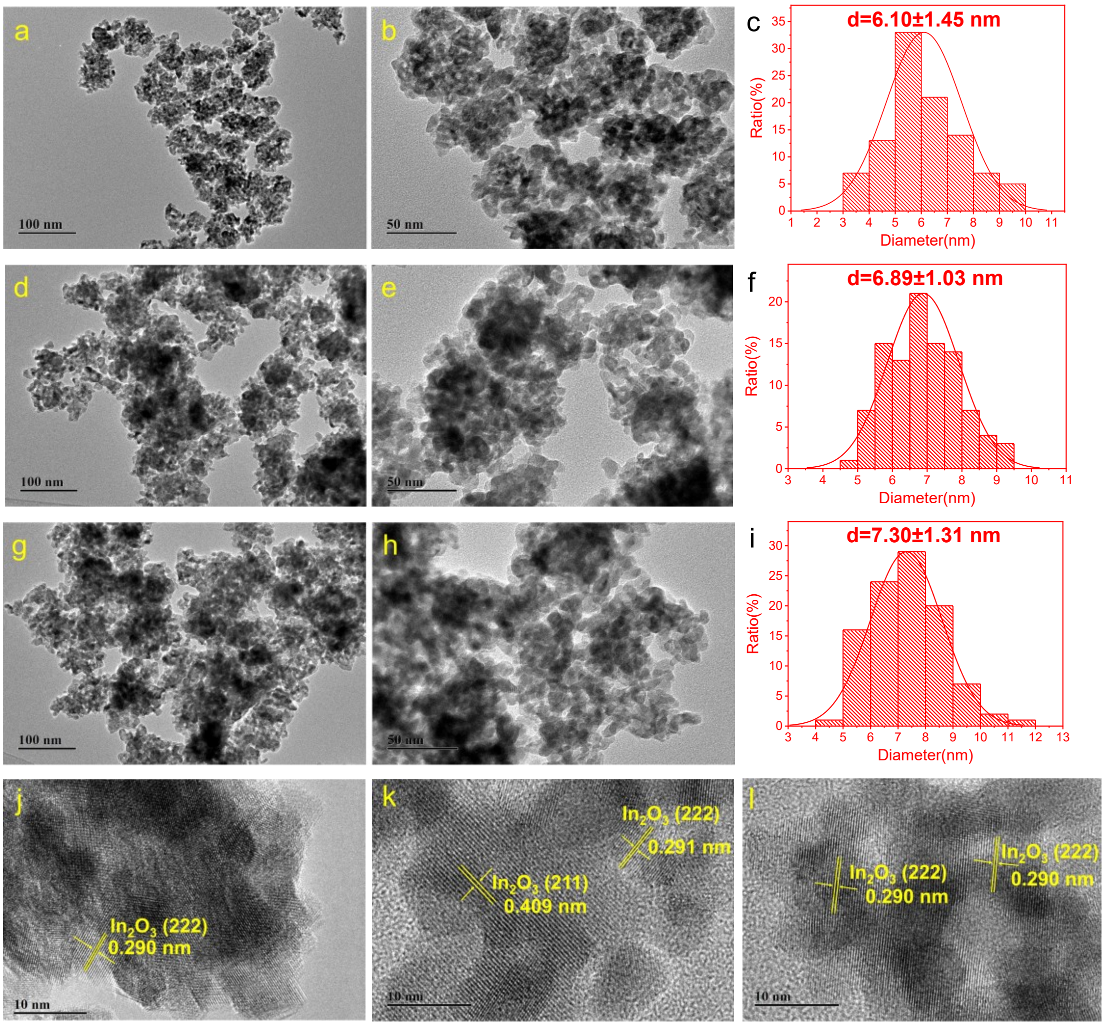

Supplementary Fig. 39. Morphology and particle size distribution. TEM images and the corresponding particle size distributions estimated by counting 100 particles of the spent $InZrO_x$(SCP-4) oxide after reaction for different times: fresh (**a–c**), 24 h (**d–f**) and 42 h (**g–i**); HRTEM image of the spent $InZrO_x$(SCP-4) sample after reaction for different times: fresh (**j**), 24 h (**k**) and 42 h (**l**).

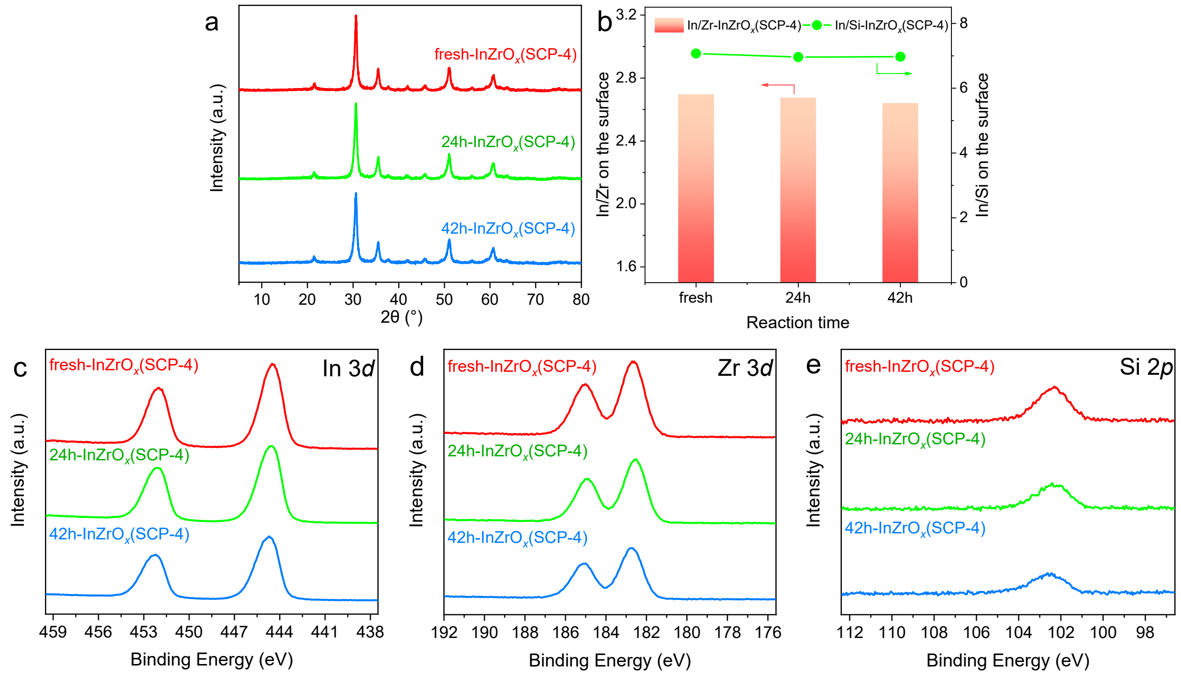

Supplementary Fig. 40. Crystal structure and surface electronic properties. XRD patterns (**a**) of $InZrO_x$ separated from the spent $InZrO_x$(SCP-4)-Beta(40) composite catalysts after reaction for different times; Surface In/Zr and In/Si ratios of the spent $InZrO_x$(SCP-4) oxide after reaction for different times (**b**). The surface In/Zr and In/Si ratios were calculated by the following equations: In/Zr = $(I_{In}/S_{In})/(I_{Zr}/S_{Zr})$, In/Si = $(I_{In}/S_{In})/(I_{Si}/S_{Si})$, where I_E represent peak area of various elements in the XPS spectra and S_E is the corresponding sensitivity factor. The In 3*d* (**c**), Zr 3*d* (**d**) and Si 2*p* (**e**) XPS spectra of $InZrO_x$(SCP-4) separated from the spent $InZrO_x$(SCP-4)-Beta(40) composite catalyst after reaction for different times.

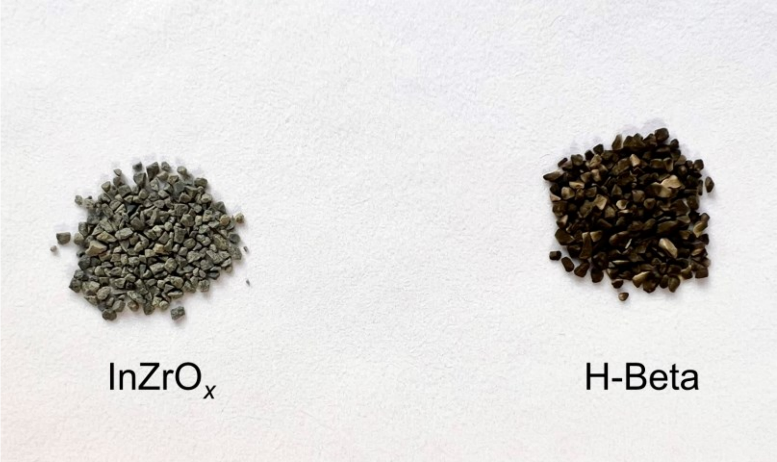

Supplementary Fig. 41. Photographs of InZrO$_x$ and Beta moieties. Photographs of the InZrO$_x$ oxide and H-Beta(40) zeolite components separated from the spent InZrO$_x$-Beta(40) composite catalysts after the CO_2 hydrogenation reaction test.

Supplementary Tables

Supplementary Table 1. Textural properties of various $InZrO_x$ oxides.

Sample	S_{BET}[a] ($m^2\ g^{-1}$)	S_{micro} ($m^2\ g^{-1}$)	V_{micro}[b] ($cm^3\ g^{-1}$)	V_{meso}[c] ($cm^3\ g^{-1}$)
$InZrO_x$(SG)	59	25	0.003	0.07
$InZrO_x$(HT)	41	22	0.006	0.15
$InZrO_x$(CP)	73	29	0.006	0.31

[a] S_{BET}: BET specific surface area.

[b] V_{micro}: micropore volume determined by t-plot.

[c] V_{meso}: mesopore volume determined by $V_{total}-V_{mico}$.

Supplementary Table 2. Textural properties of various H-Beta zeolites with different Si/Al ratios.

Zeolites	Si/Al ratio[a]	S^{b} ($m^2\ g^{-1}$)		V_{pore}^{c} ($cm^3\ g^{-1}$)		D_{acid}^{d} (μmol g^{-1})	
		Total	Micro	Micro	Meso	Weak	Strong
H-Beta(20)	22	510	376	0.18	0.098	544	463
H-Beta(30)	33	459	359	0.17	0.085	355	446
H-Beta(40)	43	460	369	0.18	0.081	245	397
H-Beta(60)	58	450	367	0.18	0.074	154	230
H-Beta(100)	99	402	327	0.16	0.098	66	113

[a] Si/Al ratio: tested by ICP.
[b] Total and micro surface area.
[c] Micropore and mesopore volume.
[d] Acid content, measured by NH_3-TPD.

Supplementary Table 3. Textural properties of the spent $InZrO_x$(CP) oxides after conducting the CO_2 hydrogenation reaction with co-feeding different contents of water.

Sample	S_{BET} (m^2 g^{-1})	S_{micro} (m^2 g^{-1})	V_{micro} (cm^3 g^{-1})	V_{meso} (cm^3 g^{-1})
$InZrO_x$(CP)-fresh	73	29	0.006	0.31
$InZrO_x$(CP)-7.5% H_2O	39	12	0.001	0.18
$InZrO_x$(CP)-15.1% H_2O	37	12	0.001	0.17

Supplementary Table 4. Elemental composition of H-Beta(40) zeolite before and after reaction, measured by ICP.

Sample	Si/Al	In content (wt%)	Zr content (wt%)
fresh H-Beta(40)	41.3	-	-
spent H-Beta(40)	42.0	0.26%	-

Note: The spent H-Beta(40) zeolite represents the H-Beta(40) zeolite component separated from the spent $InZrO_x$-Beta(40) composite catalyst after the CO_2 hydrogenation reaction test.

Supplementary Table 5. Textural properties of $InZrO_x$(CP) oxides with different Si contents.

Sample	S_{BET} (m^2 g^{-1})	S_{micro} (m^2 g^{-1})	V_{micro} (cm^3 g^{-1})	V_{meso} (cm^3 g^{-1})
$InZrO_x$(CP)	73	29	0.006	0.31
$InZrO_x$(SCP-4)	81	27	0.003	0.23
$InZrO_x$(SCP-8)	77	24	0.002	0.24

Supplementary Table 6. Acidic content of various H-Beta(40) zeolite separated from the spent $InZrO_x$(CP)-Beta(40) and $InZrO_x$(SCP-4)-Beta(40) composite catalysts after conducting the CO_2 hydrogenation reaction for 24 and 42 h.

H-Beta(40) zeolite separated from	Acid content (μmol g^{-1})	
	weak	strong
$InZrO_x$(CP)-Beta(40), reaction for 24 h	127	101
$InZrO_x$(CP)-Beta(40), reaction for 42 h	108	86
$InZrO_x$(SCP-4)-Beta(40), reaction for 24 h	230	189
$InZrO_x$(SCP-4)-Beta(40), reaction for 42 h	169	133

Supplementary Table 7. CO_2 conversion and CO selectivity of various indium-based composite catalysts for CO_2 hydrogenation.

Catalyst	CO_2 conversion (%)	CO selectivity (%)	Ref.
In_2O_3/SAPO-34	15.3	68.3	5
In-Zr(16:1)/SAPO-34	17.1	64.0	5
In-Zr(4:1)/SAPO-34	26.2	63.9	5
In-Zr(1:1)/SAPO-34	23.3	68.6	5
In-Zr(1:4)/SAPO-34	22.5	70.4	5
In_2O_3/HZSM-5	13.1	44.8	6
In-Zr-Zn/SAPO-34	~12	~47	7
In_2O_3-$ZnZrO_x$/SAPO-34	17.0	55.8	8
In_2O_3/SAPO-34	~33	~60	9
$InZrO_x$/SSZ-13	~24	~61	10
$InZrO_x$(SCP-4)-Beta(40)	19.7	34.5	This work

Supplementary References

1. Camblor, M. A.; Avelino, C.; Susana, V. Synthesis in fluoride media and characterisation of aluminosilicate zeolite beta. *J. Mater. Chem. A* **8**, 2137–2145 (1998).

2. Li, S.; Cao, J.; Liu, Y.; Feng, X.; Chen, X.; Yang, C. Effect of acid strength on the formation mechanism of tertiary butyl carbocation in initial C_4 alkylation reaction over H-BEA zeolite: A density functional theory study. *Catal. Today* **355**, 171–179 (2020).

3. Pan, Y.; Liu, C.; Shi, P. The cleavage of the methane CH bond over PdO/H-BEA: A density functional theory study. *Appl. Surf. Sci.* **254**, 5587–5593 (2008).

4. Wu, W.; Wang, Y.; Luo, L.; Wang, M.; Li, Z.; Chen, Y.; Wang, Z.; Chai, J.; Cen, Z.; Shi, Y.; Zhao, J.; Zeng, J.; Li, H. CO_2 hydrogenation over copper/ZnO single-atom catalysts: water-promoted transient synthesis of methanol. *Angew. Chem. Int. Ed.* **61**, e202213024 (2022).

5. Dang, S.; Gao, P.; Liu, Z.; Chen, X.; Yang, C.; Wang, H.; Zhong, L.; Li, S.; Sun, Y. Role of zirconium in direct CO_2 hydrogenation to lower olefins on oxide/zeolite bifunctional catalysts. *J. Catal.* **364**, 382–393 (2018).

6. Gao, P.; Li, S.; Bu, X.; Dang, S.; Liu, Z.; Wang, H.; Zhong, L.; Qiu, M.; Yang, C.; Cai, J.; Wei, W.; Sun, Y. Direct conversion of CO_2 into liquid fuels with high selectivity over a bifunctional catalyst. *Nat. Chem.* **9**, 1019–1024 (2017).

7. Gao, P.; Dang, S.; Li, S.; Bu, X.; Liu, Z.; Qiu, M.; Yang, C.; Wang, H.; Zhong, L.; Han, Y.; Liu, Q.; Wei, W.; Sun, Y. Direct production of lower olefins from CO_2 conversion via bifunctional catalysis. *ACS Catal.* **8**, 571–578 (2017).

8. Dang, S.; Li, S.; Yang, C.; Chen, X.; Li, X.; Zhong, L.; Gao, P.; Sun, Y. Selective transformation of CO_2 and H_2 into lower olefins over In_2O_3-$ZnZrO_x$/SAPO-34 bifunctional catalysts. *ChemSusChem* **12**, 3582–3591 (2019).

9. Numpilai, T.; Wattanakit, C.; Chareonpanich, M.; Limtrakul, J.; Witoon, T. Optimization of synthesis condition for CO_2 hydrogenation to light olefins over In_2O_3 admixed with SAPO-34. *Energy Convers. Manage.* **180**, 511–523 (2019).

10. Liu, Z.; Ni, Y.; Sun, T.; Zhu, W.; Liu, Z. Conversion of CO_2 and H_2 into propane over InZrO and SSZ-13 composite catalyst. *J. Energy Chem.* **54**, 111–117 (2021).
