# Supplementary material for: Selective conversion of CO2 to isobutane-enriched C4 alkanes over InZrOx-Beta composite catalyst
Source: Nat Commun. 2023 May 6;14:2627. doi: 10.1038/s41467-023-38336-5 (PMC10164185; doi:10.1038/s41467-023-38336-5)
Supplement: Supplementary file 3 — Source Data [file 41467_2023_38336_MOESM3_ESM.zip › Source_Data_for_Figures_in_Supplementary_Information/Source_Data_Supplementary_Figure_21/Supplementary_Figure_21.pptx]

## Slide 1
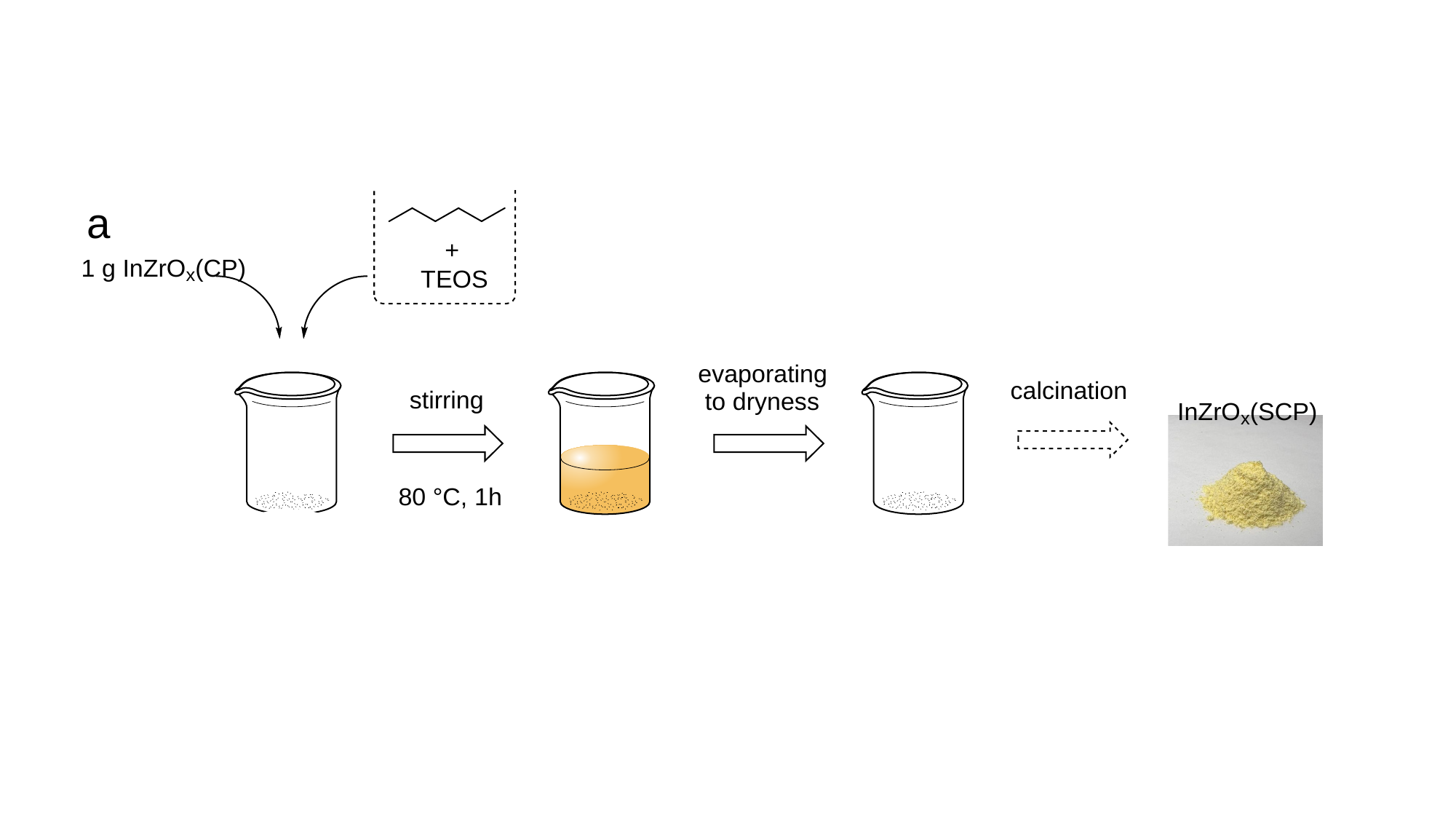

a

## Slide 2
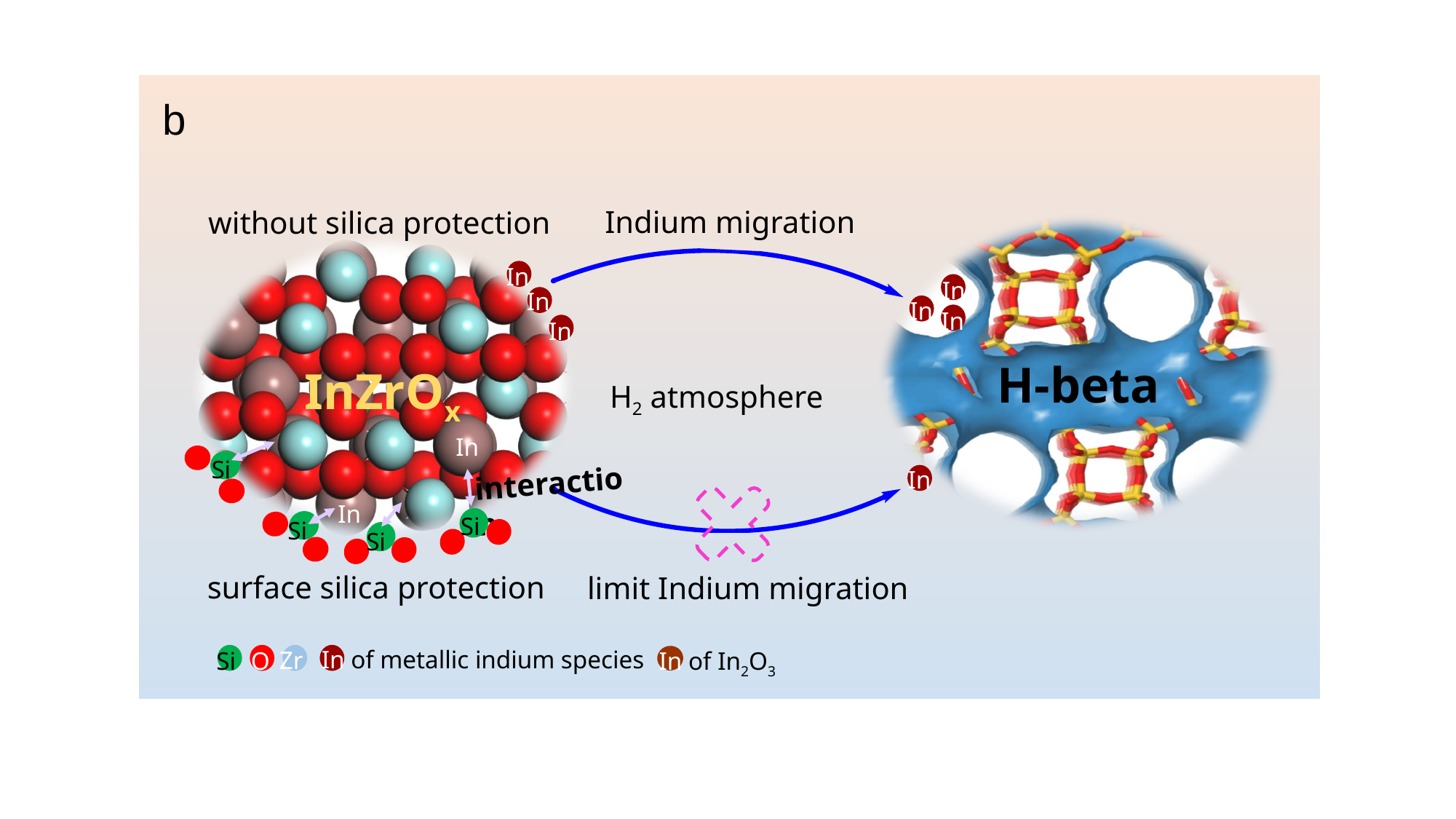

Indium migration
H-beta
InZrOx
H2 atmosphere
In
interaction
limit Indium migration
without silica protection
In
In
In
In
In
In
Si
In
In
Si
Si
Si
surface silica protection
In of metallic indium species
Zr
Si
O
In of In2O3
b
