# Supplementary figures and images for: Selective conversion of CO2 to isobutane-enriched C4 alkanes over InZrOx-Beta composite catalyst
Source: Nat Commun. 2023 May 6;14:2627. doi: 10.1038/s41467-023-38336-5 (PMC10164185; doi:10.1038/s41467-023-38336-5)

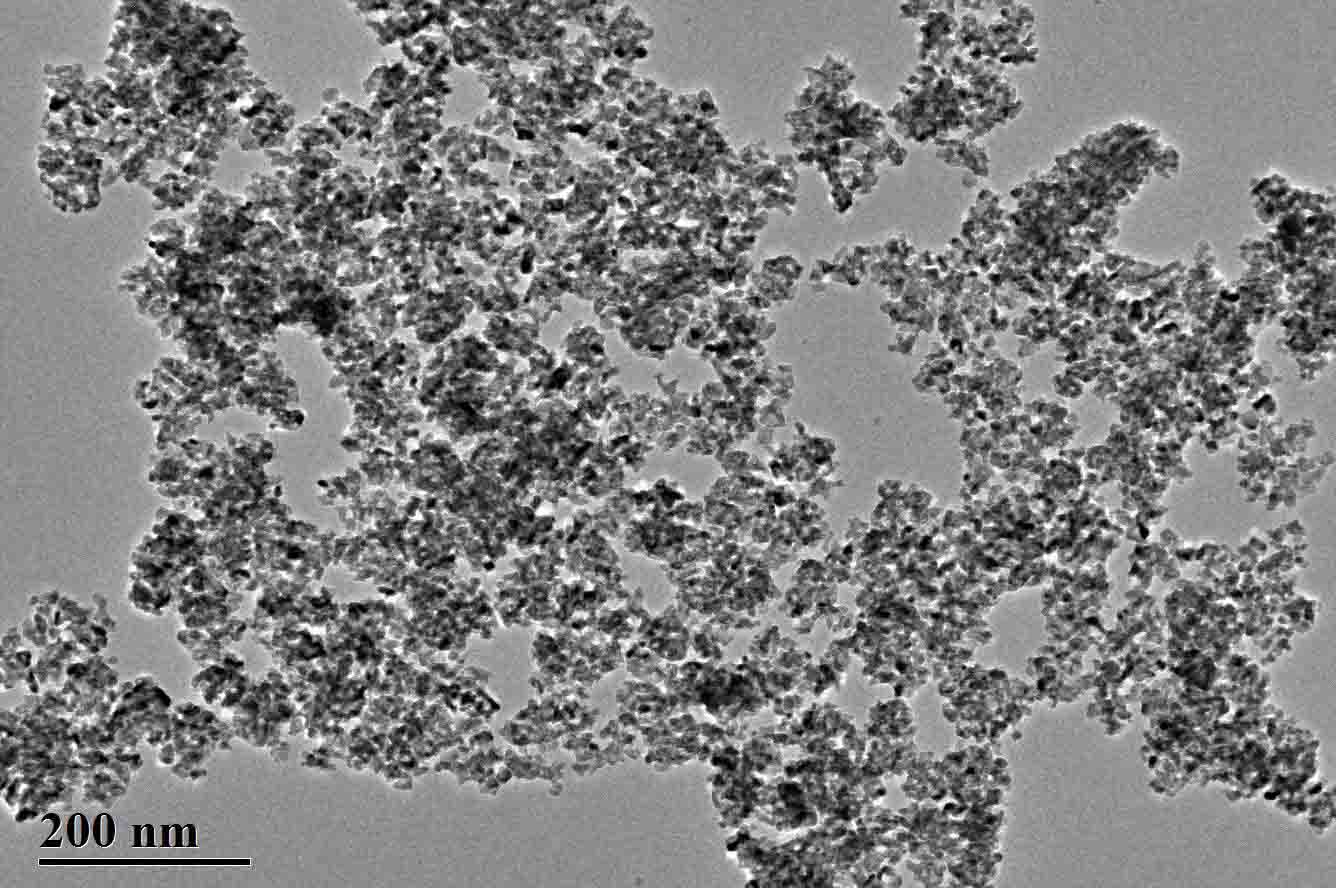

Supplement: Supplementary file 3 — Source Data [file 41467_2023_38336_MOESM3_ESM.zip › Source_Data_for_Figures_in_Supplementary_Information/Source_Data_Supplementary_Figure_02/Supplementary_Figure_2a.jpg]

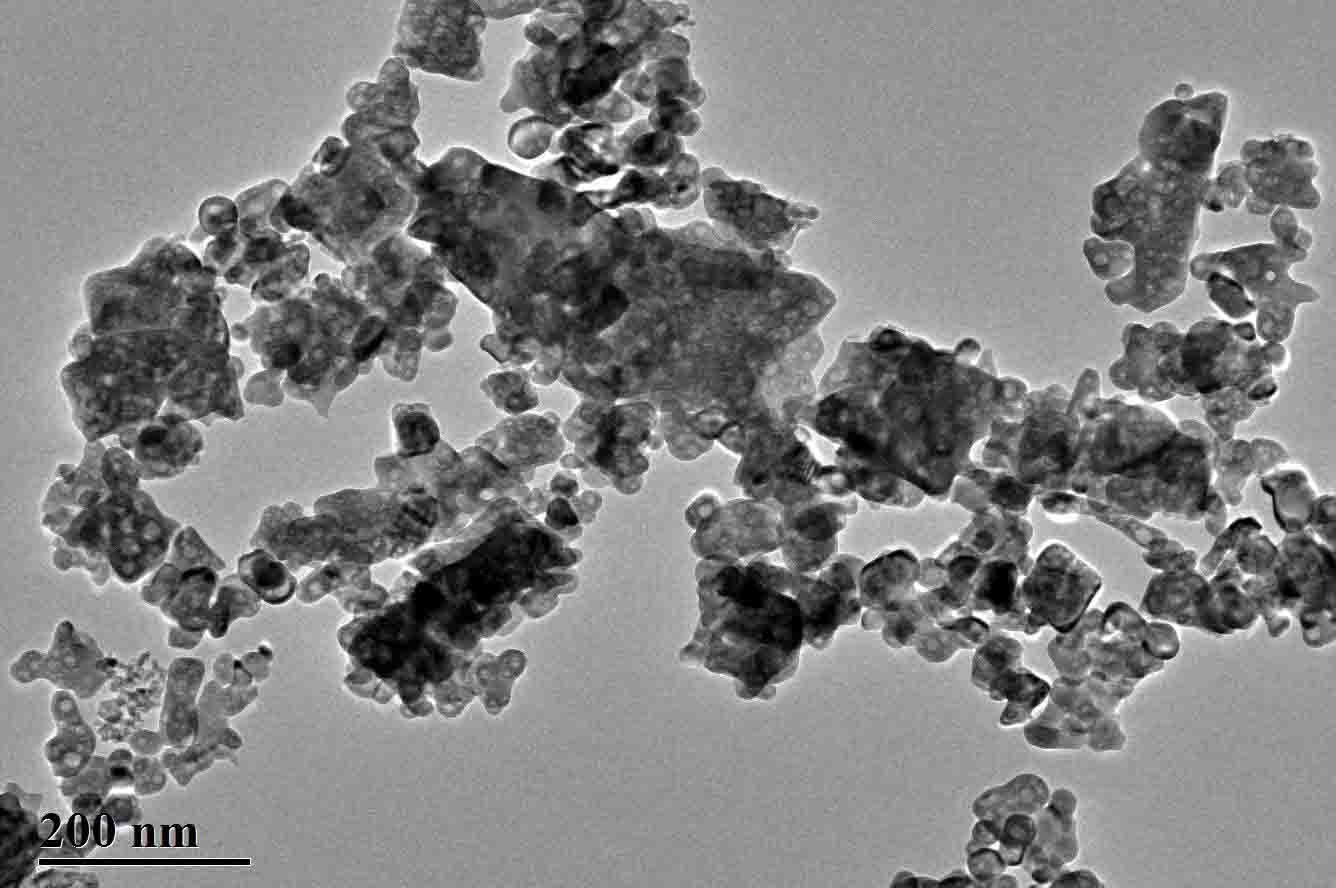

Supplement: Supplementary file 3 — Source Data [file 41467_2023_38336_MOESM3_ESM.zip › Source_Data_for_Figures_in_Supplementary_Information/Source_Data_Supplementary_Figure_02/Supplementary_Figure_2b.jpg]

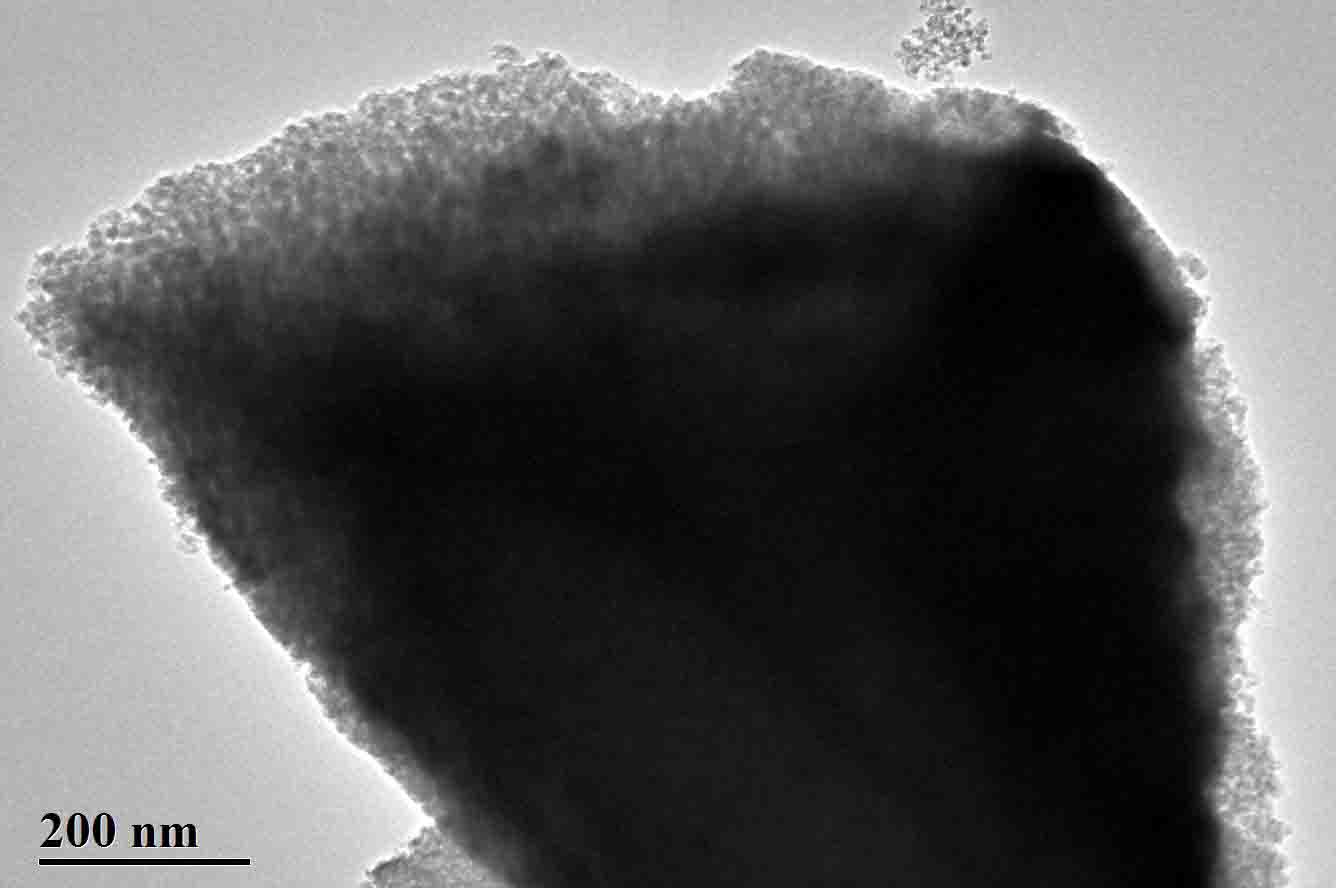

Supplement: Supplementary file 3 — Source Data [file 41467_2023_38336_MOESM3_ESM.zip › Source_Data_for_Figures_in_Supplementary_Information/Source_Data_Supplementary_Figure_02/Supplementary_Figure_2c.jpg]

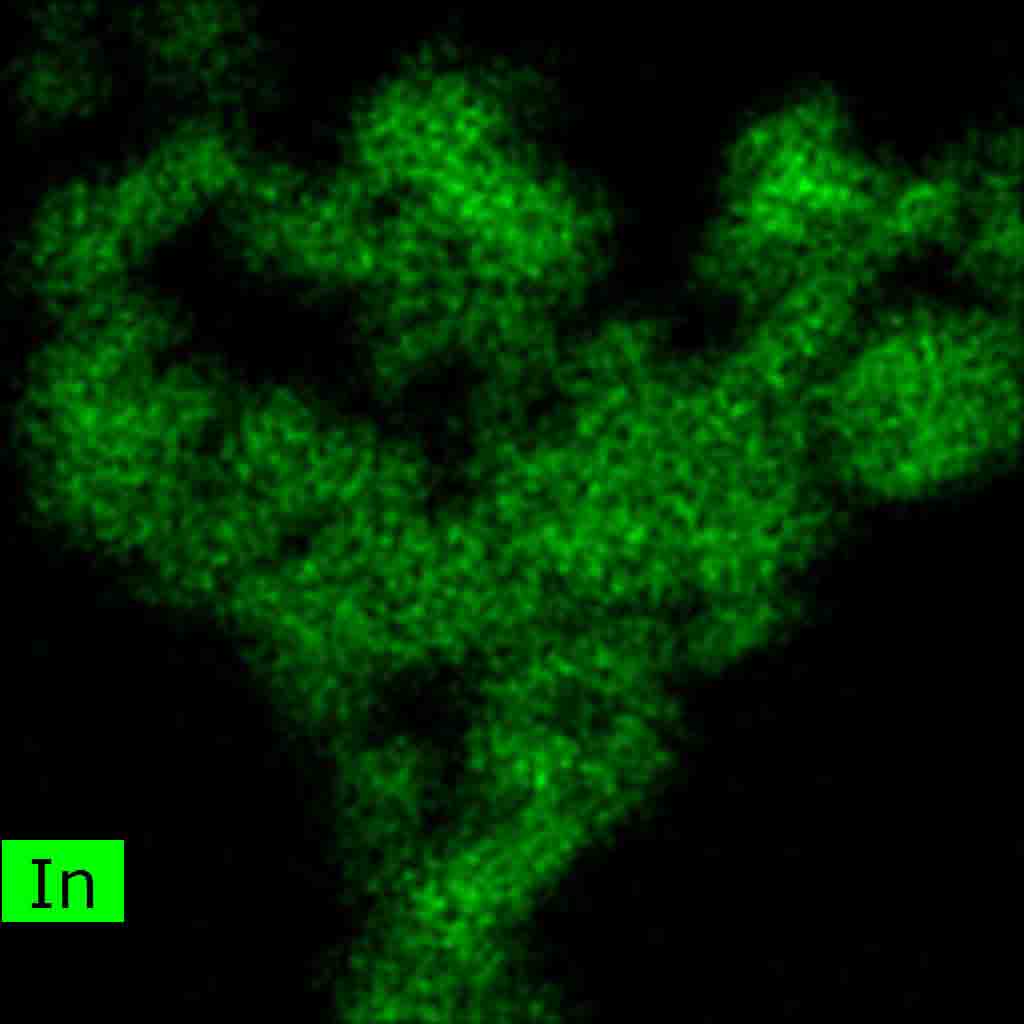

Supplement: Supplementary file 3 — Source Data [file 41467_2023_38336_MOESM3_ESM.zip › Source_Data_for_Figures_in_Supplementary_Information/Source_Data_Supplementary_Figure_03/Supplementary_Figure 3-3.jpg]

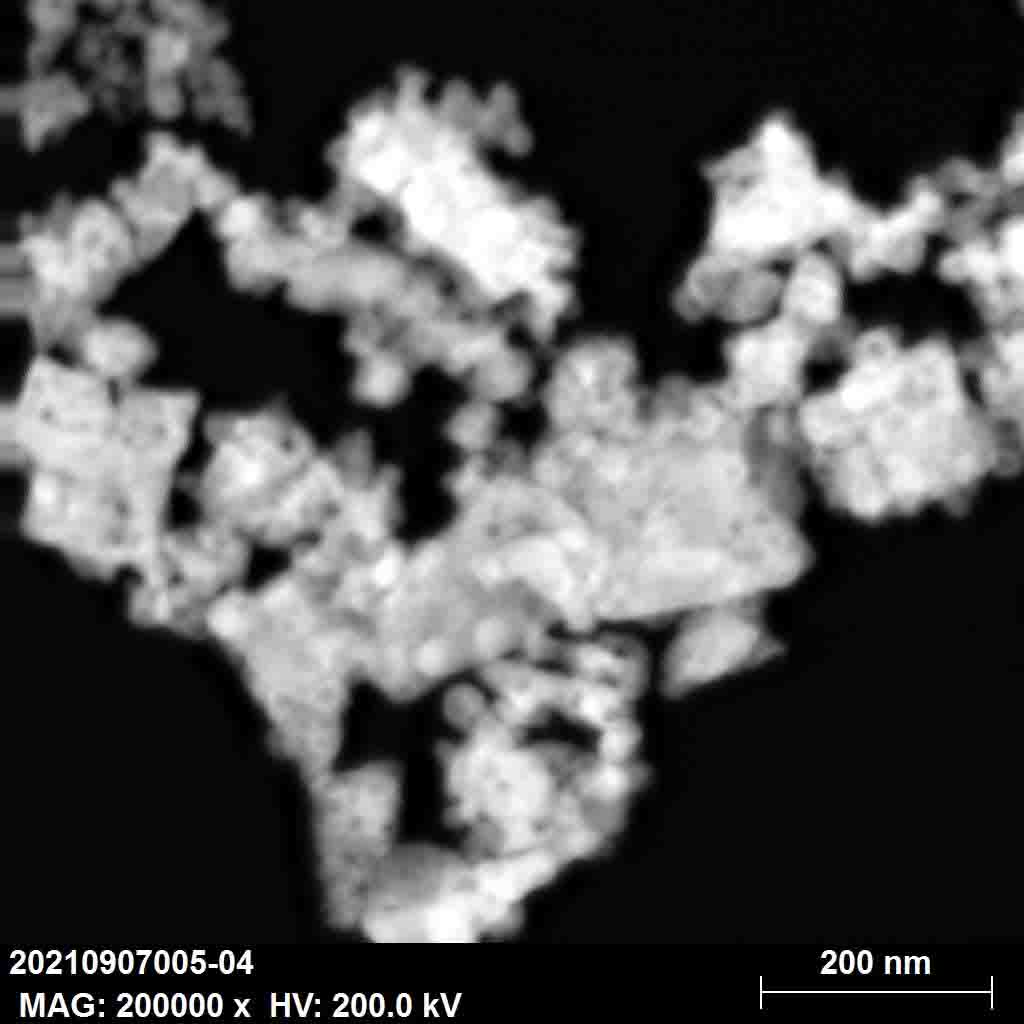

Supplement: Supplementary file 3 — Source Data [file 41467_2023_38336_MOESM3_ESM.zip › Source_Data_for_Figures_in_Supplementary_Information/Source_Data_Supplementary_Figure_03/Supplementary_Figure_3-1.jpg]

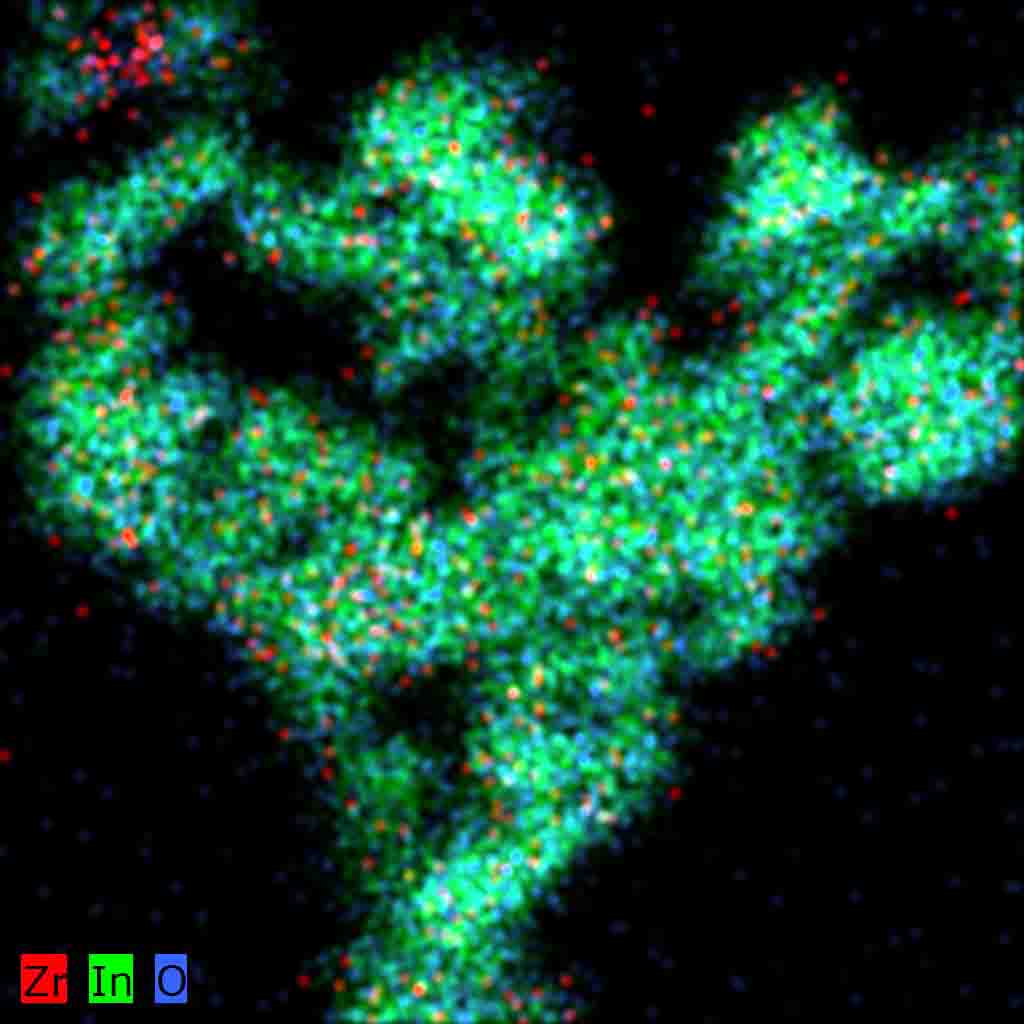

Supplement: Supplementary file 3 — Source Data [file 41467_2023_38336_MOESM3_ESM.zip › Source_Data_for_Figures_in_Supplementary_Information/Source_Data_Supplementary_Figure_03/Supplementary_Figure_3-2.jpg]

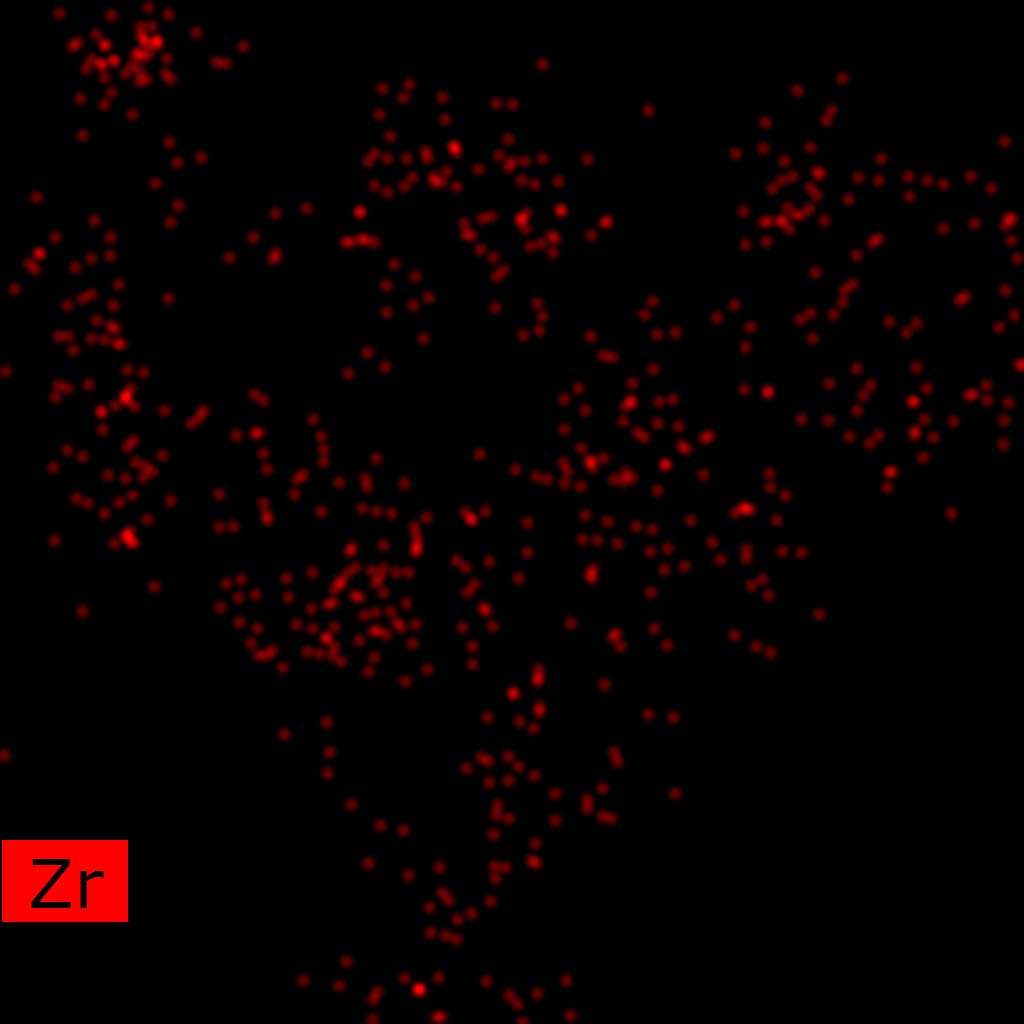

Supplement: Supplementary file 3 — Source Data [file 41467_2023_38336_MOESM3_ESM.zip › Source_Data_for_Figures_in_Supplementary_Information/Source_Data_Supplementary_Figure_03/Supplementary_Figure_3-4.jpg]

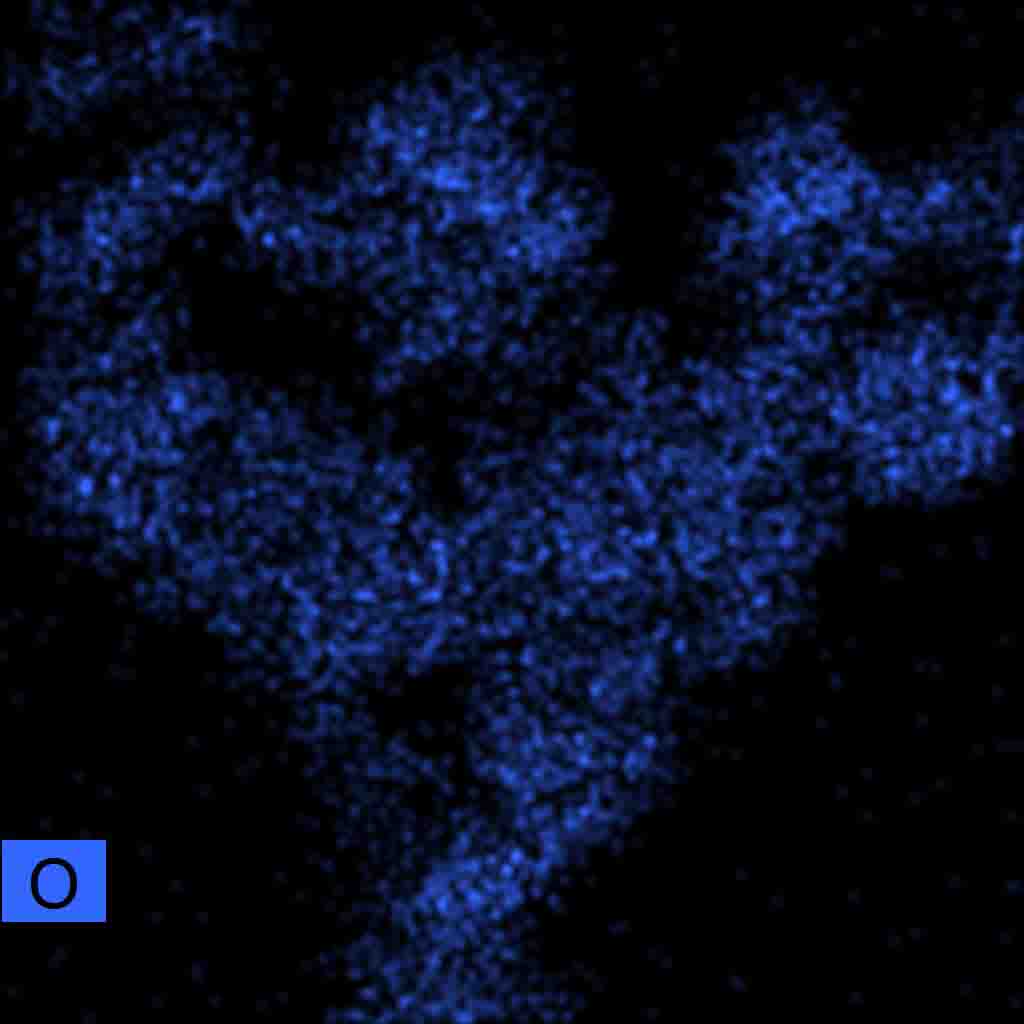

Supplement: Supplementary file 3 — Source Data [file 41467_2023_38336_MOESM3_ESM.zip › Source_Data_for_Figures_in_Supplementary_Information/Source_Data_Supplementary_Figure_03/Supplementary_Figure_3-5.jpg]

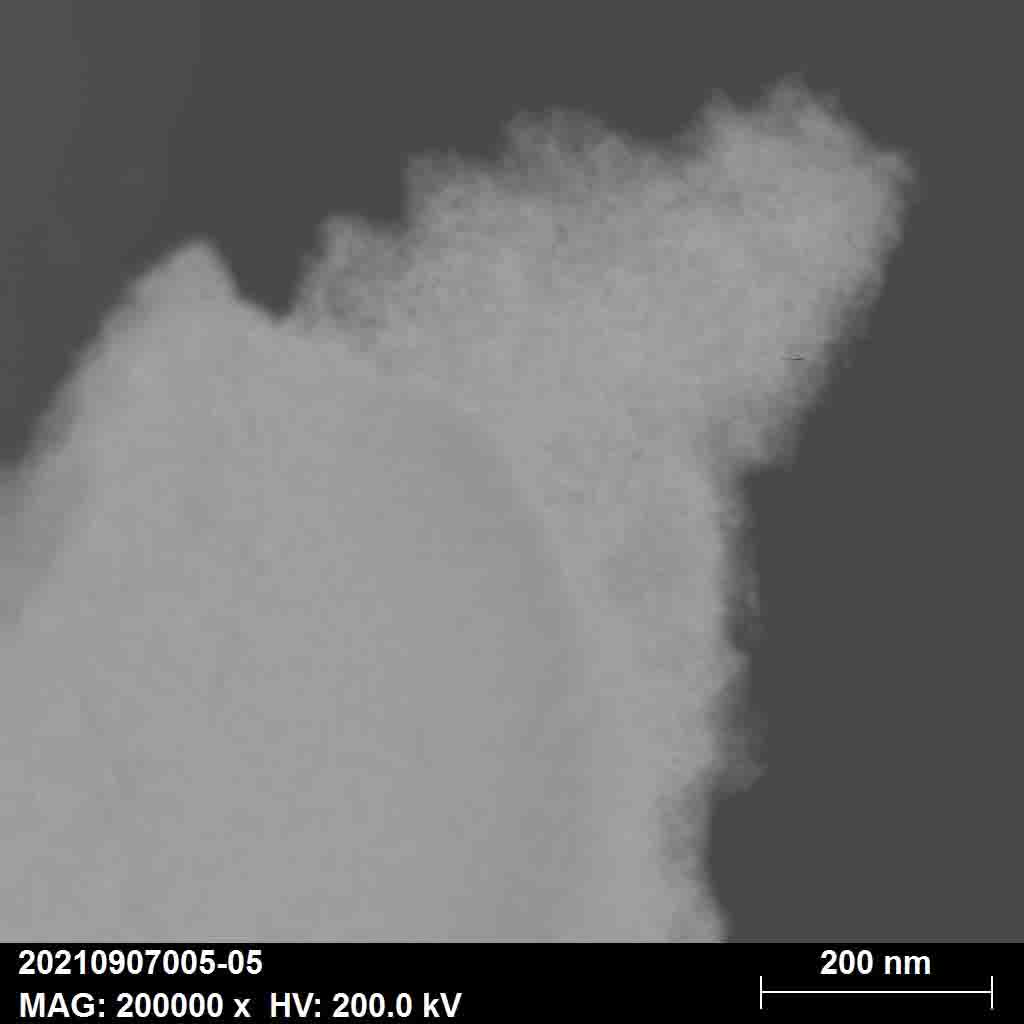

Supplement: Supplementary file 3 — Source Data [file 41467_2023_38336_MOESM3_ESM.zip › Source_Data_for_Figures_in_Supplementary_Information/Source_Data_Supplementary_Figure_04/Supplementary_Figure 4-1.jpg]

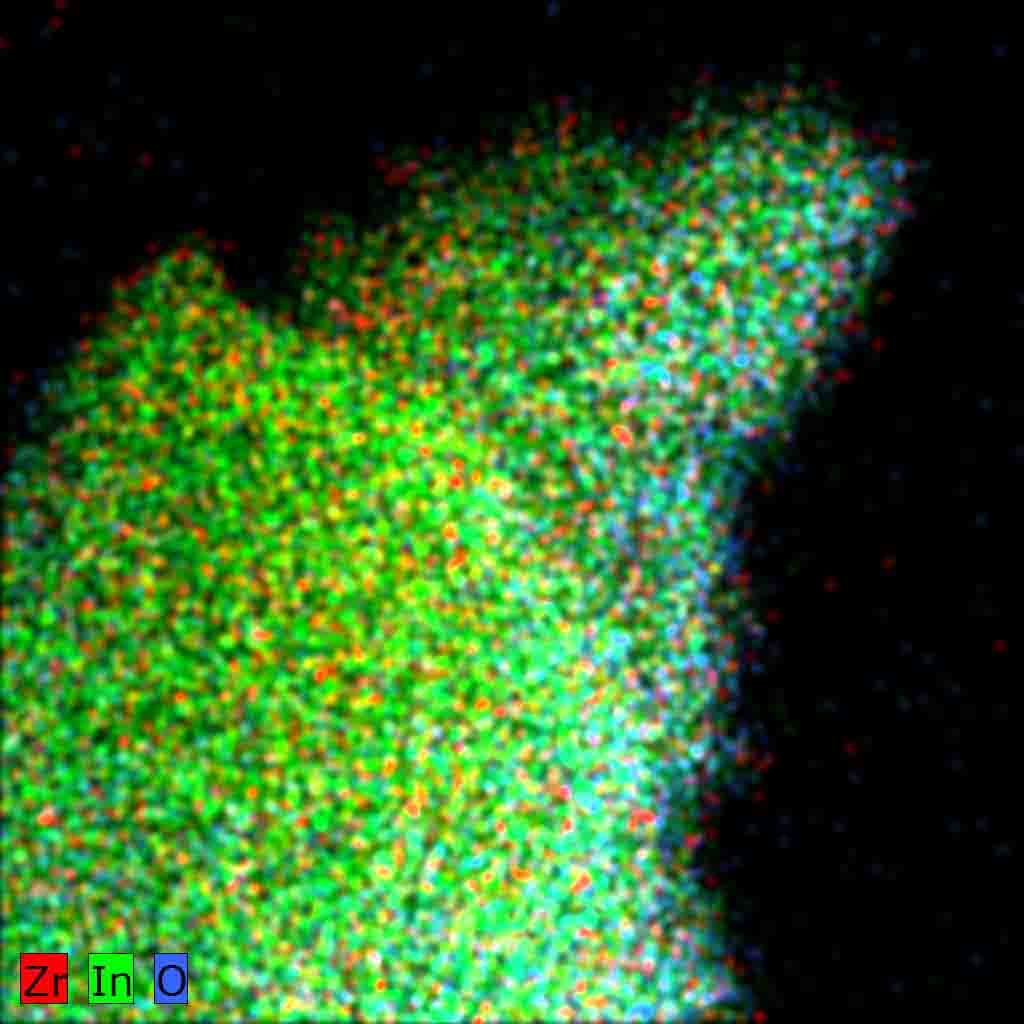

Supplement: Supplementary file 3 — Source Data [file 41467_2023_38336_MOESM3_ESM.zip › Source_Data_for_Figures_in_Supplementary_Information/Source_Data_Supplementary_Figure_04/Supplementary_Figure 4-2.jpg]

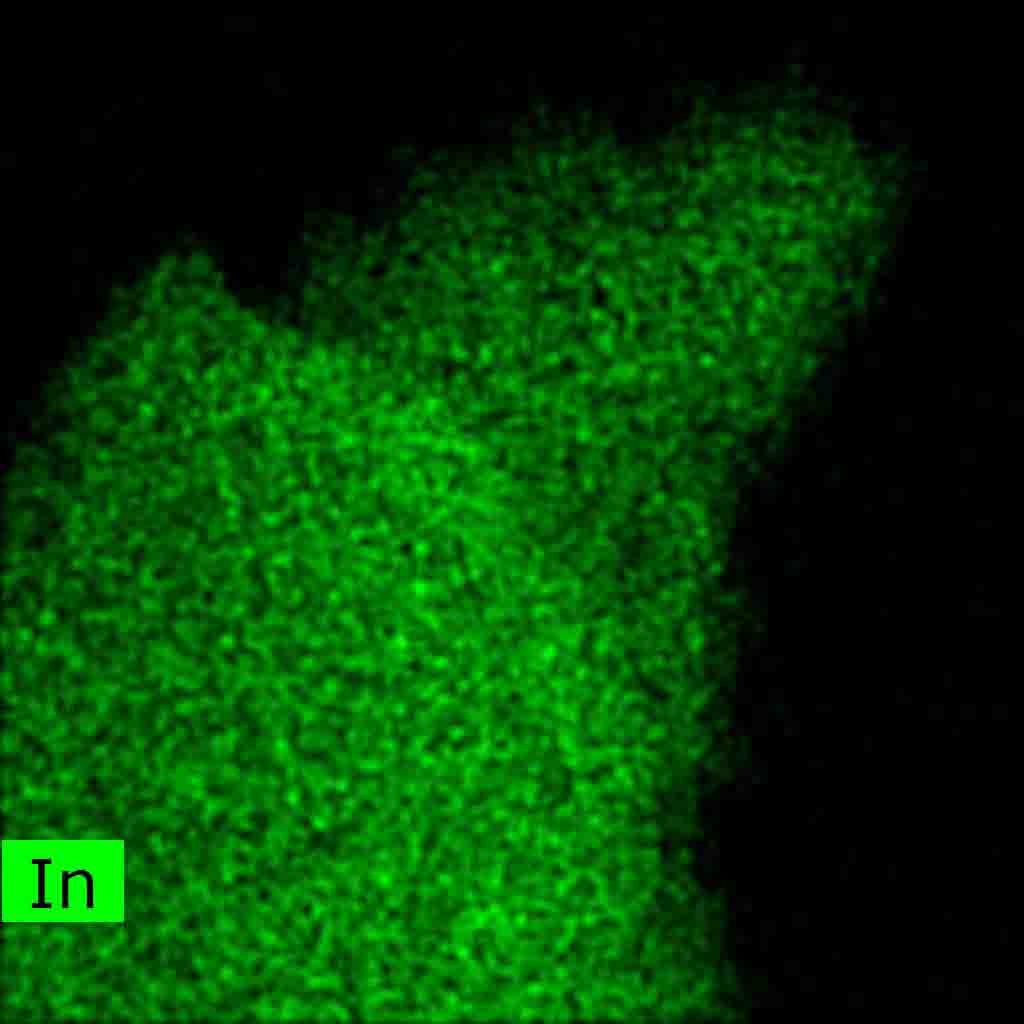

Supplement: Supplementary file 3 — Source Data [file 41467_2023_38336_MOESM3_ESM.zip › Source_Data_for_Figures_in_Supplementary_Information/Source_Data_Supplementary_Figure_04/Supplementary_Figure 4-3.jpg]

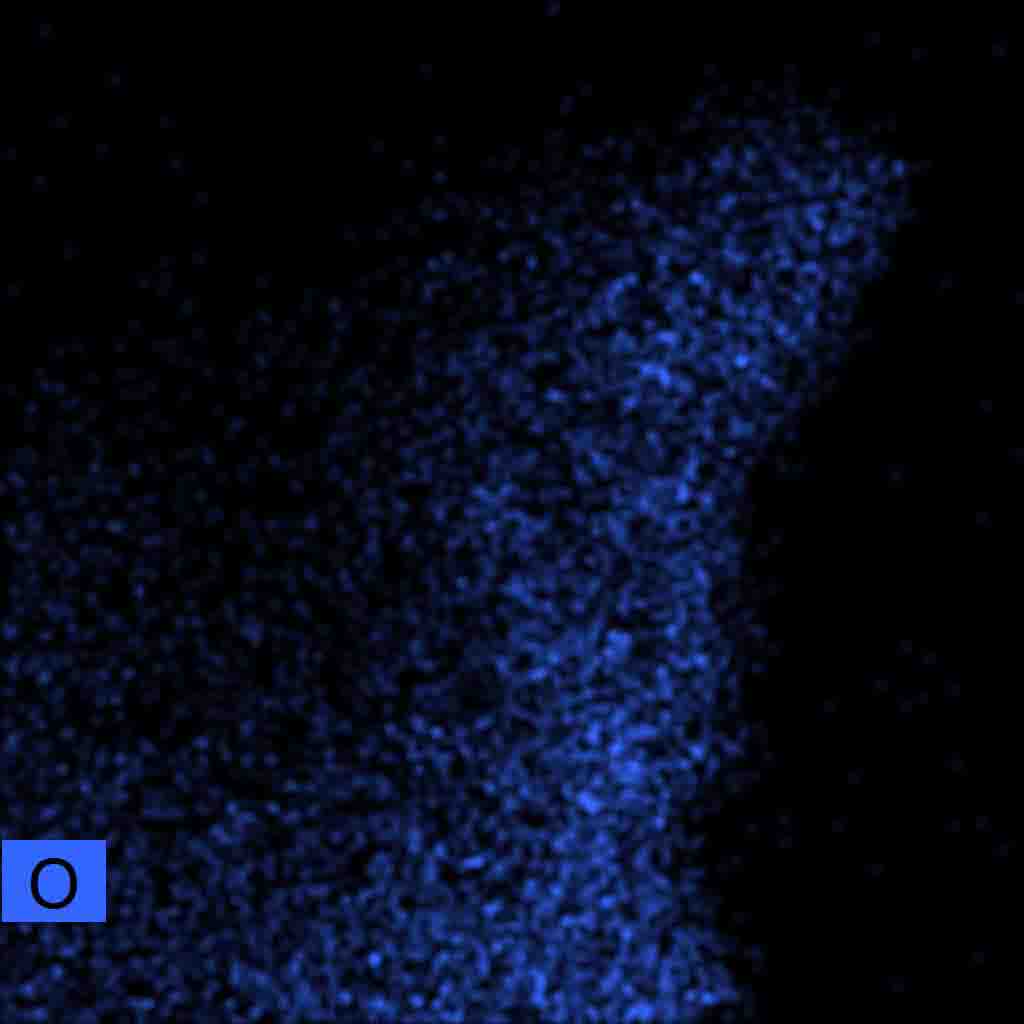

Supplement: Supplementary file 3 — Source Data [file 41467_2023_38336_MOESM3_ESM.zip › Source_Data_for_Figures_in_Supplementary_Information/Source_Data_Supplementary_Figure_04/Supplementary_Figure 4-4.jpg]

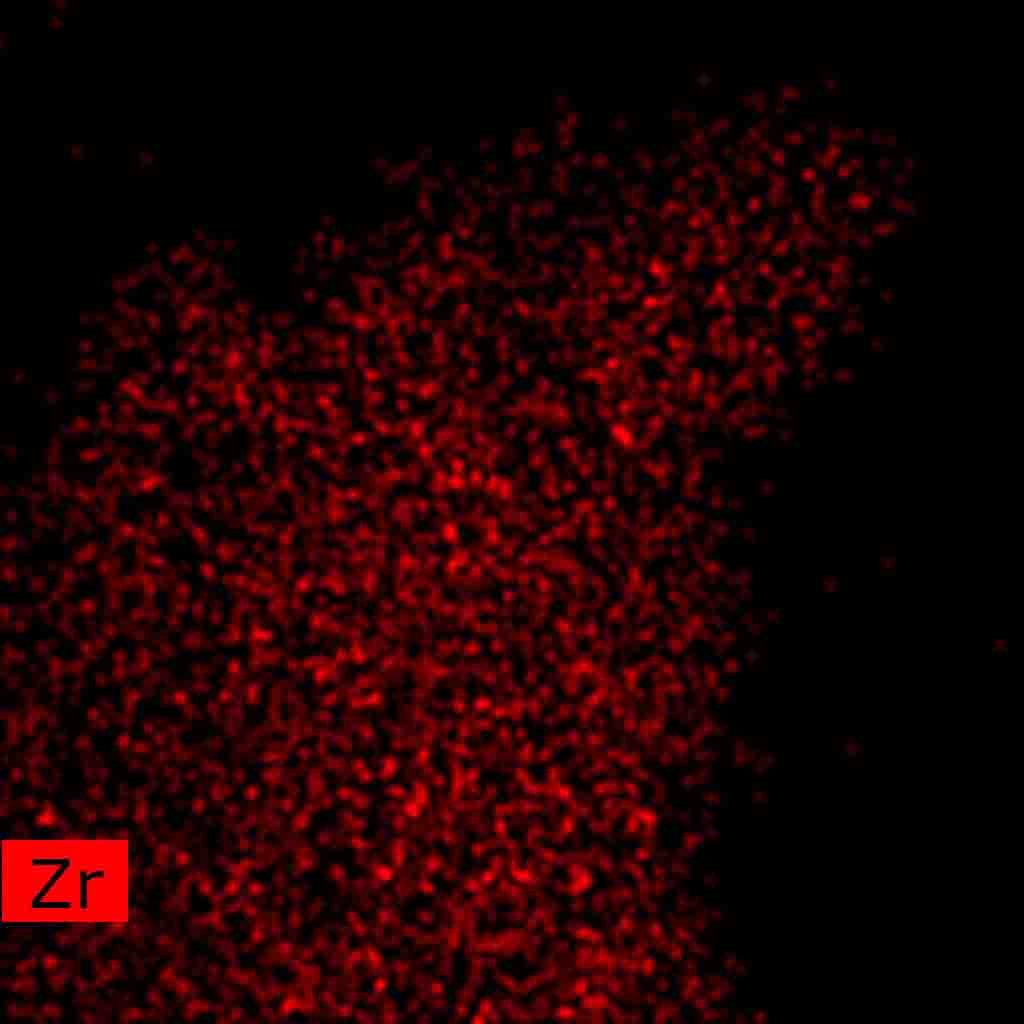

Supplement: Supplementary file 3 — Source Data [file 41467_2023_38336_MOESM3_ESM.zip › Source_Data_for_Figures_in_Supplementary_Information/Source_Data_Supplementary_Figure_04/Supplementary_Figure 4-5.jpg]

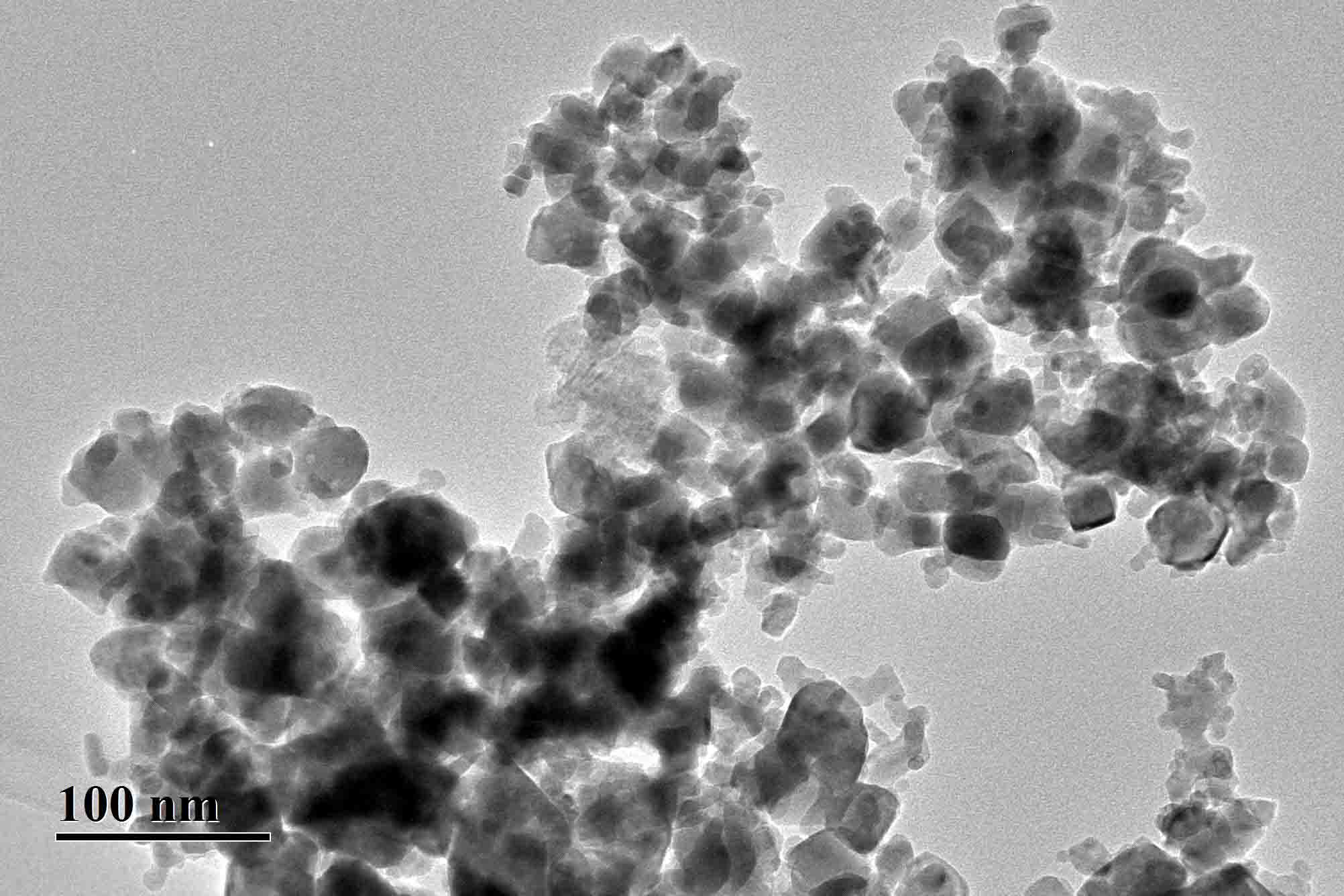

Supplement: Supplementary file 3 — Source Data [file 41467_2023_38336_MOESM3_ESM.zip › Source_Data_for_Figures_in_Supplementary_Information/Source_Data_Supplementary_Figure_12/Supplementary_Figure_12b.jpg]

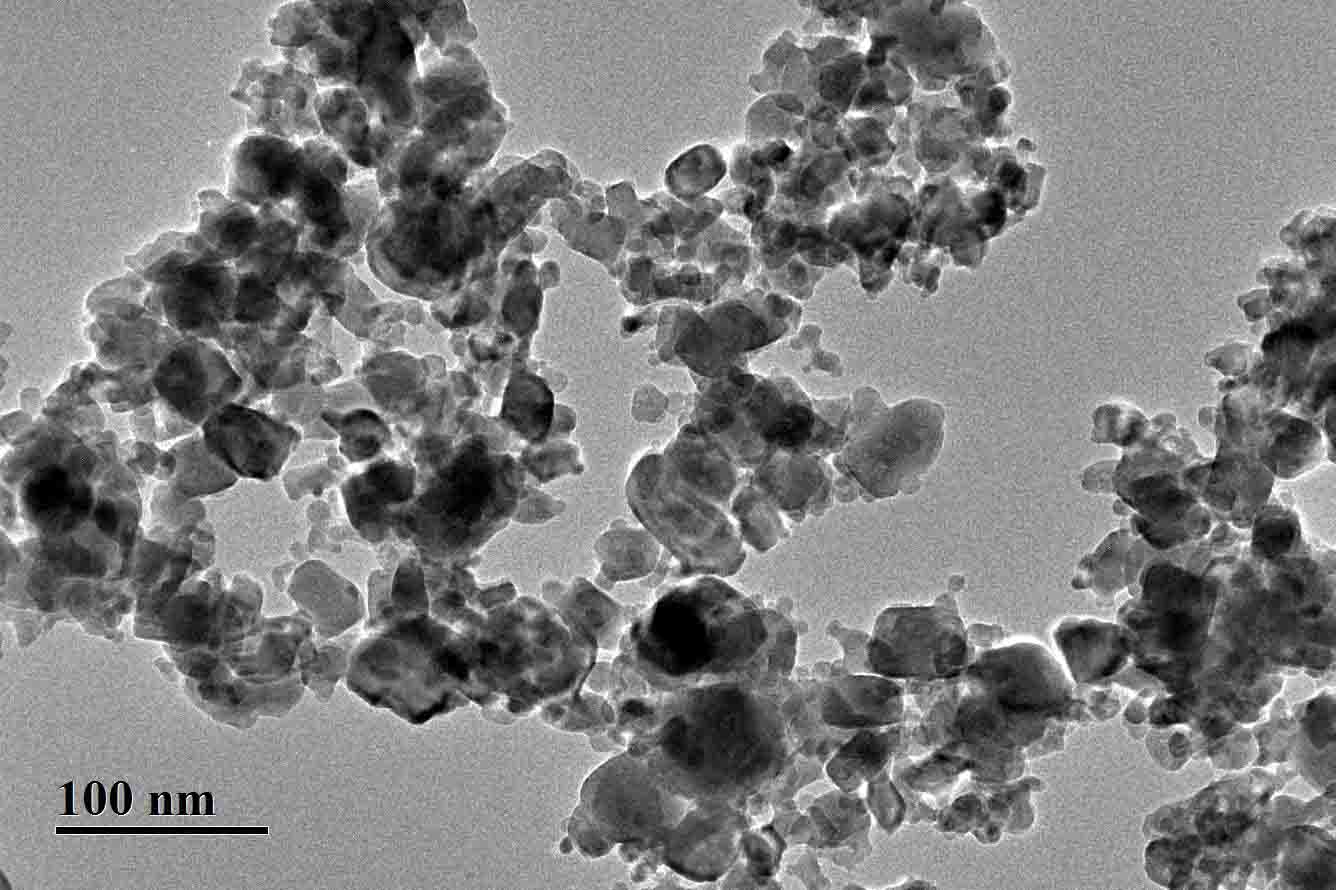

Supplement: Supplementary file 3 — Source Data [file 41467_2023_38336_MOESM3_ESM.zip › Source_Data_for_Figures_in_Supplementary_Information/Source_Data_Supplementary_Figure_12/Supplementary_Figure_12c.jpg]

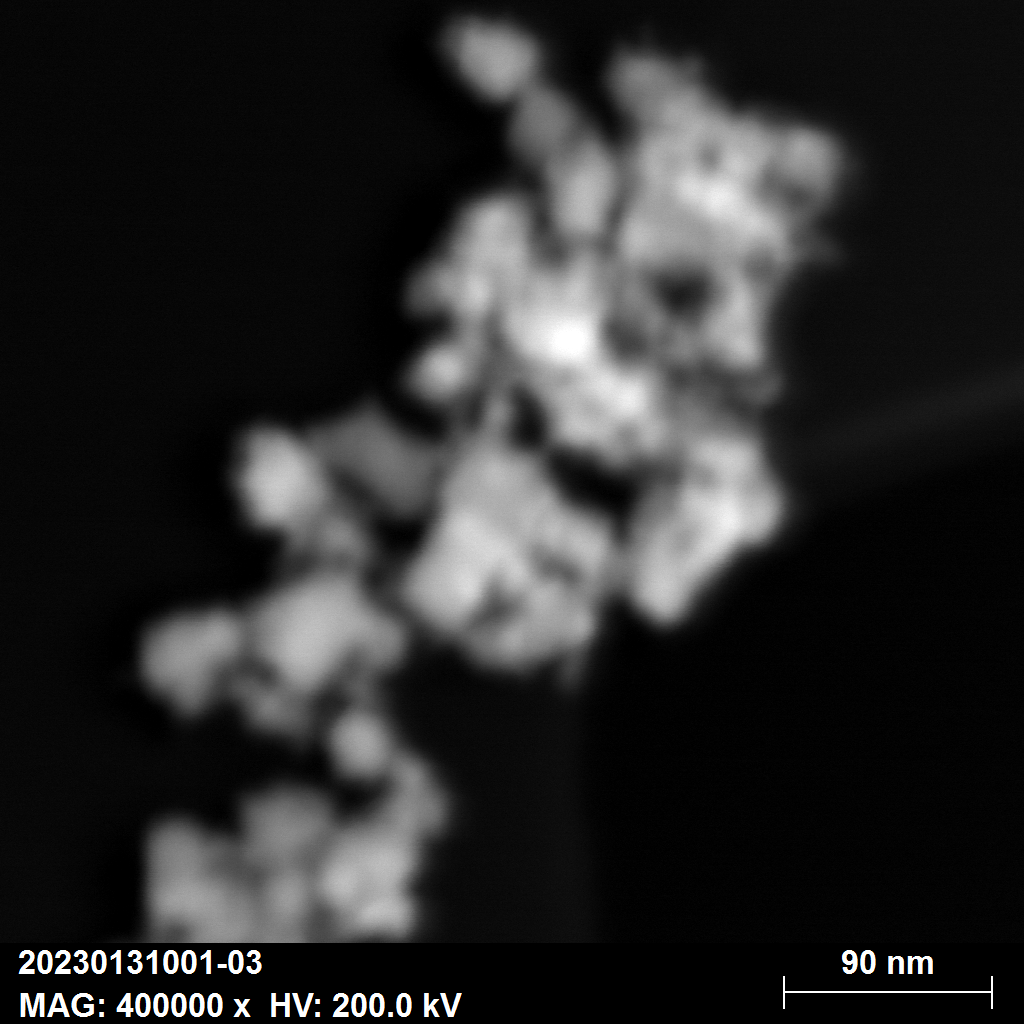

Supplement: Supplementary file 3 — Source Data [file 41467_2023_38336_MOESM3_ESM.zip › Source_Data_for_Figures_in_Supplementary_Information/Source_Data_Supplementary_Figure_14/Supplementary_Figure_14a-1.bmp]

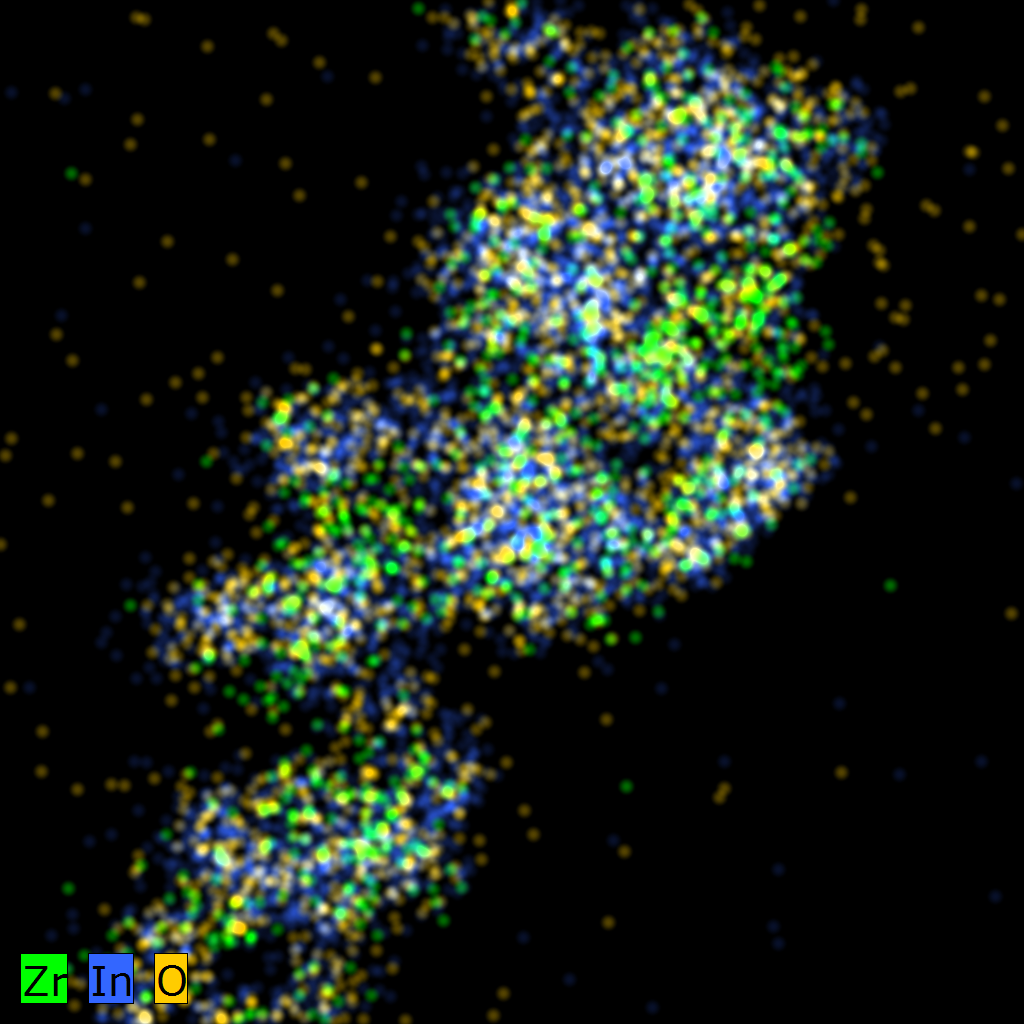

Supplement: Supplementary file 3 — Source Data [file 41467_2023_38336_MOESM3_ESM.zip › Source_Data_for_Figures_in_Supplementary_Information/Source_Data_Supplementary_Figure_14/Supplementary_Figure_14a-2.bmp]

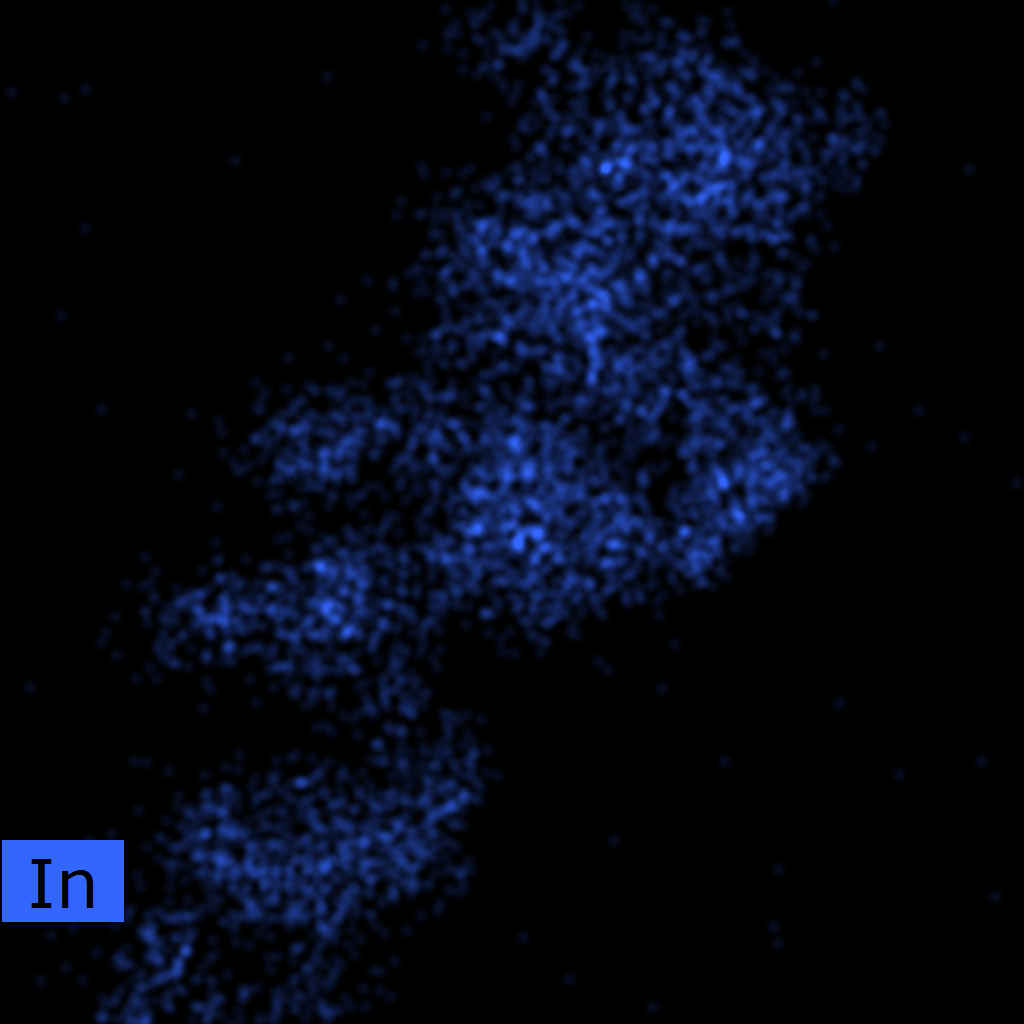

Supplement: Supplementary file 3 — Source Data [file 41467_2023_38336_MOESM3_ESM.zip › Source_Data_for_Figures_in_Supplementary_Information/Source_Data_Supplementary_Figure_14/Supplementary_Figure_14a-3.bmp]

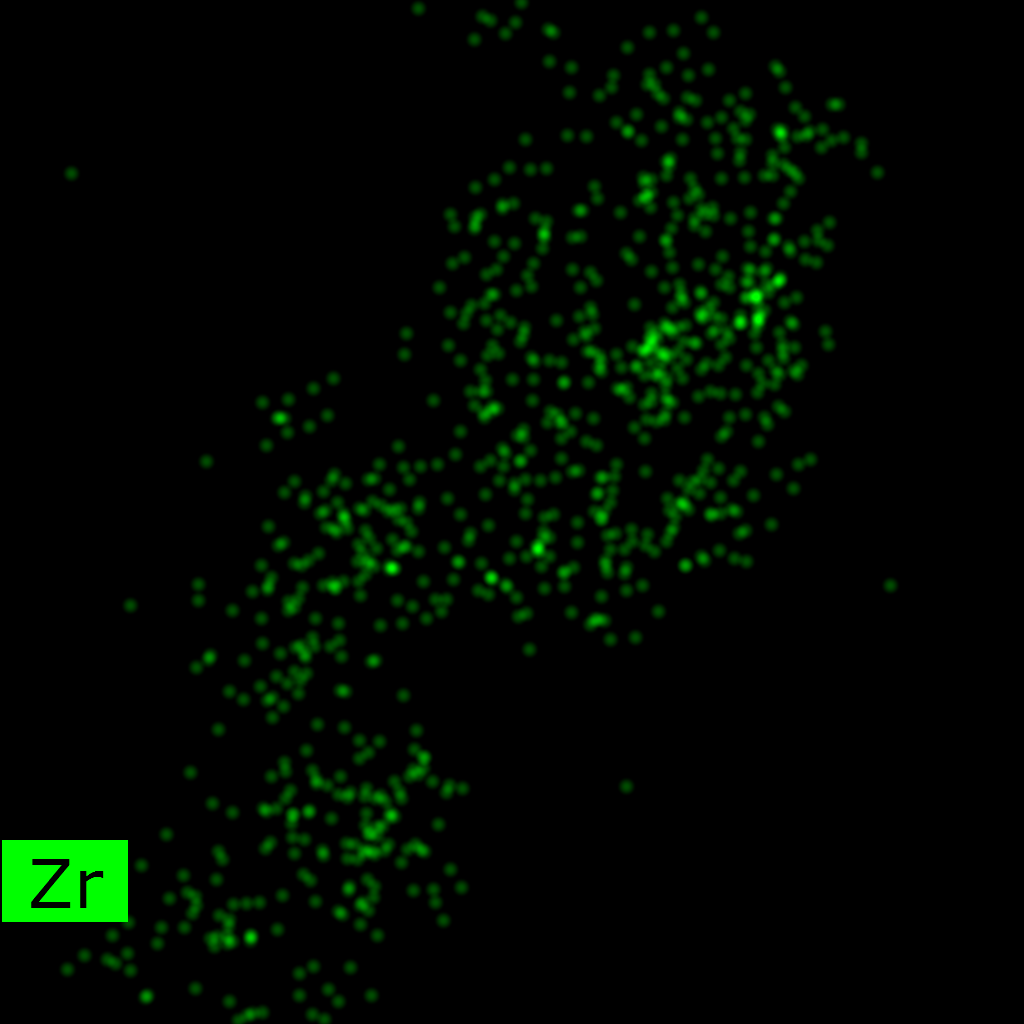

Supplement: Supplementary file 3 — Source Data [file 41467_2023_38336_MOESM3_ESM.zip › Source_Data_for_Figures_in_Supplementary_Information/Source_Data_Supplementary_Figure_14/Supplementary_Figure_14a-4.bmp]

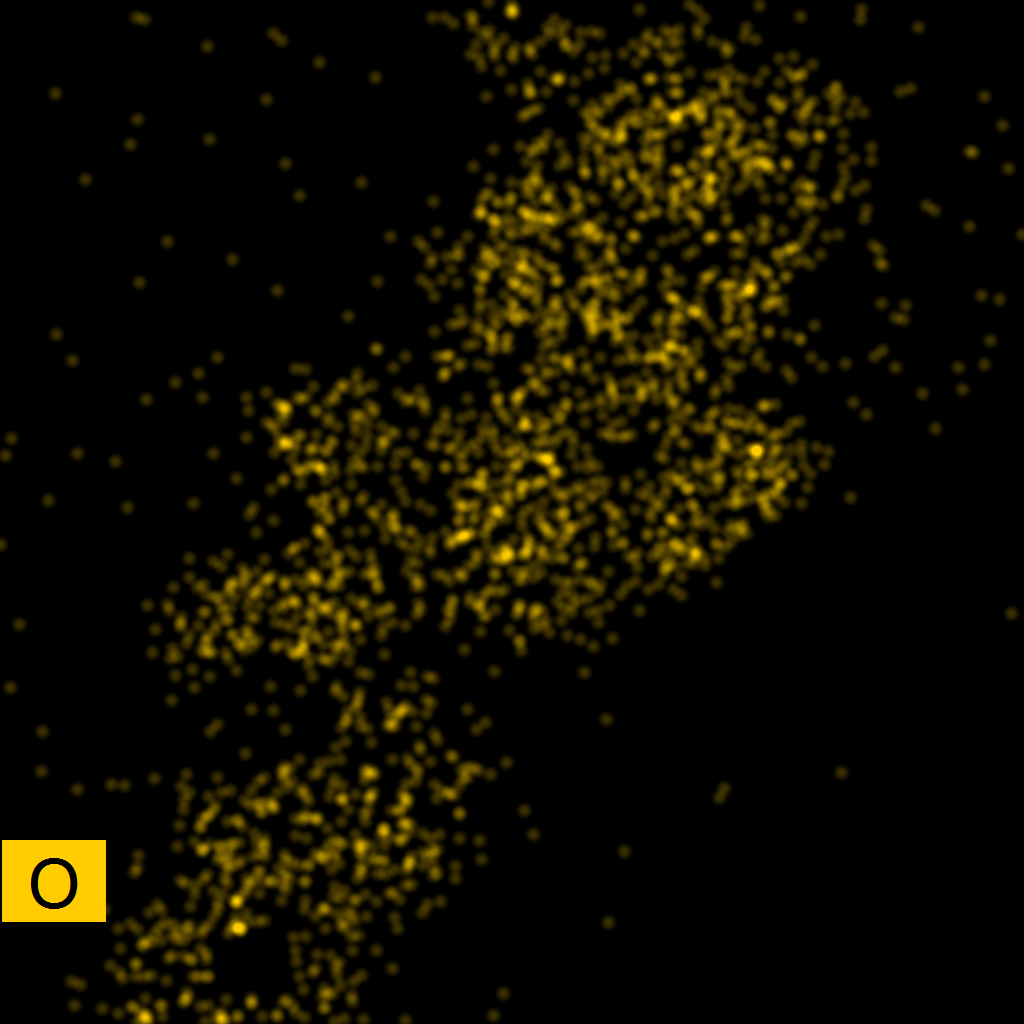

Supplement: Supplementary file 3 — Source Data [file 41467_2023_38336_MOESM3_ESM.zip › Source_Data_for_Figures_in_Supplementary_Information/Source_Data_Supplementary_Figure_14/Supplementary_Figure_14a-5.bmp]

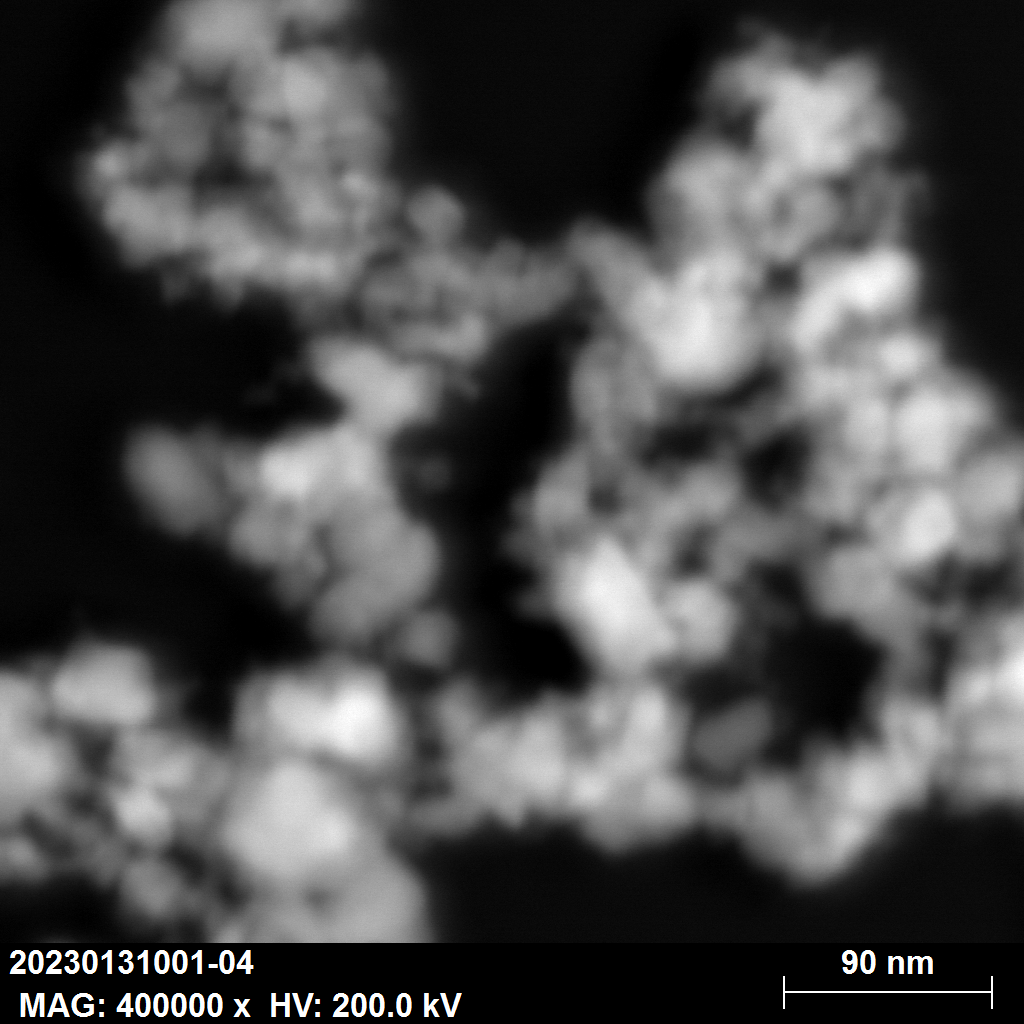

Supplement: Supplementary file 3 — Source Data [file 41467_2023_38336_MOESM3_ESM.zip › Source_Data_for_Figures_in_Supplementary_Information/Source_Data_Supplementary_Figure_14/Supplementary_Figure_14b-1.bmp]

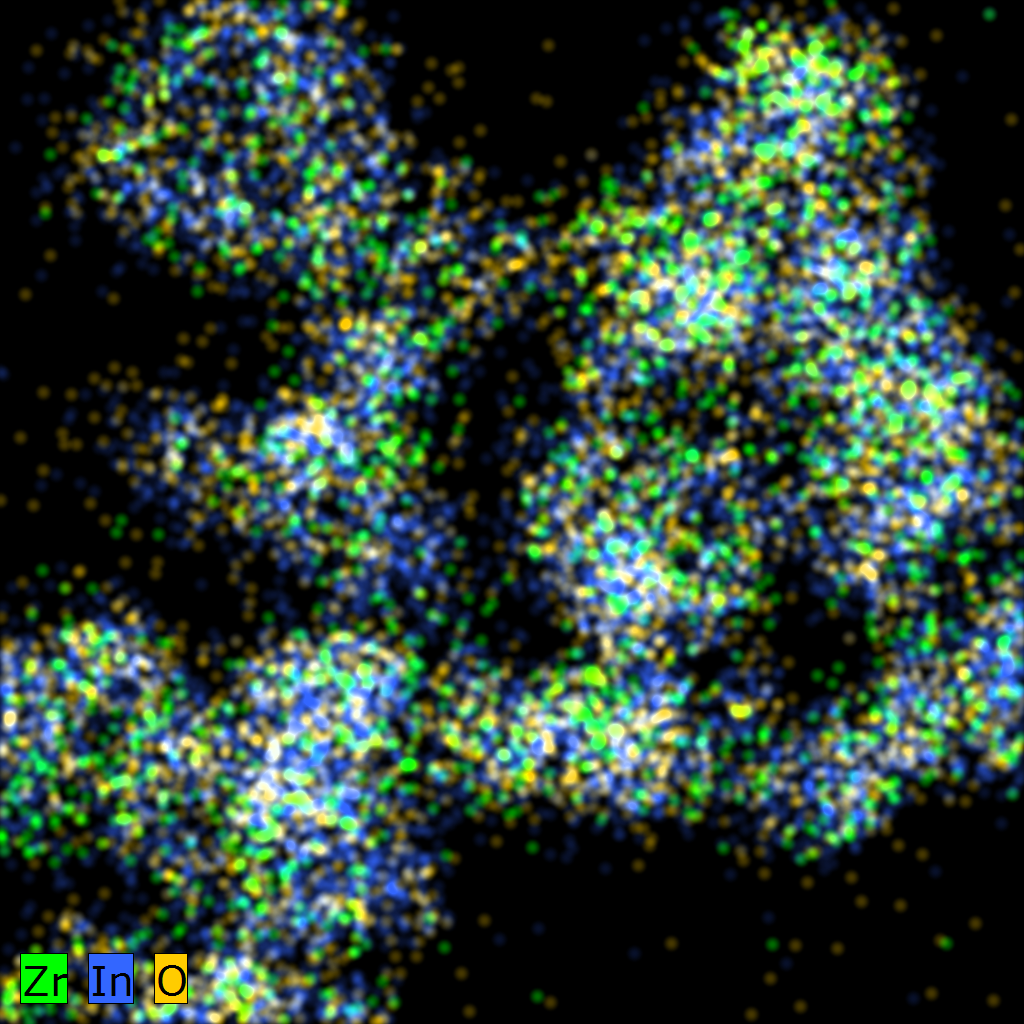

Supplement: Supplementary file 3 — Source Data [file 41467_2023_38336_MOESM3_ESM.zip › Source_Data_for_Figures_in_Supplementary_Information/Source_Data_Supplementary_Figure_14/Supplementary_Figure_14b-2.bmp]

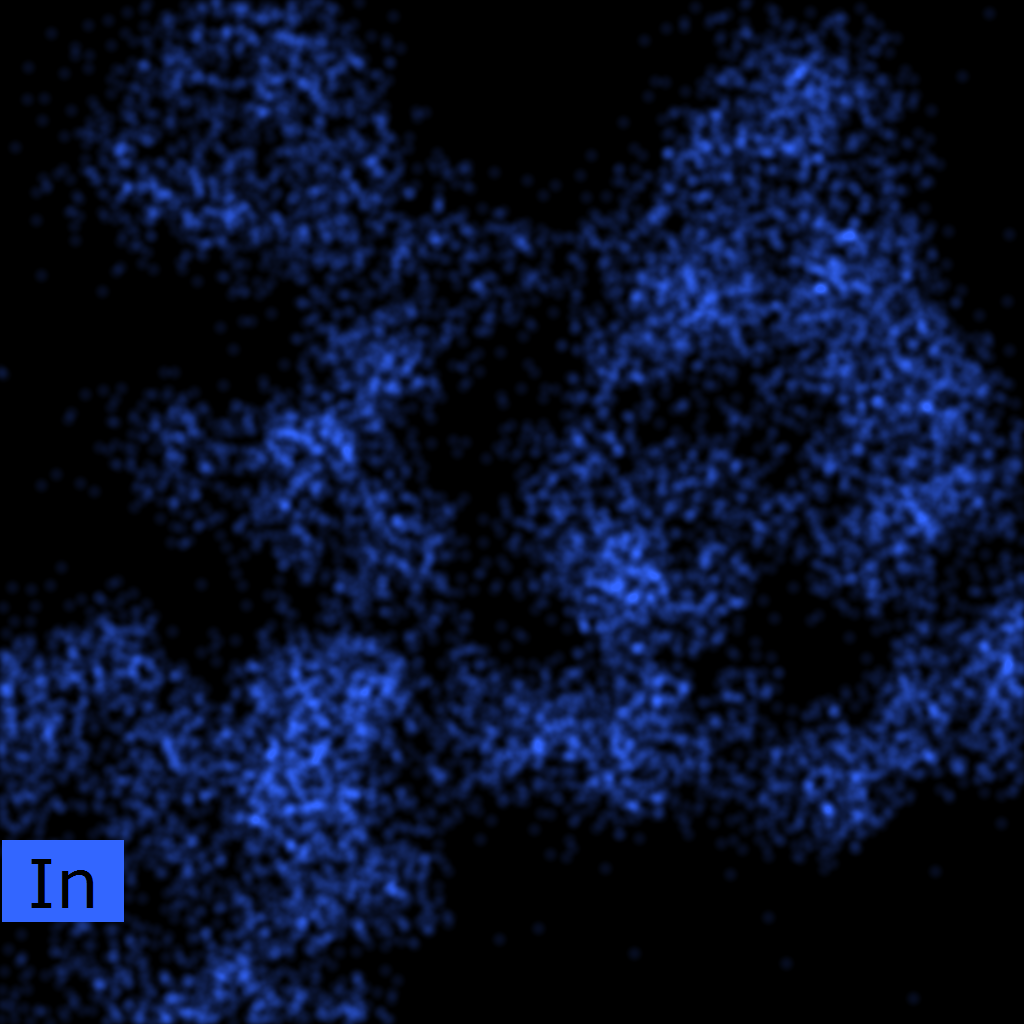

Supplement: Supplementary file 3 — Source Data [file 41467_2023_38336_MOESM3_ESM.zip › Source_Data_for_Figures_in_Supplementary_Information/Source_Data_Supplementary_Figure_14/Supplementary_Figure_14b-3.bmp]

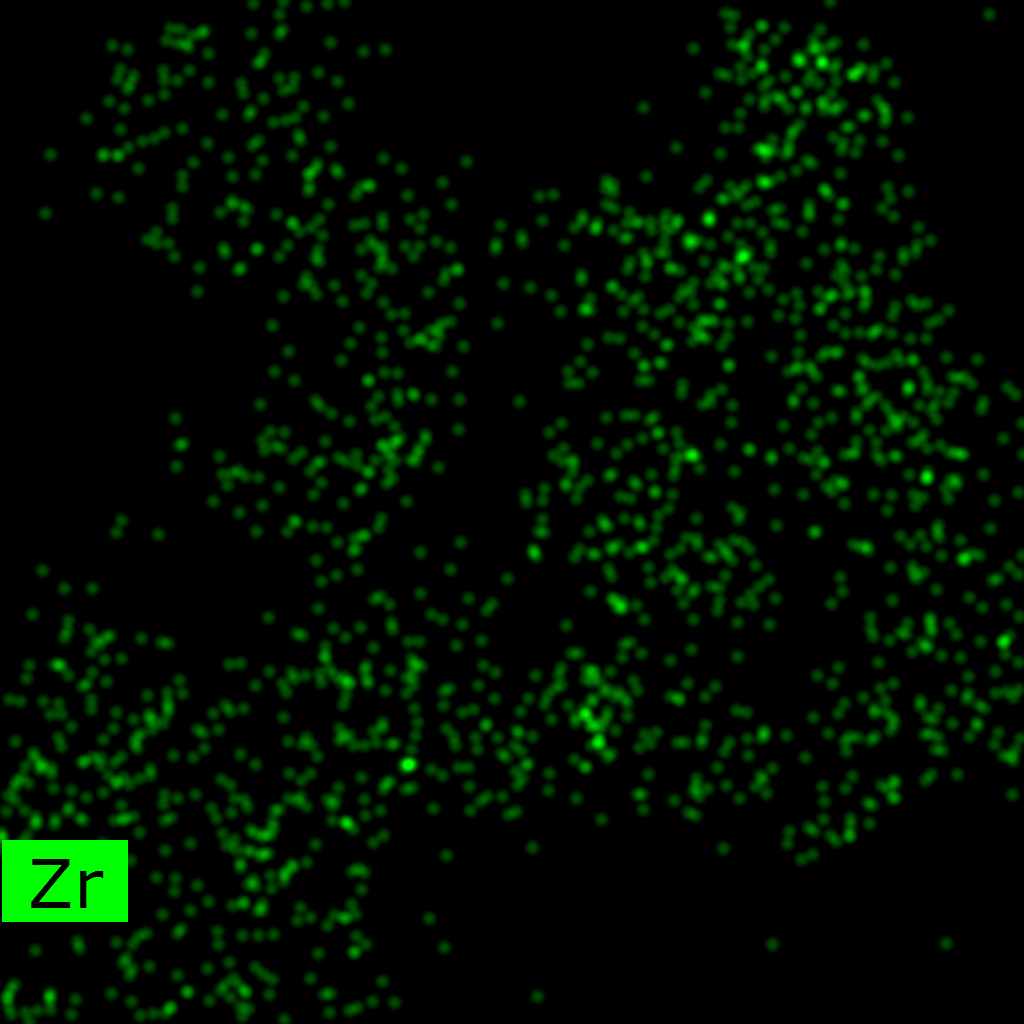

Supplement: Supplementary file 3 — Source Data [file 41467_2023_38336_MOESM3_ESM.zip › Source_Data_for_Figures_in_Supplementary_Information/Source_Data_Supplementary_Figure_14/Supplementary_Figure_14b-4.bmp]

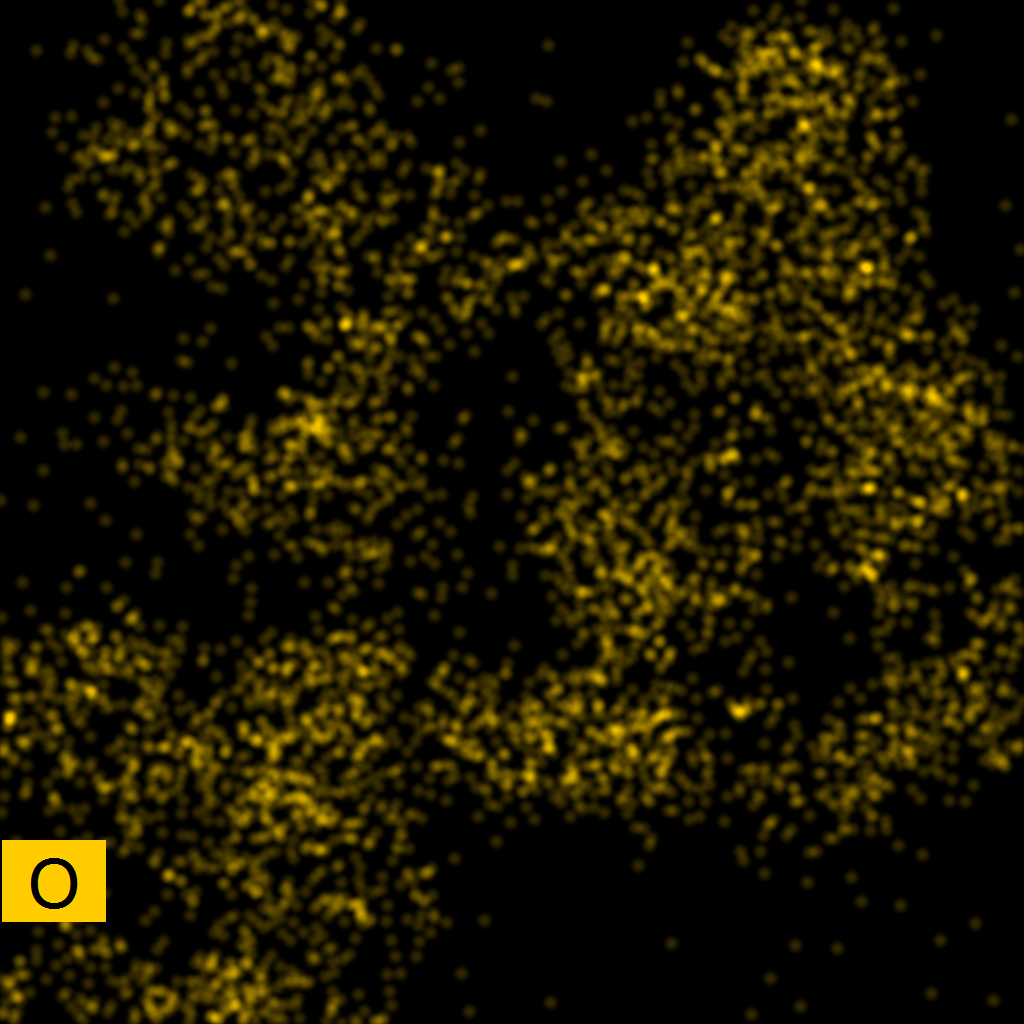

Supplement: Supplementary file 3 — Source Data [file 41467_2023_38336_MOESM3_ESM.zip › Source_Data_for_Figures_in_Supplementary_Information/Source_Data_Supplementary_Figure_14/Supplementary_Figure_14b-5.bmp]

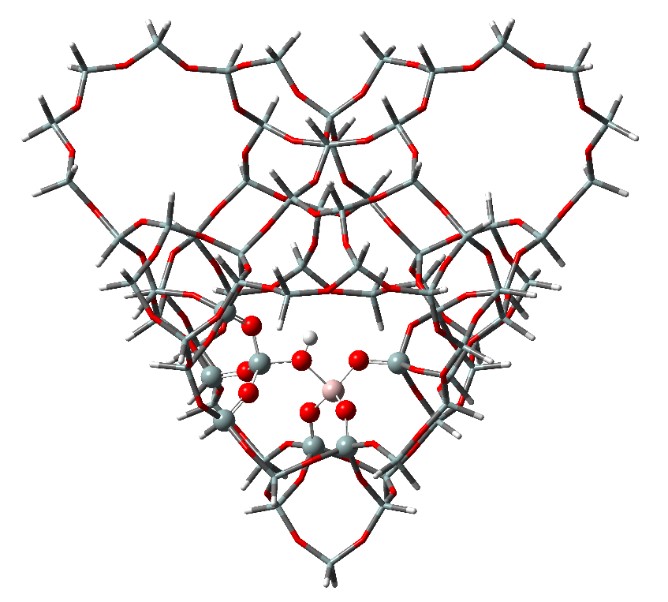

Supplement: Supplementary file 3 — Source Data [file 41467_2023_38336_MOESM3_ESM.zip › Source_Data_for_Figures_in_Supplementary_Information/Source_Data_Supplementary_Figure_20/Supplementary_Figure_20.jpg]

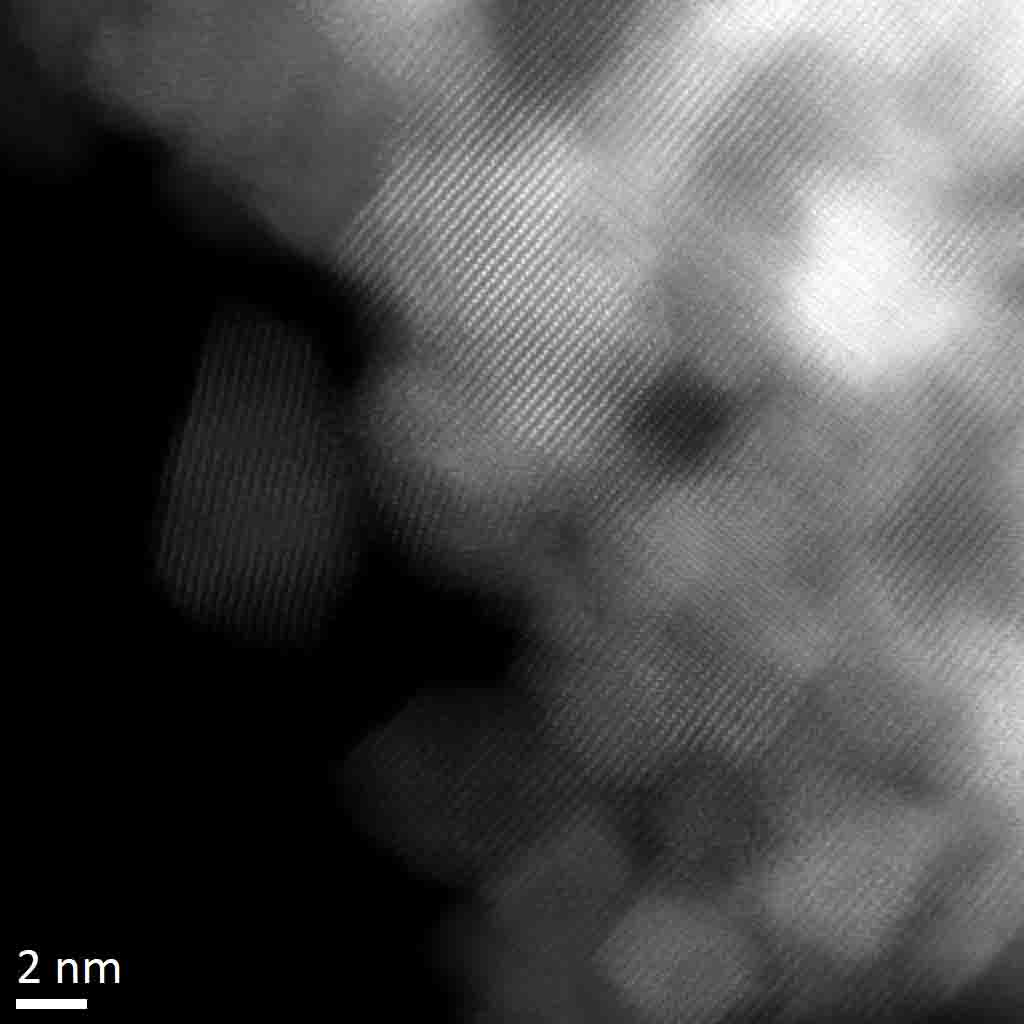

Supplement: Supplementary file 3 — Source Data [file 41467_2023_38336_MOESM3_ESM.zip › Source_Data_for_Figures_in_Supplementary_Information/Source_Data_Supplementary_Figure_22/Supplementary_Figure_22a.jpg]

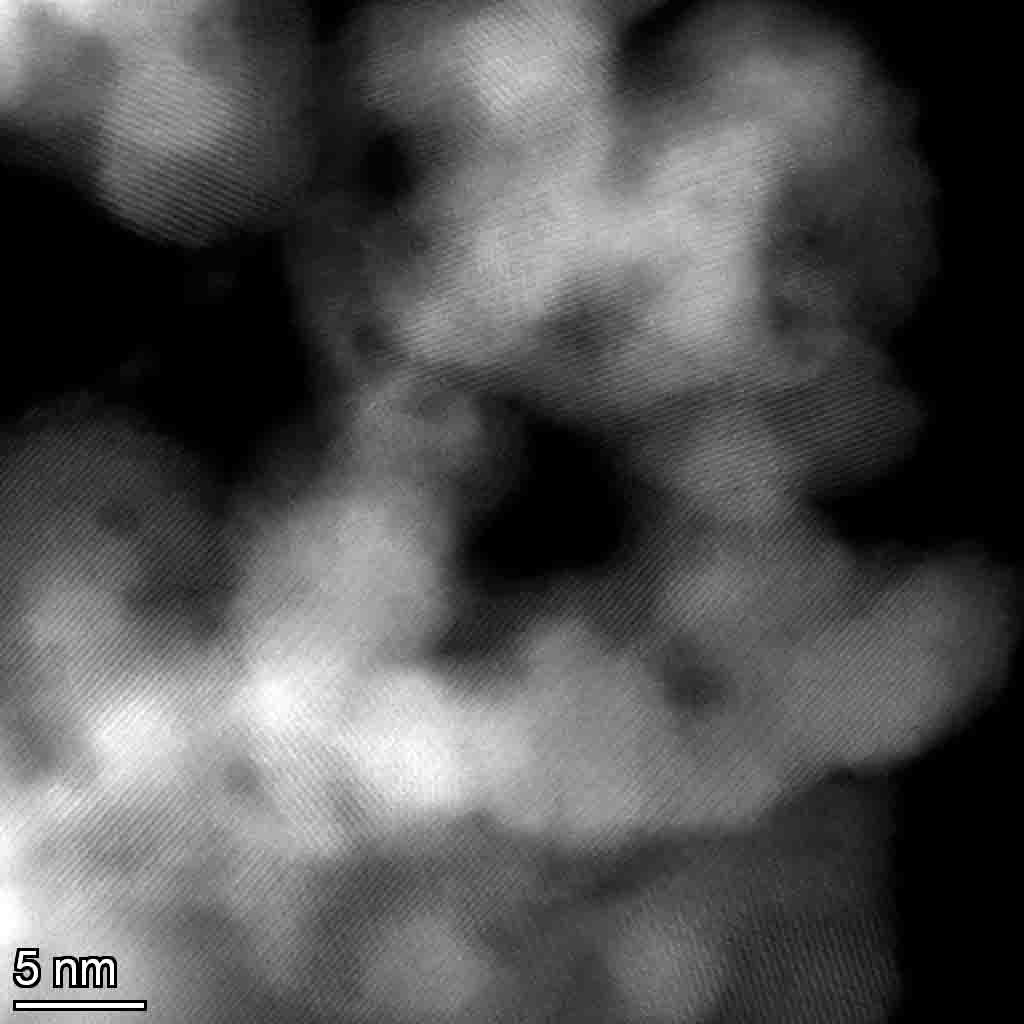

Supplement: Supplementary file 3 — Source Data [file 41467_2023_38336_MOESM3_ESM.zip › Source_Data_for_Figures_in_Supplementary_Information/Source_Data_Supplementary_Figure_22/Supplementary_Figure_22b.jpg]

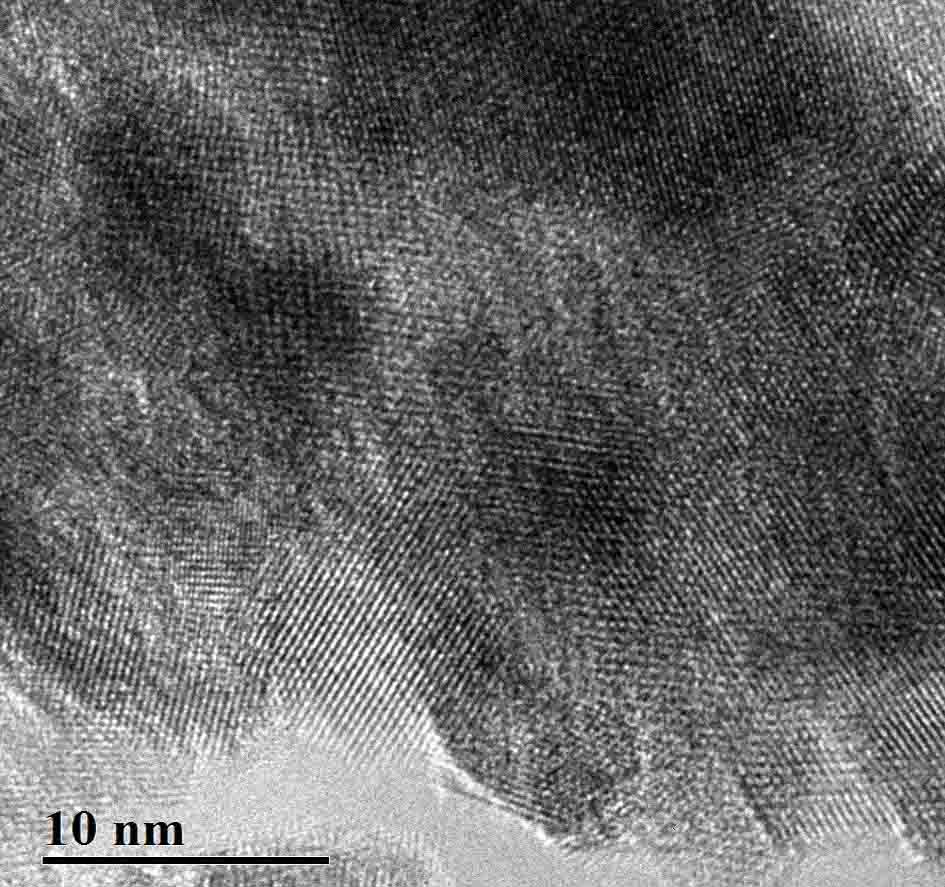

Supplement: Supplementary file 3 — Source Data [file 41467_2023_38336_MOESM3_ESM.zip › Source_Data_for_Figures_in_Supplementary_Information/Source_Data_Supplementary_Figure_22/Supplementary_Figure_22c.jpg]

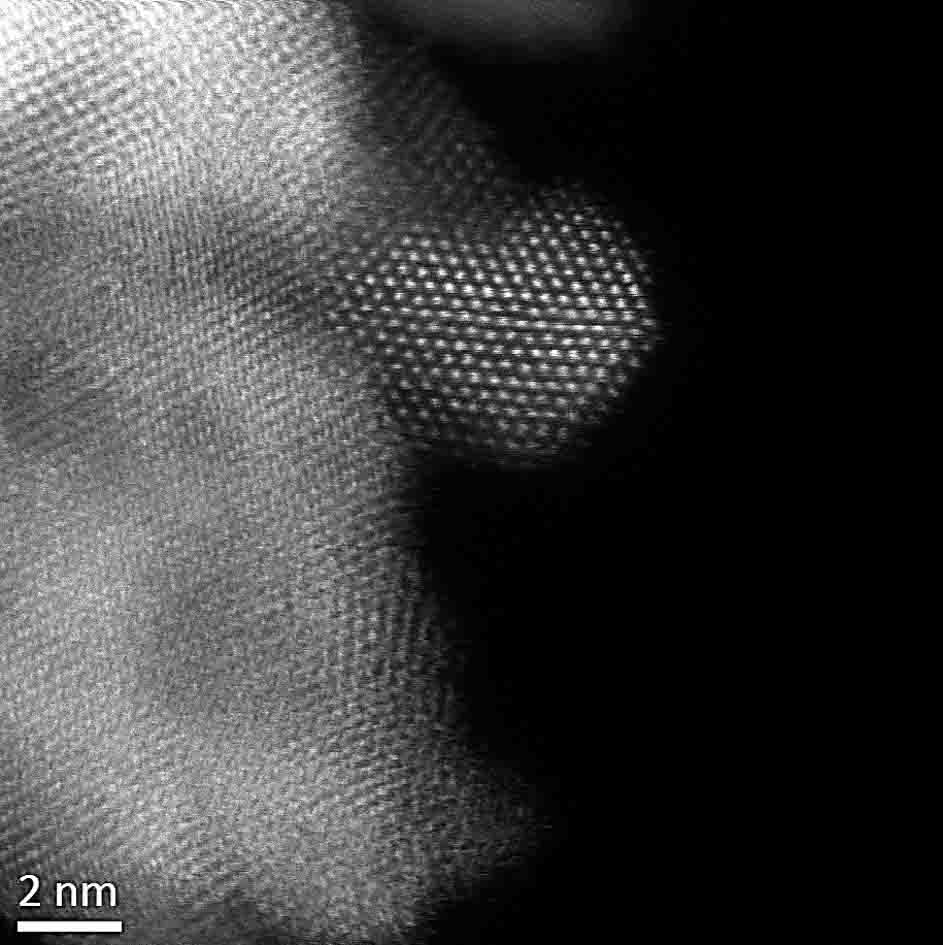

Supplement: Supplementary file 3 — Source Data [file 41467_2023_38336_MOESM3_ESM.zip › Source_Data_for_Figures_in_Supplementary_Information/Source_Data_Supplementary_Figure_22/Supplementary_Figure_22d.jpg]

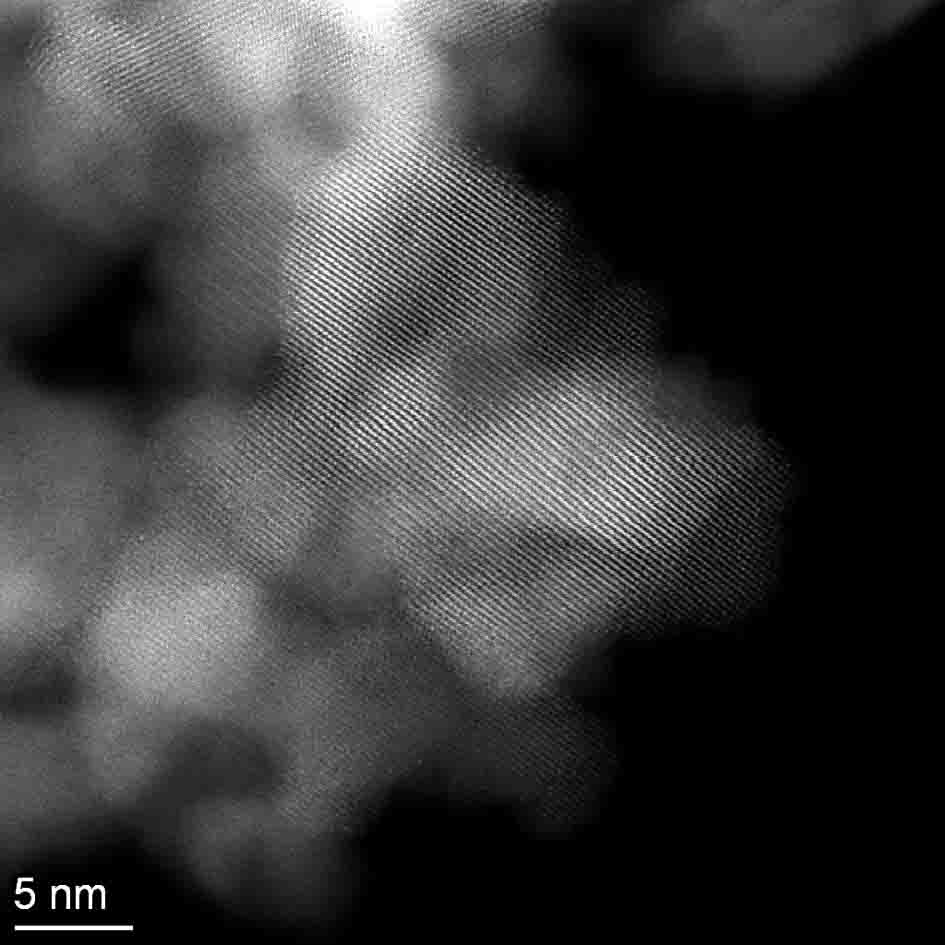

Supplement: Supplementary file 3 — Source Data [file 41467_2023_38336_MOESM3_ESM.zip › Source_Data_for_Figures_in_Supplementary_Information/Source_Data_Supplementary_Figure_22/Supplementary_Figure_22e.jpg]

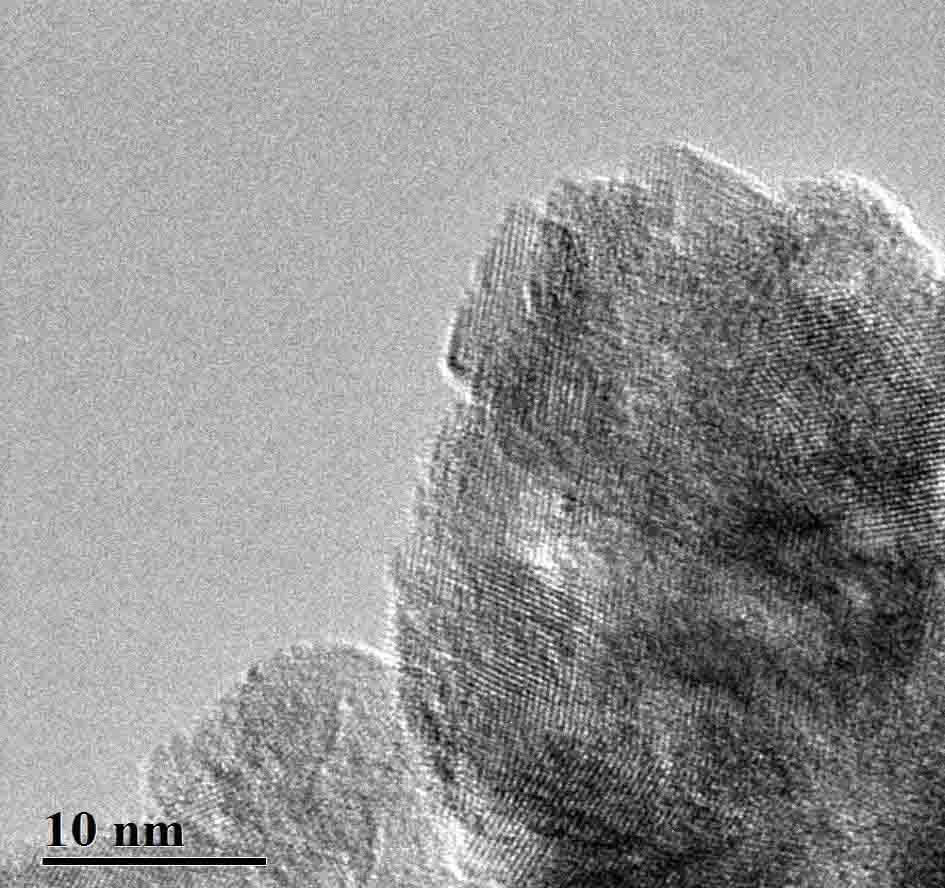

Supplement: Supplementary file 3 — Source Data [file 41467_2023_38336_MOESM3_ESM.zip › Source_Data_for_Figures_in_Supplementary_Information/Source_Data_Supplementary_Figure_22/Supplementary_Figure_22f.jpg]

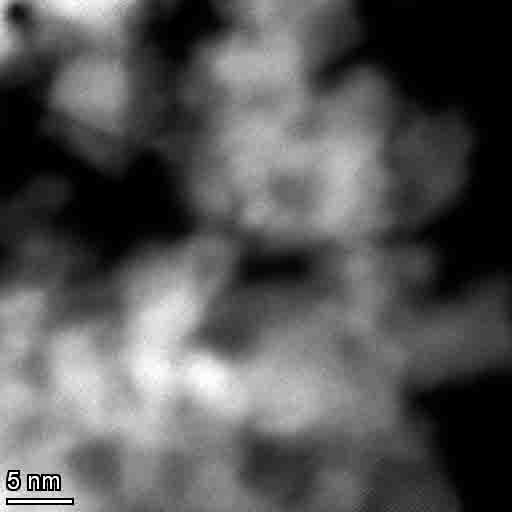

Supplement: Supplementary file 3 — Source Data [file 41467_2023_38336_MOESM3_ESM.zip › Source_Data_for_Figures_in_Supplementary_Information/Source_Data_Supplementary_Figure_23/Supplementary_Figure_23-1.jpg]

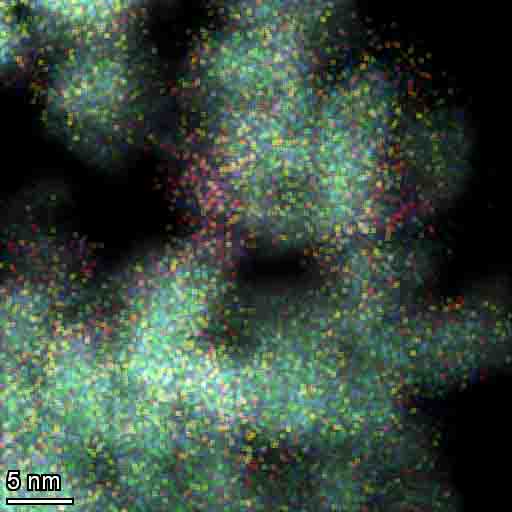

Supplement: Supplementary file 3 — Source Data [file 41467_2023_38336_MOESM3_ESM.zip › Source_Data_for_Figures_in_Supplementary_Information/Source_Data_Supplementary_Figure_23/Supplementary_Figure_23-2.jpg]

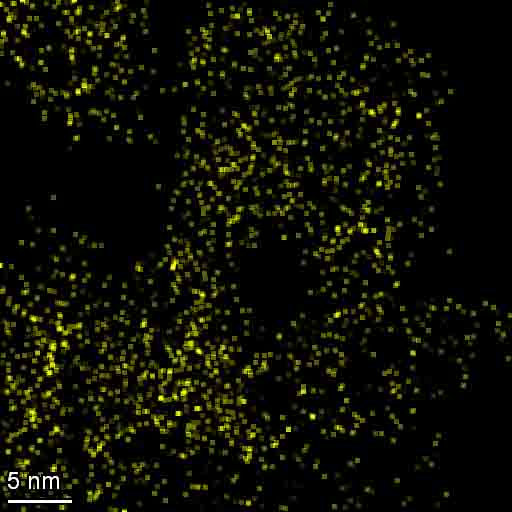

Supplement: Supplementary file 3 — Source Data [file 41467_2023_38336_MOESM3_ESM.zip › Source_Data_for_Figures_in_Supplementary_Information/Source_Data_Supplementary_Figure_23/Supplementary_Figure_23-3.jpg]

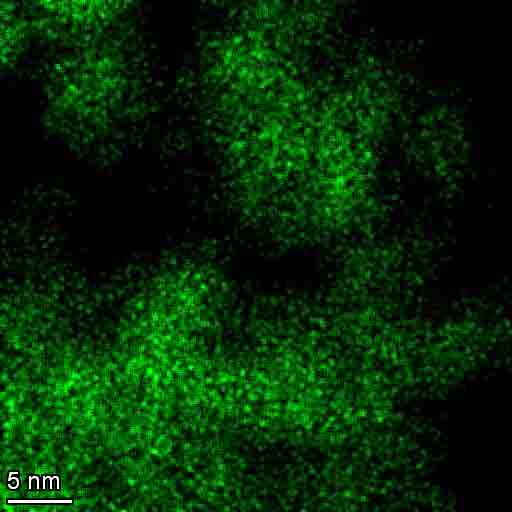

Supplement: Supplementary file 3 — Source Data [file 41467_2023_38336_MOESM3_ESM.zip › Source_Data_for_Figures_in_Supplementary_Information/Source_Data_Supplementary_Figure_23/Supplementary_Figure_23-4.jpg]

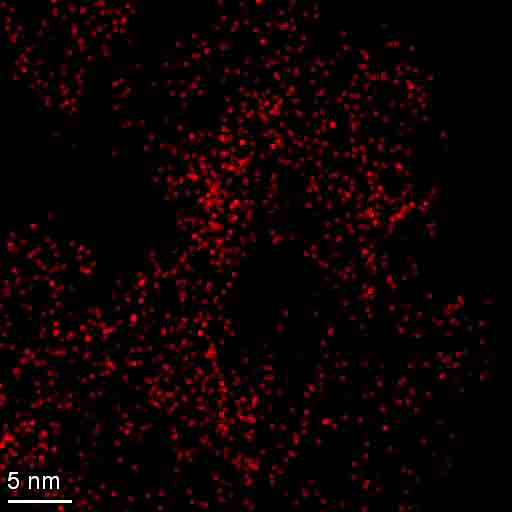

Supplement: Supplementary file 3 — Source Data [file 41467_2023_38336_MOESM3_ESM.zip › Source_Data_for_Figures_in_Supplementary_Information/Source_Data_Supplementary_Figure_23/Supplementary_Figure_23-5.jpg]

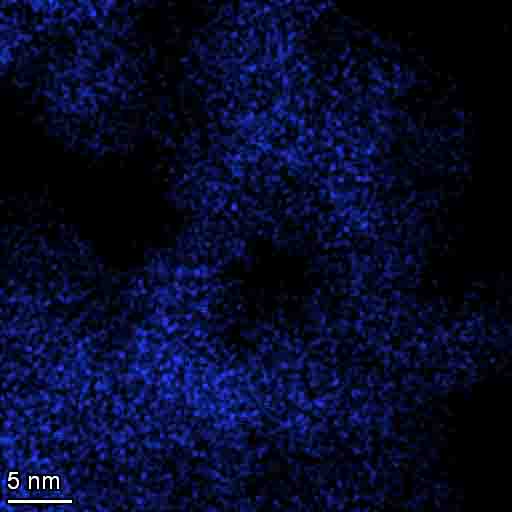

Supplement: Supplementary file 3 — Source Data [file 41467_2023_38336_MOESM3_ESM.zip › Source_Data_for_Figures_in_Supplementary_Information/Source_Data_Supplementary_Figure_23/Supplementary_Figure_23-6.jpg]

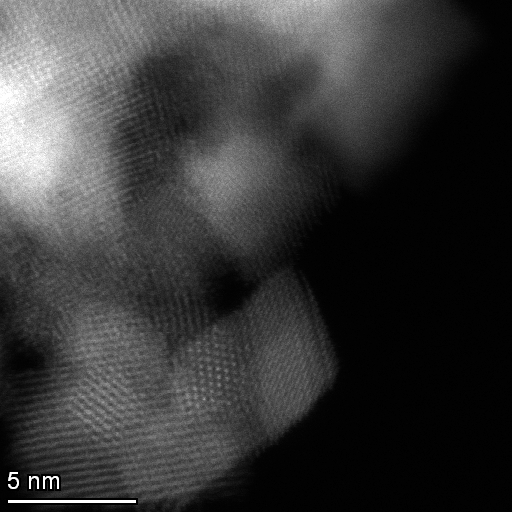

Supplement: Supplementary file 3 — Source Data [file 41467_2023_38336_MOESM3_ESM.zip › Source_Data_for_Figures_in_Supplementary_Information/Source_Data_Supplementary_Figure_24/Supplementary_Figure_24-1.tif]

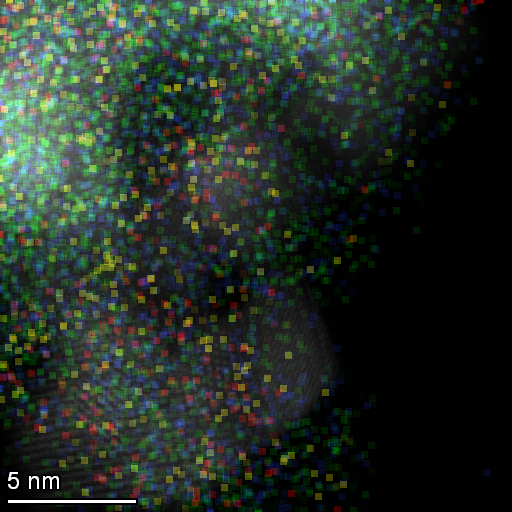

Supplement: Supplementary file 3 — Source Data [file 41467_2023_38336_MOESM3_ESM.zip › Source_Data_for_Figures_in_Supplementary_Information/Source_Data_Supplementary_Figure_24/Supplementary_Figure_24-2.tif]

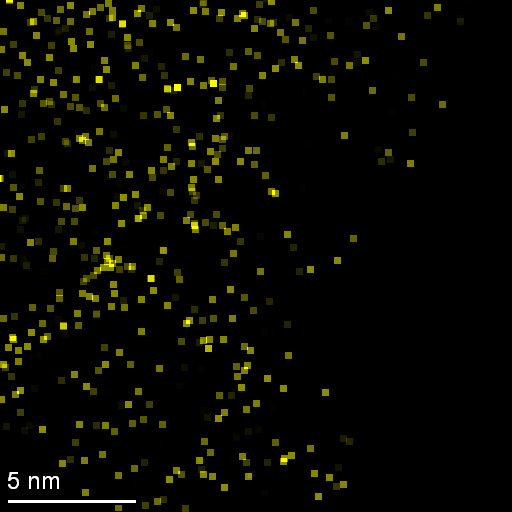

Supplement: Supplementary file 3 — Source Data [file 41467_2023_38336_MOESM3_ESM.zip › Source_Data_for_Figures_in_Supplementary_Information/Source_Data_Supplementary_Figure_24/Supplementary_Figure_24-3.tif]

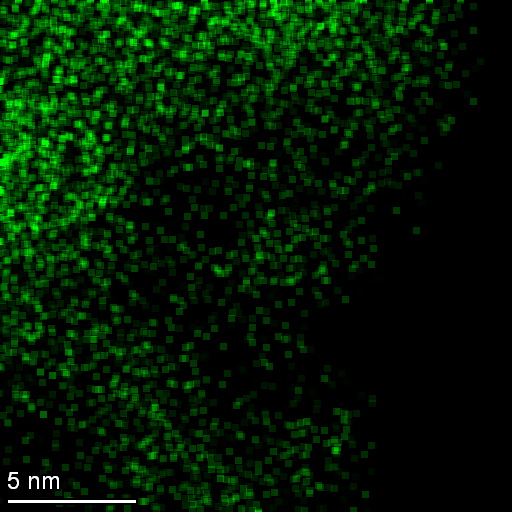

Supplement: Supplementary file 3 — Source Data [file 41467_2023_38336_MOESM3_ESM.zip › Source_Data_for_Figures_in_Supplementary_Information/Source_Data_Supplementary_Figure_24/Supplementary_Figure_24-4.tif]

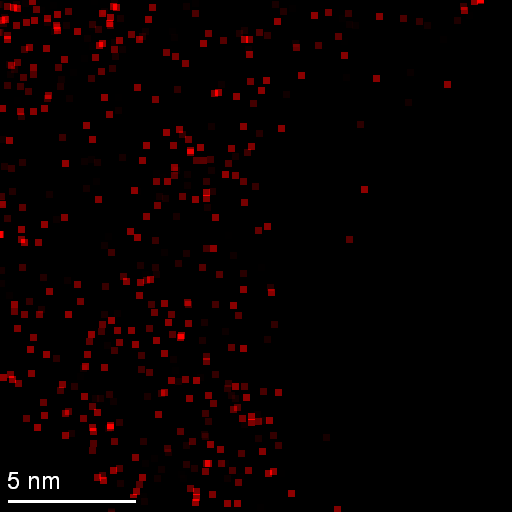

Supplement: Supplementary file 3 — Source Data [file 41467_2023_38336_MOESM3_ESM.zip › Source_Data_for_Figures_in_Supplementary_Information/Source_Data_Supplementary_Figure_24/Supplementary_Figure_24-5.tif]

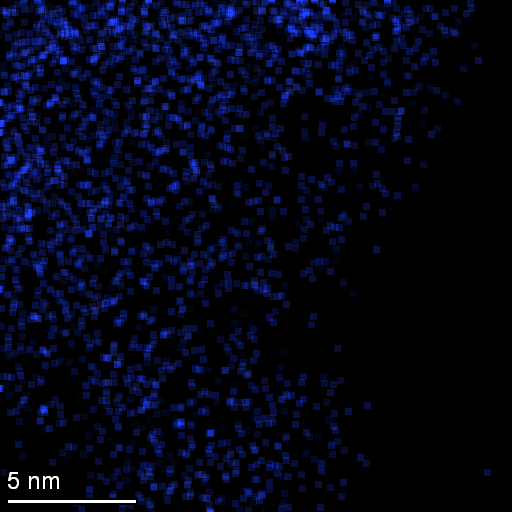

Supplement: Supplementary file 3 — Source Data [file 41467_2023_38336_MOESM3_ESM.zip › Source_Data_for_Figures_in_Supplementary_Information/Source_Data_Supplementary_Figure_24/Supplementary_Figure_24-6.tif]

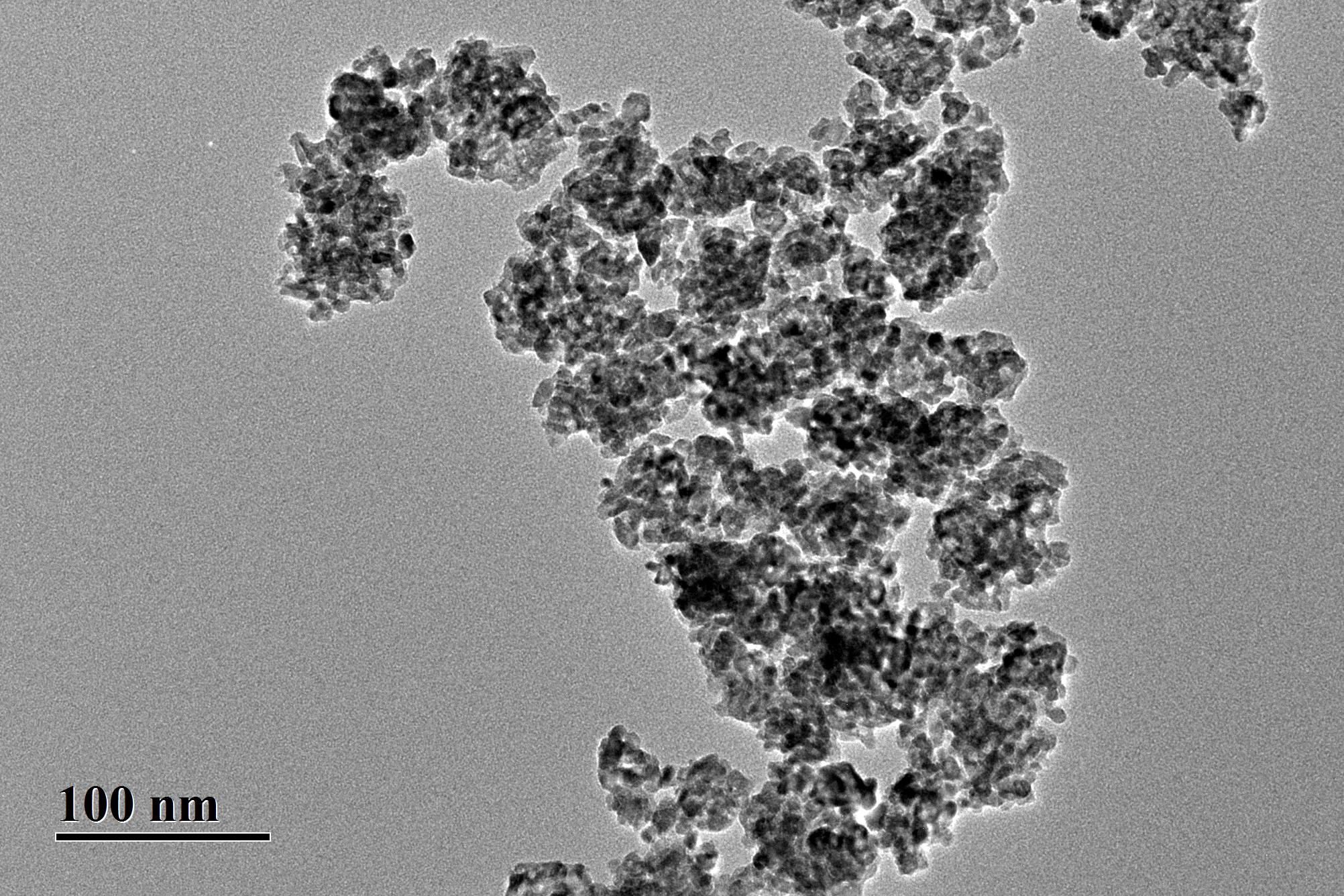

Supplement: Supplementary file 3 — Source Data [file 41467_2023_38336_MOESM3_ESM.zip › Source_Data_for_Figures_in_Supplementary_Information/Source_Data_Supplementary_Figure_26/Supplementary_Figure_26a.jpg]

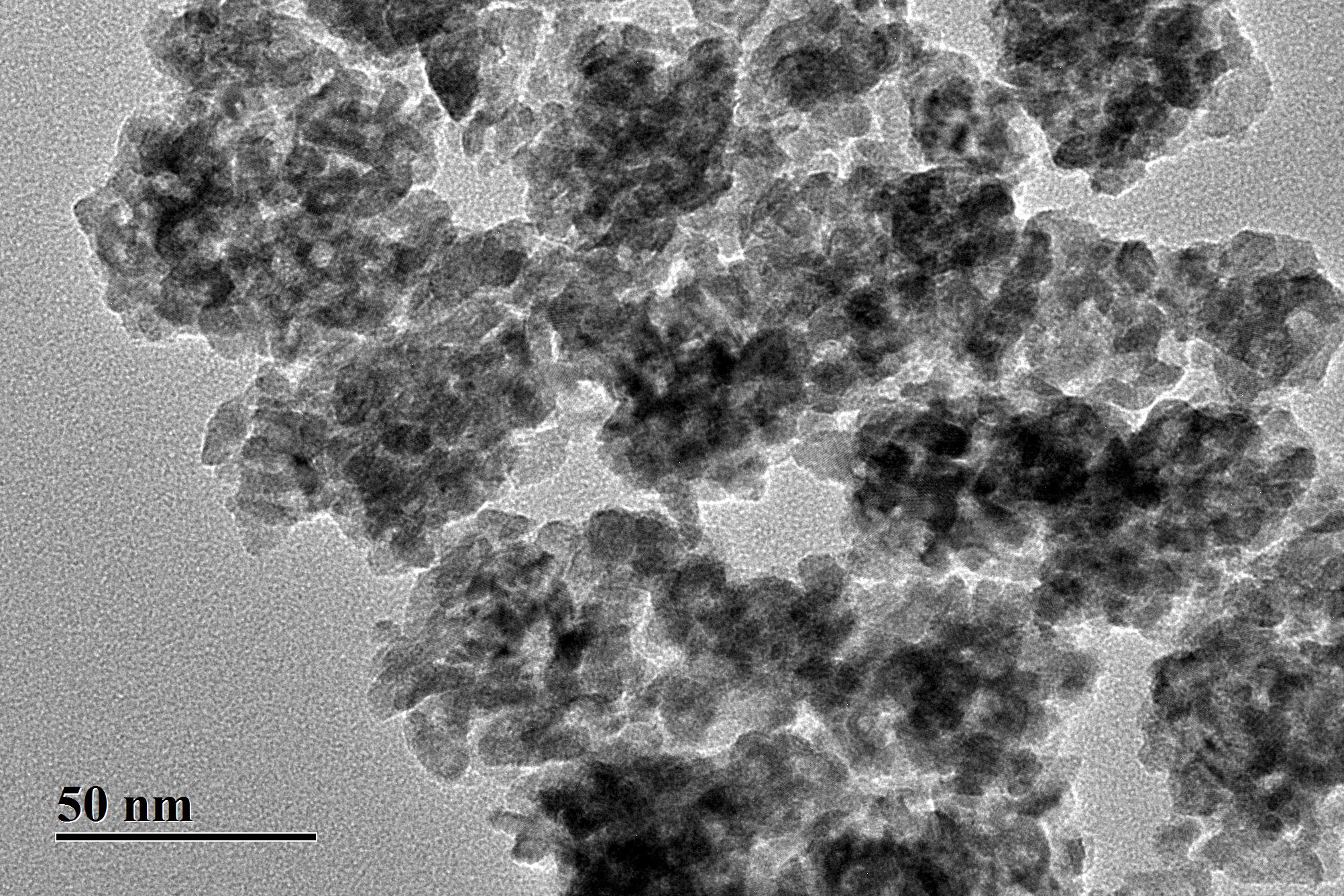

Supplement: Supplementary file 3 — Source Data [file 41467_2023_38336_MOESM3_ESM.zip › Source_Data_for_Figures_in_Supplementary_Information/Source_Data_Supplementary_Figure_26/Supplementary_Figure_26b.jpg]

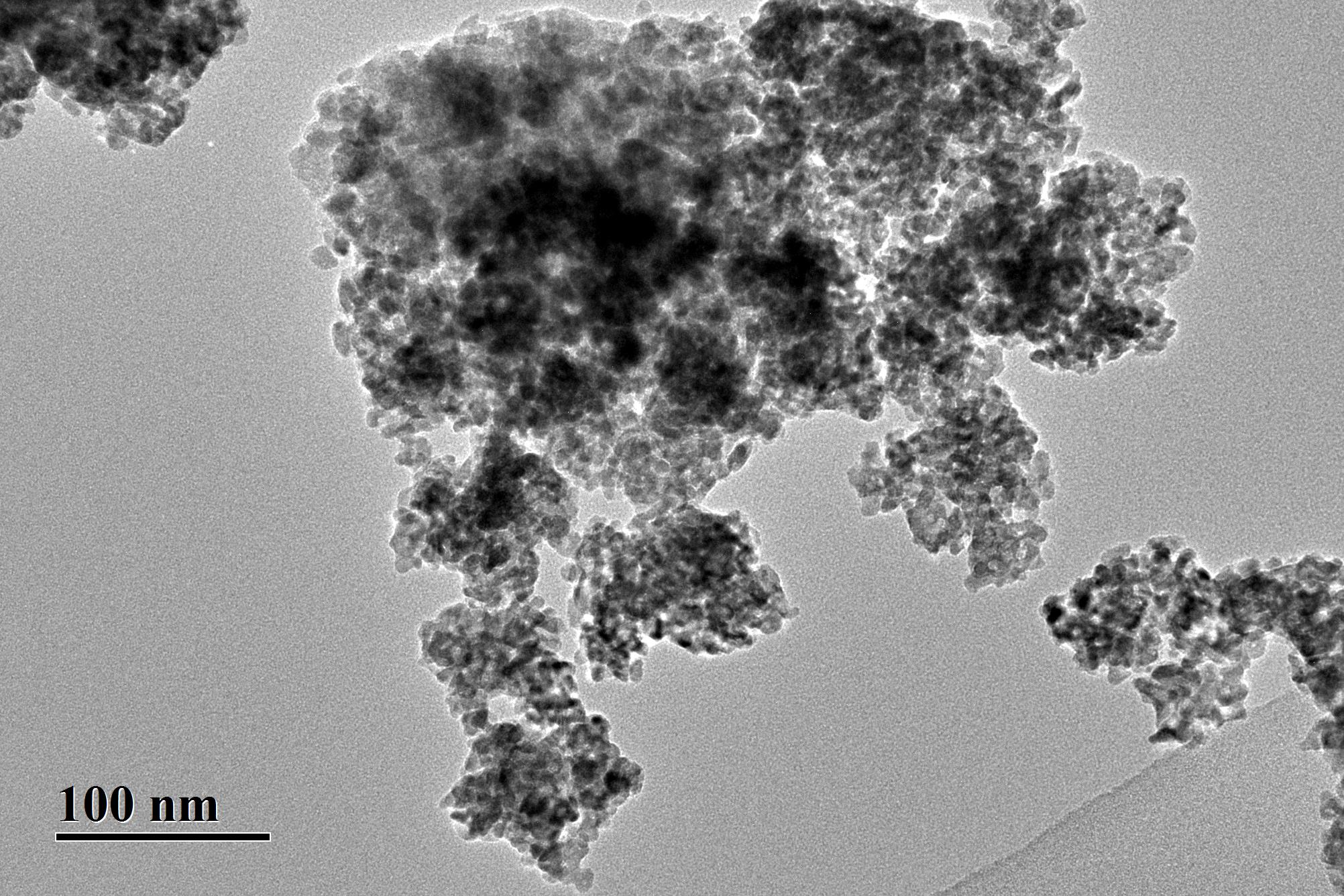

Supplement: Supplementary file 3 — Source Data [file 41467_2023_38336_MOESM3_ESM.zip › Source_Data_for_Figures_in_Supplementary_Information/Source_Data_Supplementary_Figure_26/Supplementary_Figure_26d.jpg]

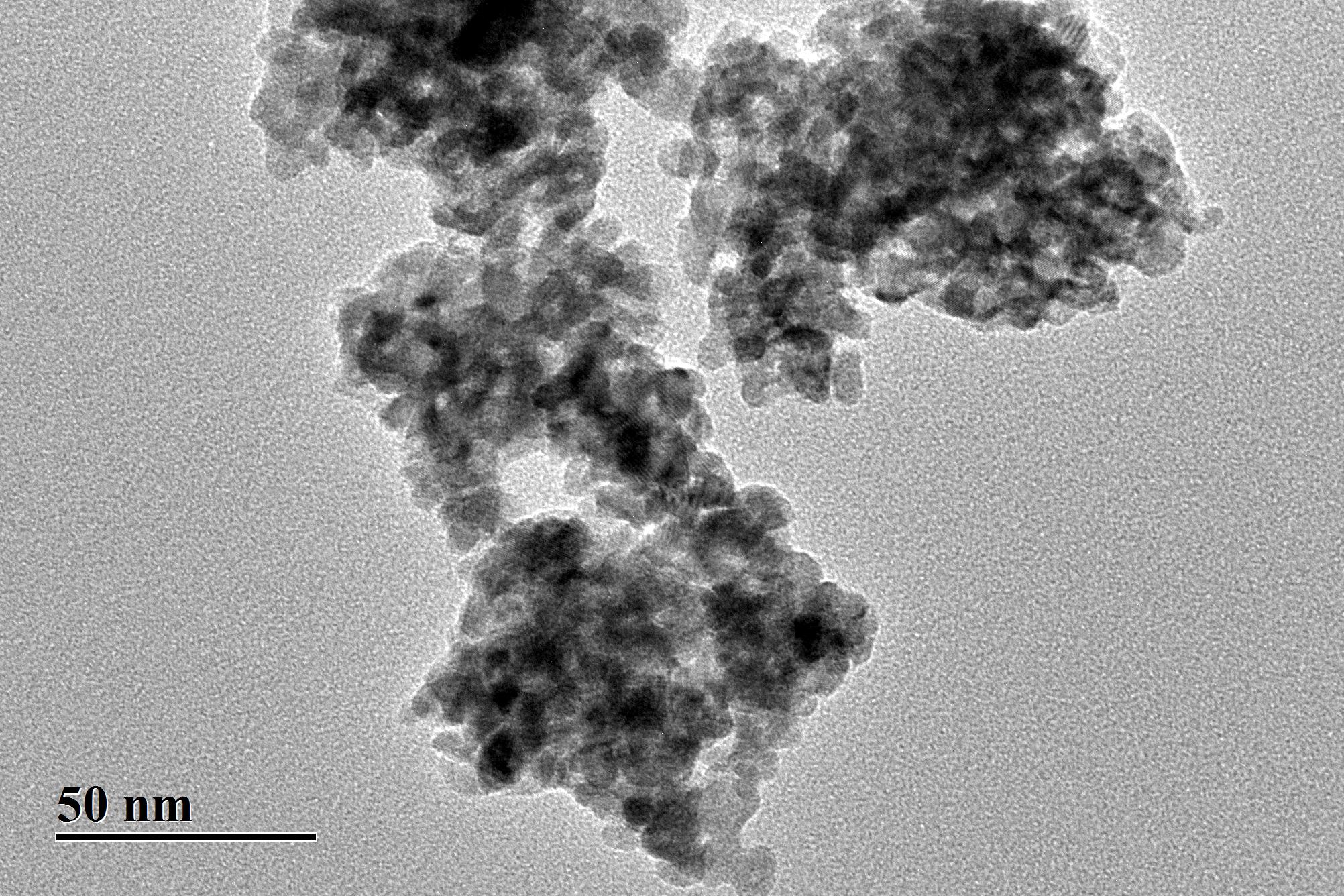

Supplement: Supplementary file 3 — Source Data [file 41467_2023_38336_MOESM3_ESM.zip › Source_Data_for_Figures_in_Supplementary_Information/Source_Data_Supplementary_Figure_26/Supplementary_Figure_26e.jpg]

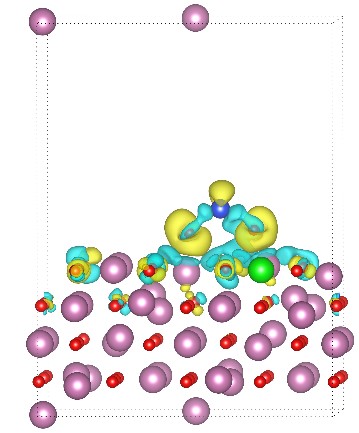

Supplement: Supplementary file 3 — Source Data [file 41467_2023_38336_MOESM3_ESM.zip › Source_Data_for_Figures_in_Supplementary_Information/Source_Data_Supplementary_Figure_27/Supplementary_Figure_27b.jpg]

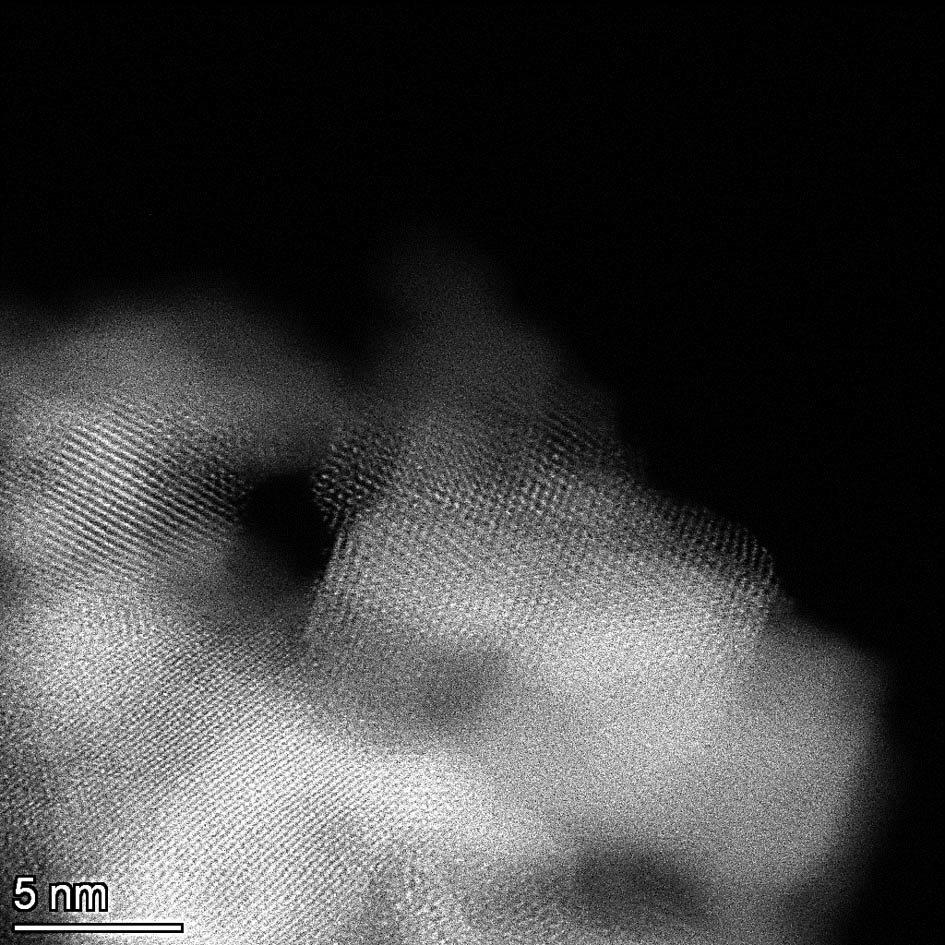

Supplement: Supplementary file 3 — Source Data [file 41467_2023_38336_MOESM3_ESM.zip › Source_Data_for_Figures_in_Supplementary_Information/Source_Data_Supplementary_Figure_34/Supplementary_Figure_34a.jpg]

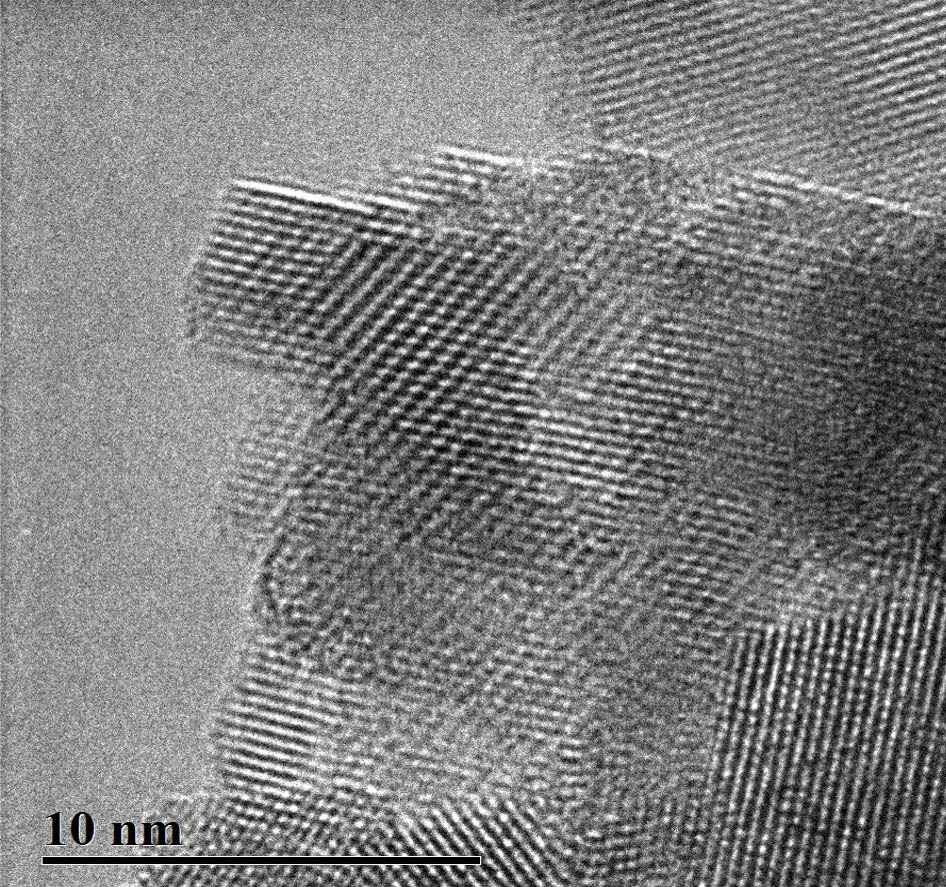

Supplement: Supplementary file 3 — Source Data [file 41467_2023_38336_MOESM3_ESM.zip › Source_Data_for_Figures_in_Supplementary_Information/Source_Data_Supplementary_Figure_34/Supplementary_Figure_34b.jpg]

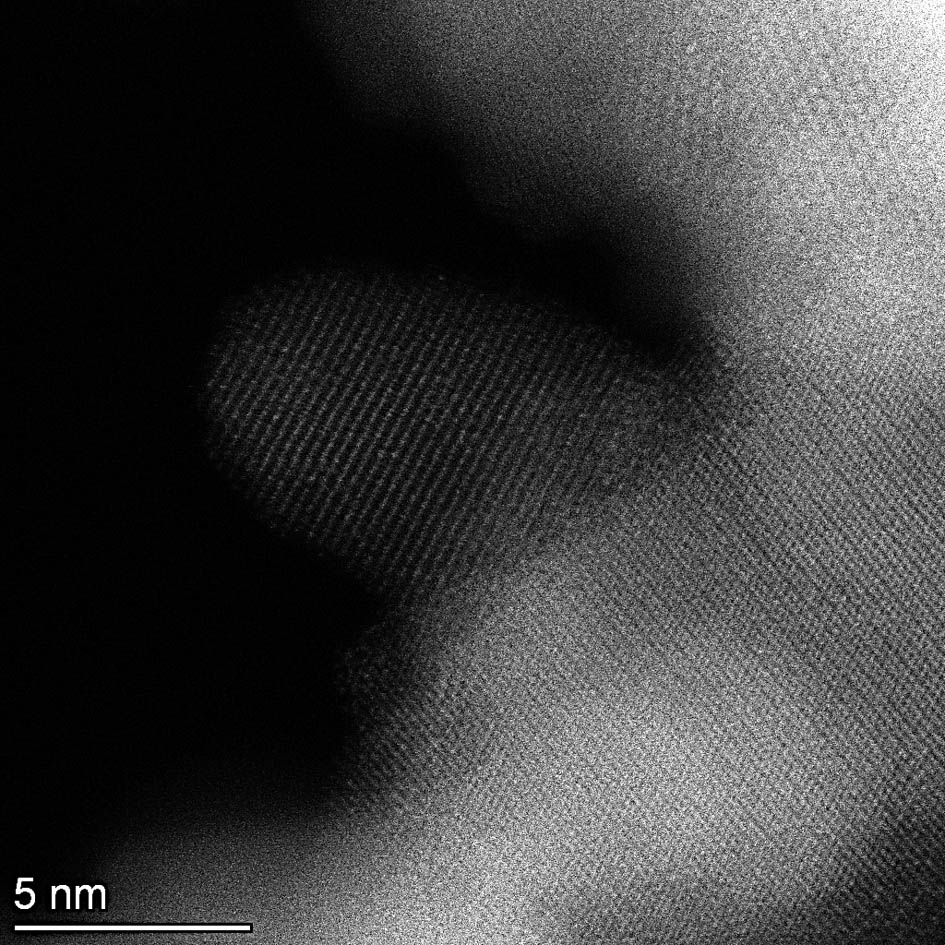

Supplement: Supplementary file 3 — Source Data [file 41467_2023_38336_MOESM3_ESM.zip › Source_Data_for_Figures_in_Supplementary_Information/Source_Data_Supplementary_Figure_34/Supplementary_Figure_34c.jpg]

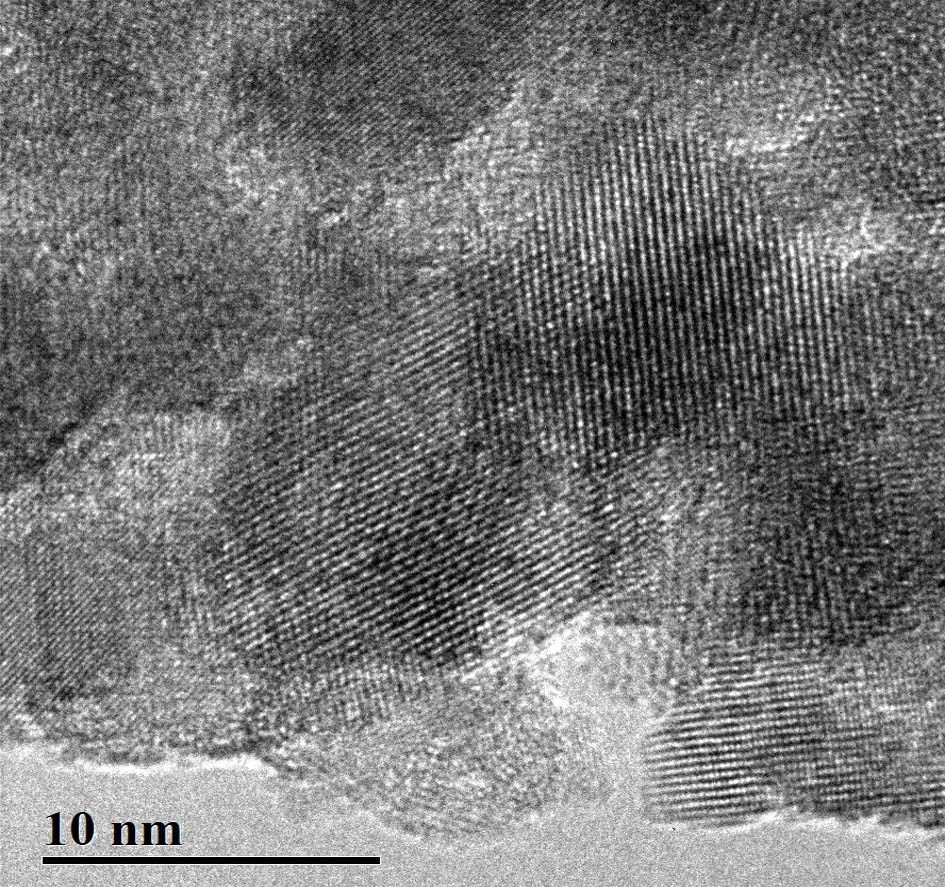

Supplement: Supplementary file 3 — Source Data [file 41467_2023_38336_MOESM3_ESM.zip › Source_Data_for_Figures_in_Supplementary_Information/Source_Data_Supplementary_Figure_34/Supplementary_Figure_34d.jpg]

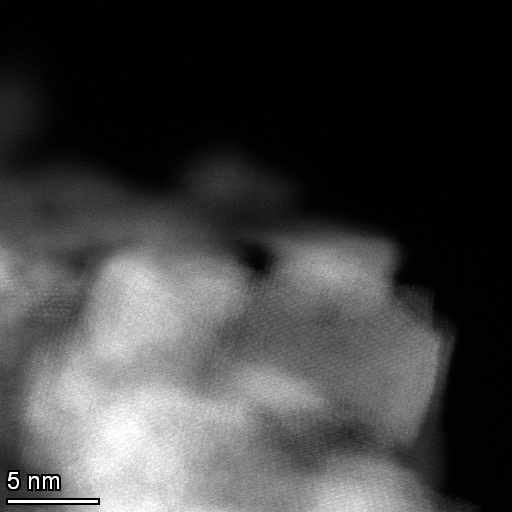

Supplement: Supplementary file 3 — Source Data [file 41467_2023_38336_MOESM3_ESM.zip › Source_Data_for_Figures_in_Supplementary_Information/Source_Data_Supplementary_Figure_35/Supplementary_Figure_35-1.tif]

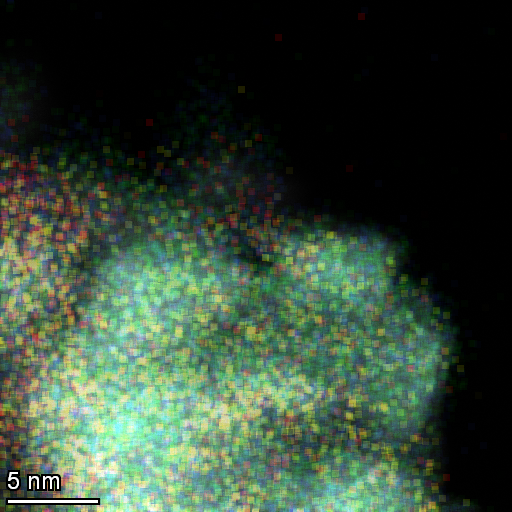

Supplement: Supplementary file 3 — Source Data [file 41467_2023_38336_MOESM3_ESM.zip › Source_Data_for_Figures_in_Supplementary_Information/Source_Data_Supplementary_Figure_35/Supplementary_Figure_35-2.tif]

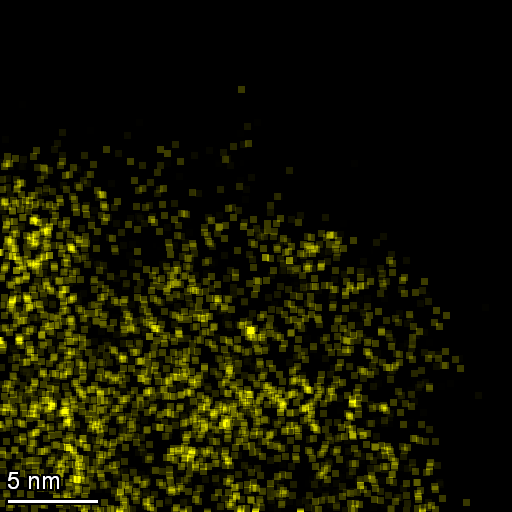

Supplement: Supplementary file 3 — Source Data [file 41467_2023_38336_MOESM3_ESM.zip › Source_Data_for_Figures_in_Supplementary_Information/Source_Data_Supplementary_Figure_35/Supplementary_Figure_35-3.tif]

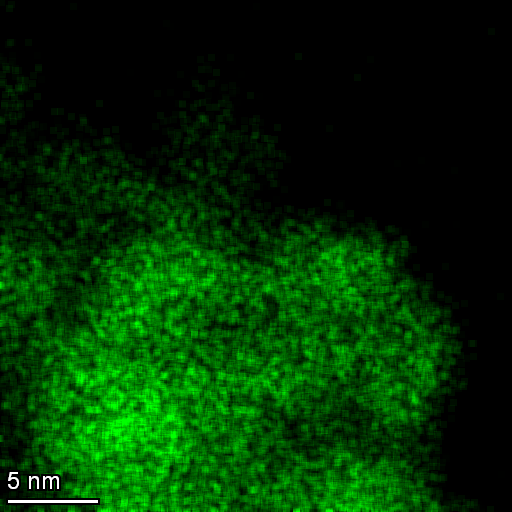

Supplement: Supplementary file 3 — Source Data [file 41467_2023_38336_MOESM3_ESM.zip › Source_Data_for_Figures_in_Supplementary_Information/Source_Data_Supplementary_Figure_35/Supplementary_Figure_35-4.tif]

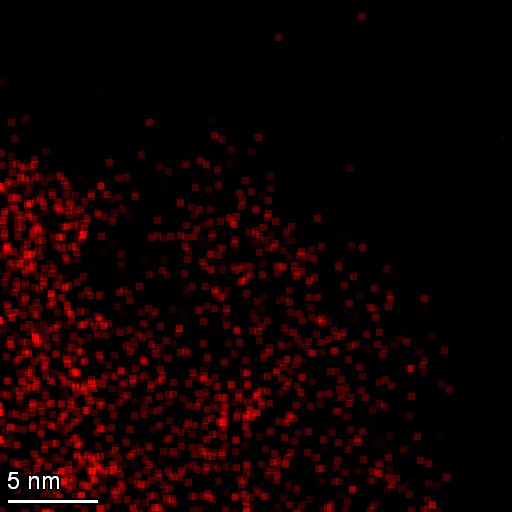

Supplement: Supplementary file 3 — Source Data [file 41467_2023_38336_MOESM3_ESM.zip › Source_Data_for_Figures_in_Supplementary_Information/Source_Data_Supplementary_Figure_35/Supplementary_Figure_35-5.tif]

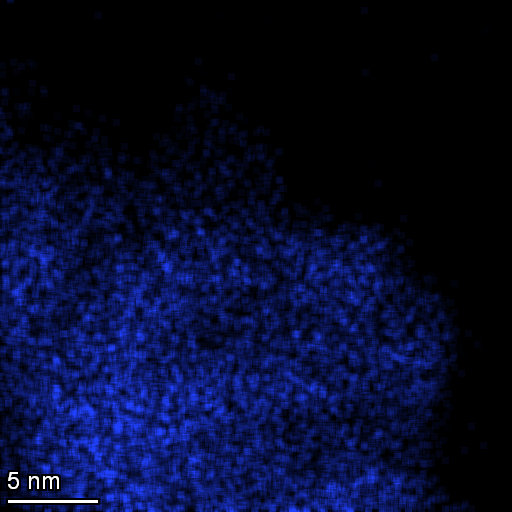

Supplement: Supplementary file 3 — Source Data [file 41467_2023_38336_MOESM3_ESM.zip › Source_Data_for_Figures_in_Supplementary_Information/Source_Data_Supplementary_Figure_35/Supplementary_Figure_35-6.tif]

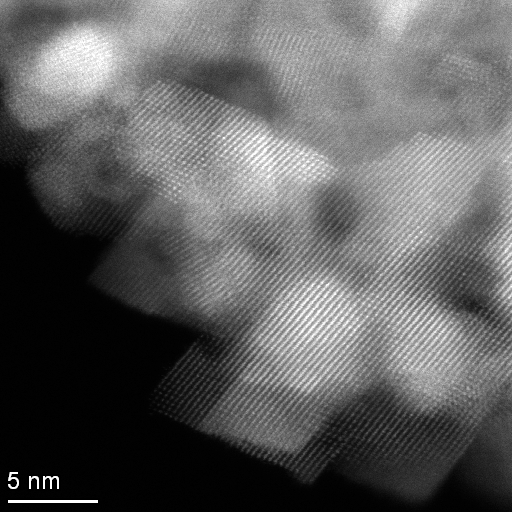

Supplement: Supplementary file 3 — Source Data [file 41467_2023_38336_MOESM3_ESM.zip › Source_Data_for_Figures_in_Supplementary_Information/Source_Data_Supplementary_Figure_36/Supplementary_Figure_36-1.tif]

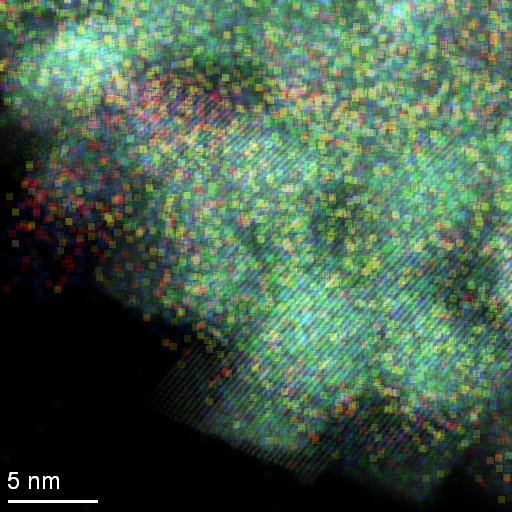

Supplement: Supplementary file 3 — Source Data [file 41467_2023_38336_MOESM3_ESM.zip › Source_Data_for_Figures_in_Supplementary_Information/Source_Data_Supplementary_Figure_36/Supplementary_Figure_36-2.tif]

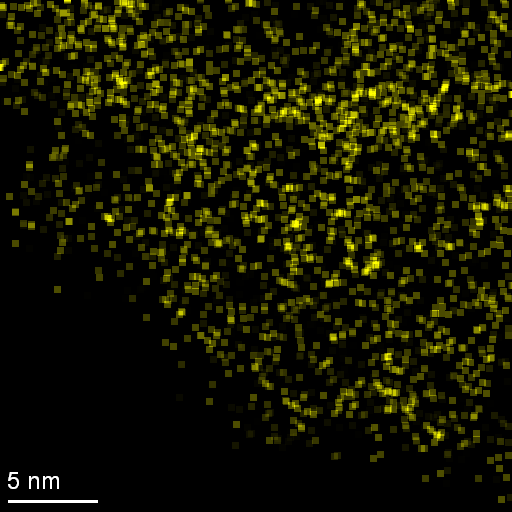

Supplement: Supplementary file 3 — Source Data [file 41467_2023_38336_MOESM3_ESM.zip › Source_Data_for_Figures_in_Supplementary_Information/Source_Data_Supplementary_Figure_36/Supplementary_Figure_36-3.tif]

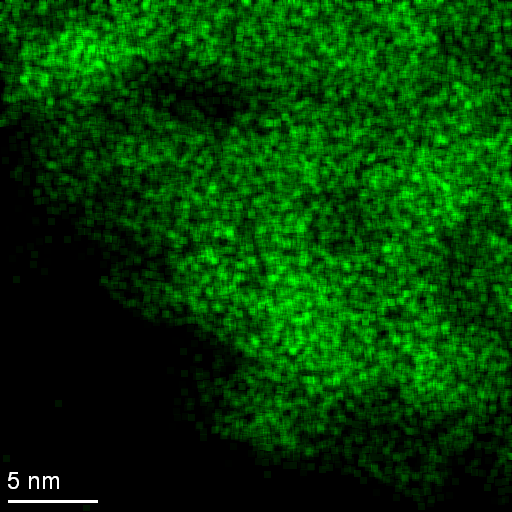

Supplement: Supplementary file 3 — Source Data [file 41467_2023_38336_MOESM3_ESM.zip › Source_Data_for_Figures_in_Supplementary_Information/Source_Data_Supplementary_Figure_36/Supplementary_Figure_36-4.tif]

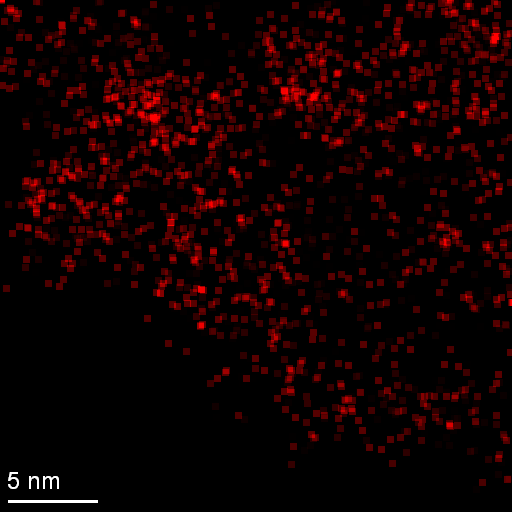

Supplement: Supplementary file 3 — Source Data [file 41467_2023_38336_MOESM3_ESM.zip › Source_Data_for_Figures_in_Supplementary_Information/Source_Data_Supplementary_Figure_36/Supplementary_Figure_36-5.tif]

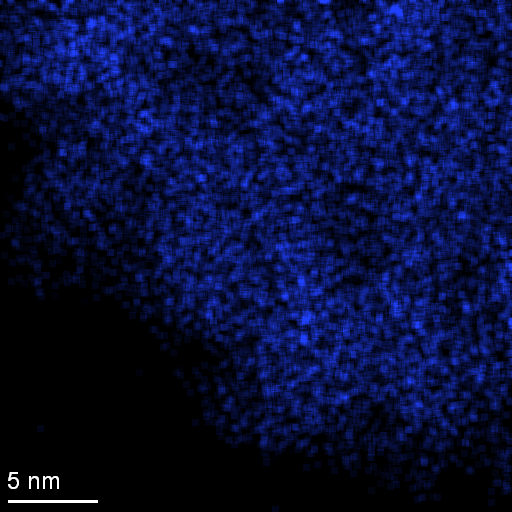

Supplement: Supplementary file 3 — Source Data [file 41467_2023_38336_MOESM3_ESM.zip › Source_Data_for_Figures_in_Supplementary_Information/Source_Data_Supplementary_Figure_36/Supplementary_Figure_36-6.tif]

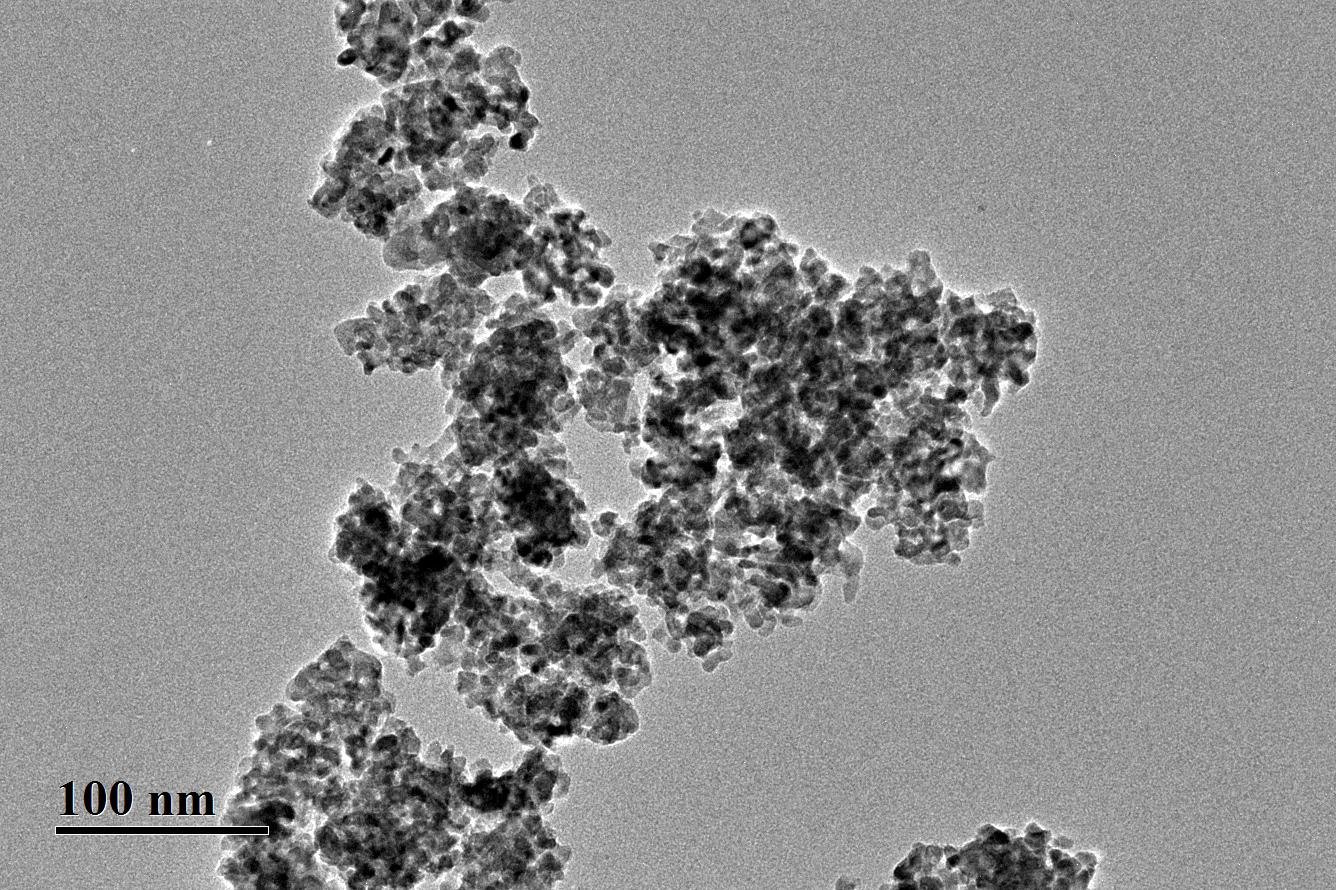

Supplement: Supplementary file 3 — Source Data [file 41467_2023_38336_MOESM3_ESM.zip › Source_Data_for_Figures_in_Supplementary_Information/Source_Data_Supplementary_Figure_37/Supplementary_Figure_37a.jpg]

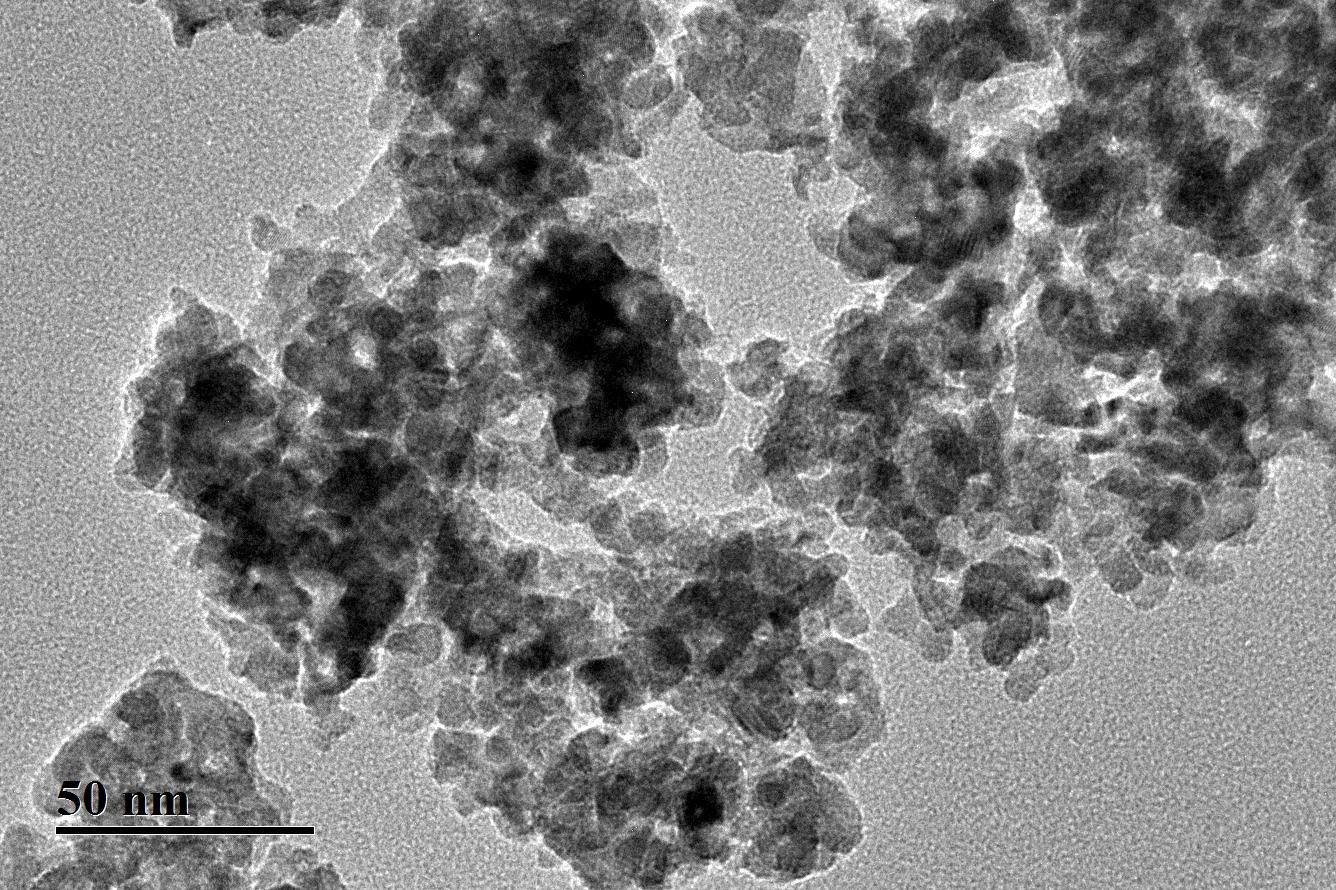

Supplement: Supplementary file 3 — Source Data [file 41467_2023_38336_MOESM3_ESM.zip › Source_Data_for_Figures_in_Supplementary_Information/Source_Data_Supplementary_Figure_37/Supplementary_Figure_37b.jpg]

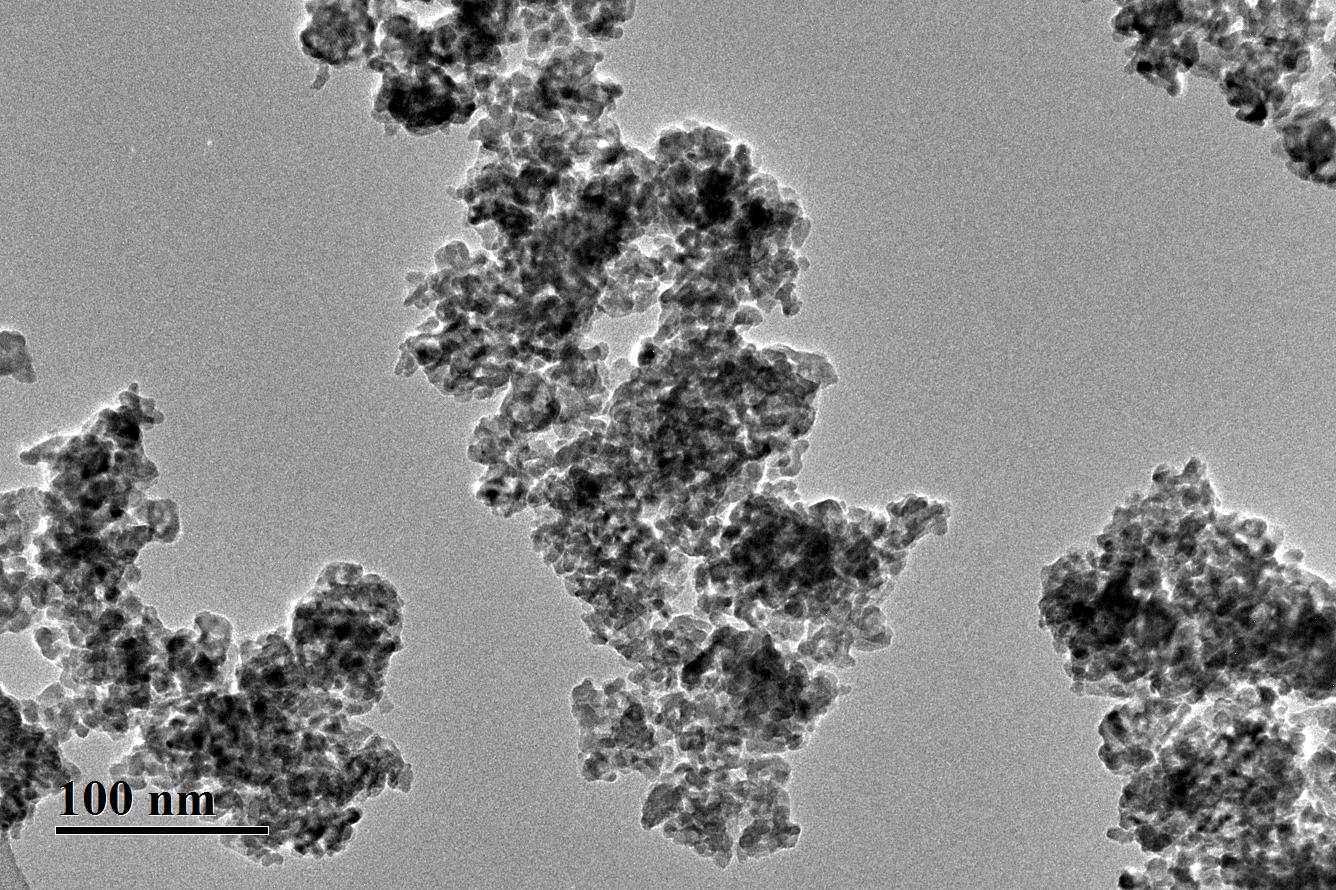

Supplement: Supplementary file 3 — Source Data [file 41467_2023_38336_MOESM3_ESM.zip › Source_Data_for_Figures_in_Supplementary_Information/Source_Data_Supplementary_Figure_37/Supplementary_Figure_37d.jpg]

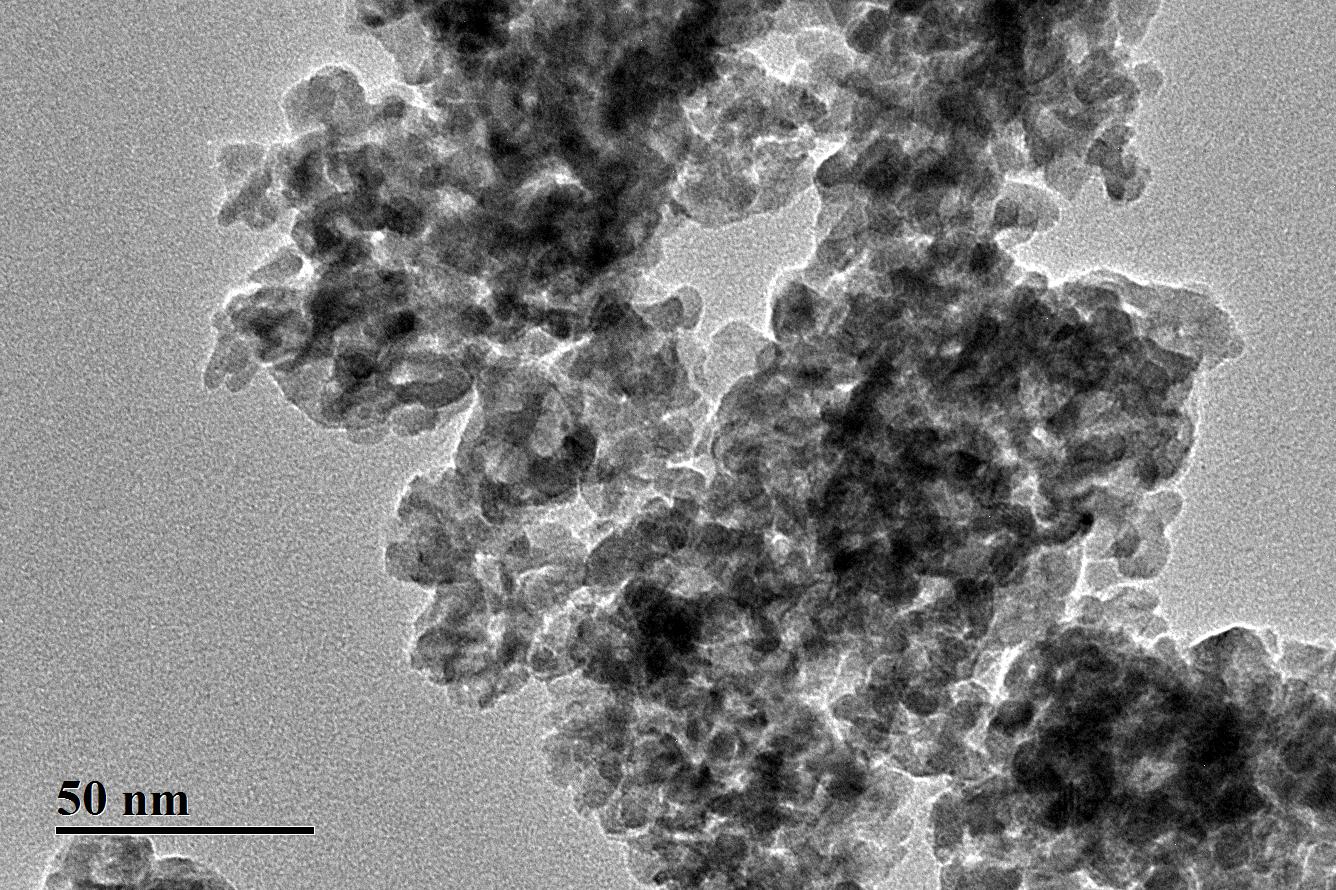

Supplement: Supplementary file 3 — Source Data [file 41467_2023_38336_MOESM3_ESM.zip › Source_Data_for_Figures_in_Supplementary_Information/Source_Data_Supplementary_Figure_37/Supplementary_Figure_37e.jpg]

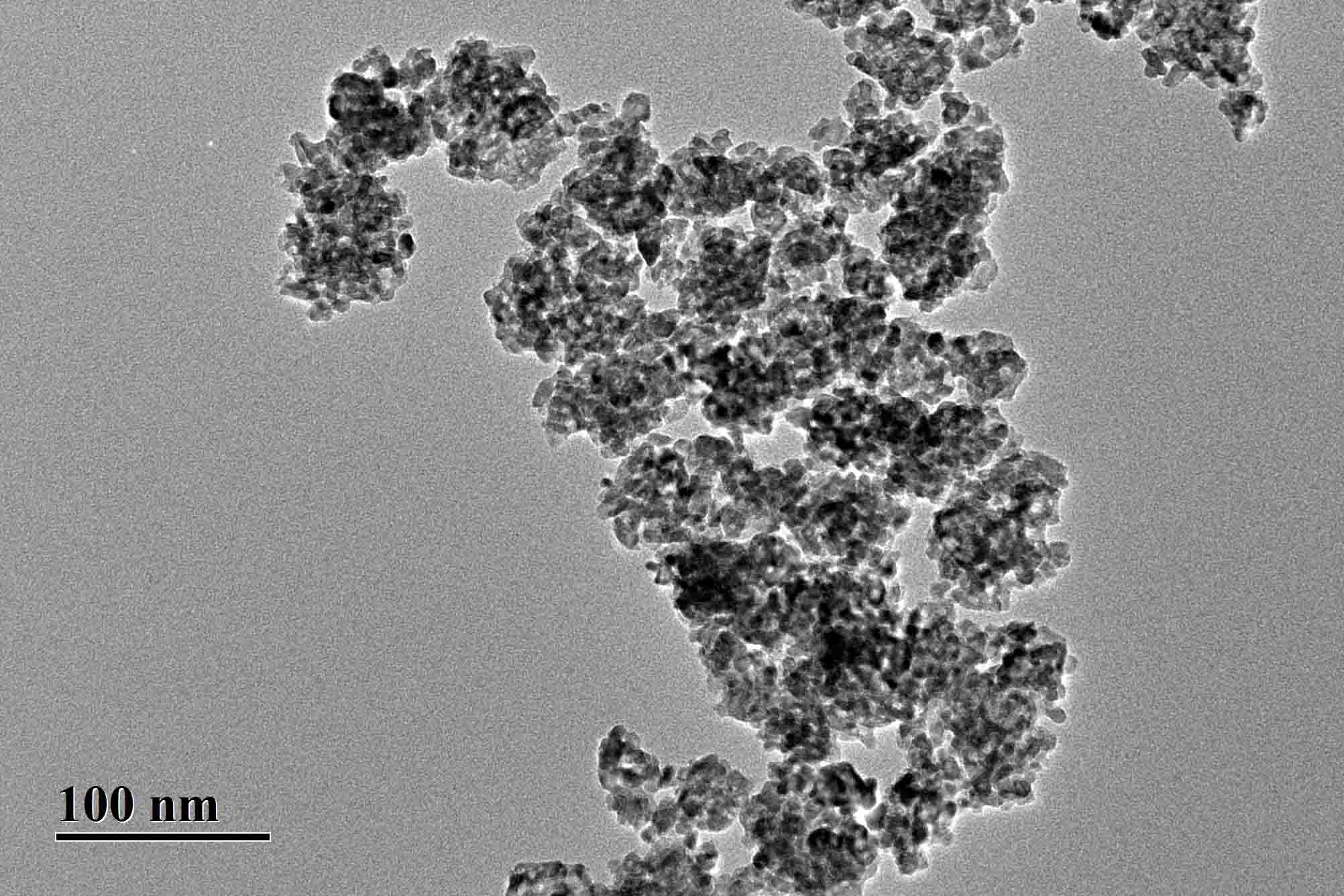

Supplement: Supplementary file 3 — Source Data [file 41467_2023_38336_MOESM3_ESM.zip › Source_Data_for_Figures_in_Supplementary_Information/Source_Data_Supplementary_Figure_39/Supplementary_Figure_39a.jpg]

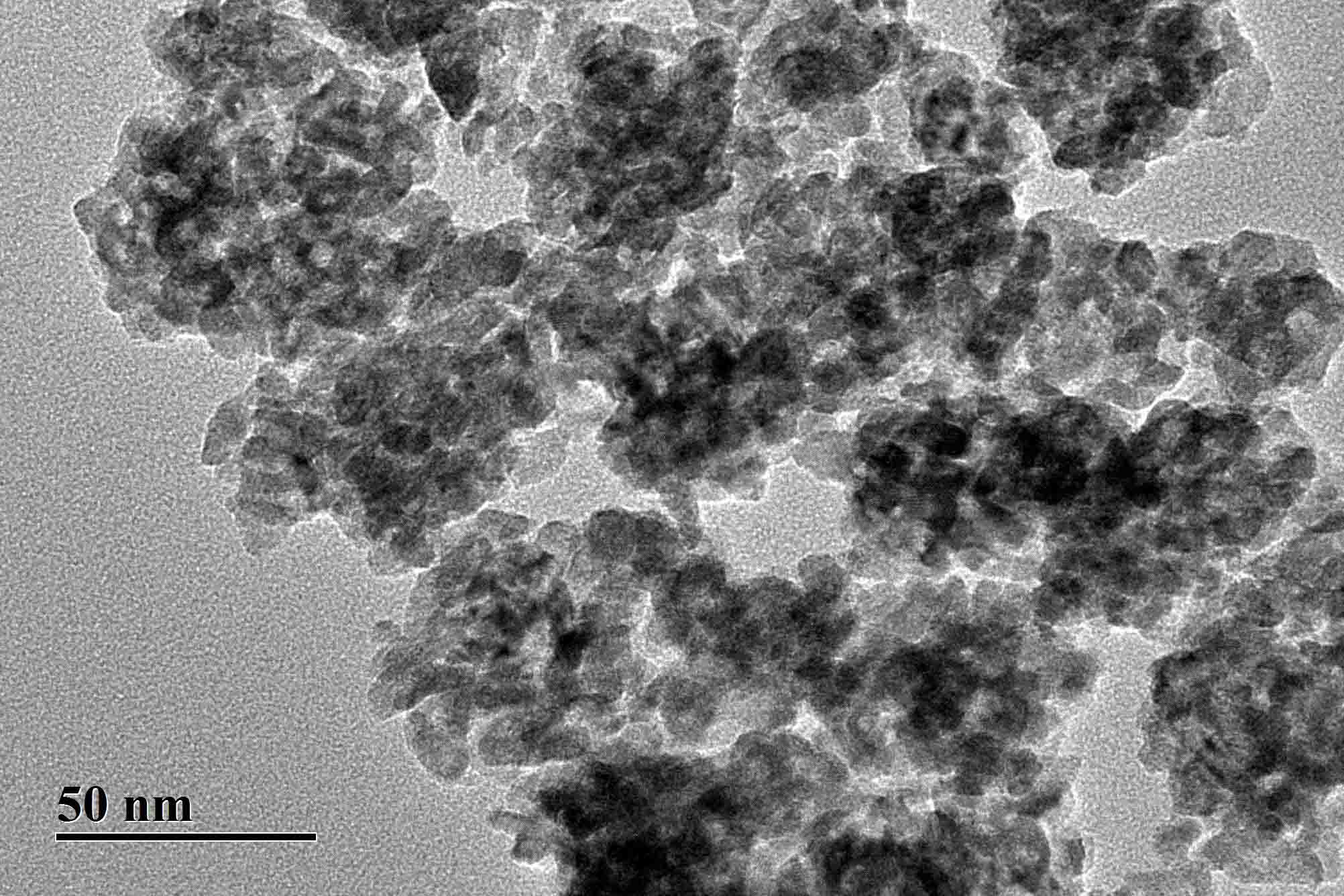

Supplement: Supplementary file 3 — Source Data [file 41467_2023_38336_MOESM3_ESM.zip › Source_Data_for_Figures_in_Supplementary_Information/Source_Data_Supplementary_Figure_39/Supplementary_Figure_39b.jpg]

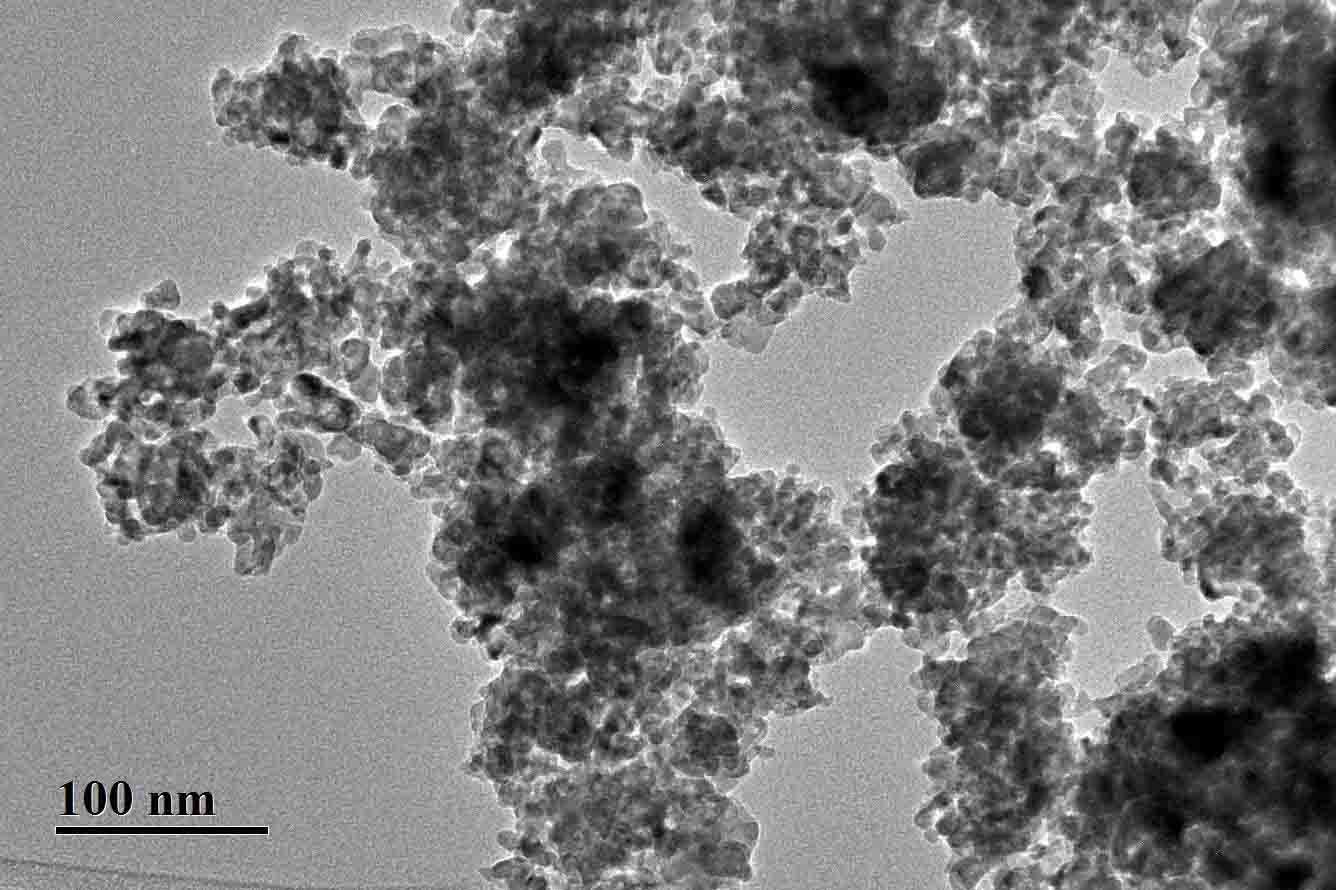

Supplement: Supplementary file 3 — Source Data [file 41467_2023_38336_MOESM3_ESM.zip › Source_Data_for_Figures_in_Supplementary_Information/Source_Data_Supplementary_Figure_39/Supplementary_Figure_39d.jpg]

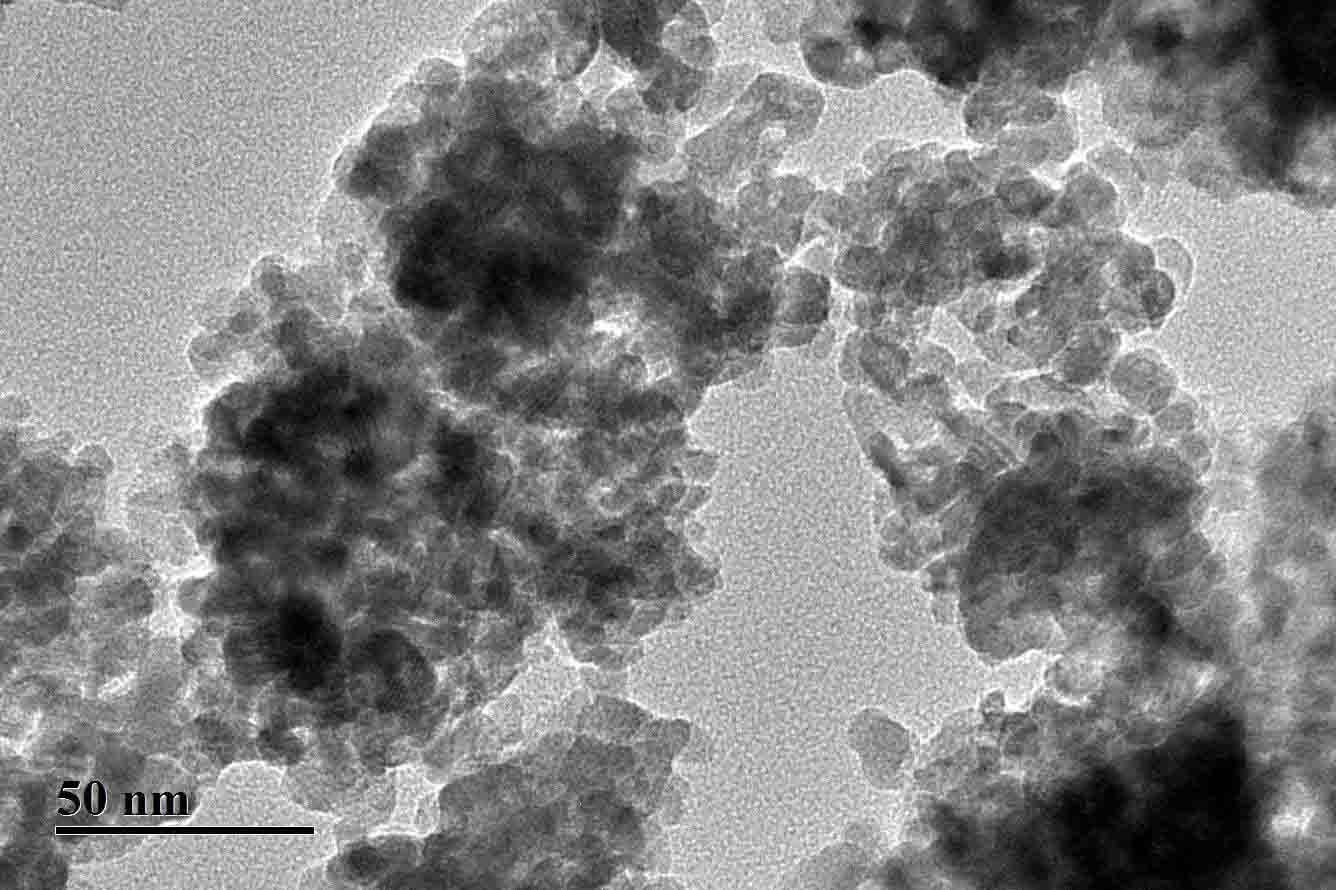

Supplement: Supplementary file 3 — Source Data [file 41467_2023_38336_MOESM3_ESM.zip › Source_Data_for_Figures_in_Supplementary_Information/Source_Data_Supplementary_Figure_39/Supplementary_Figure_39e.jpg]

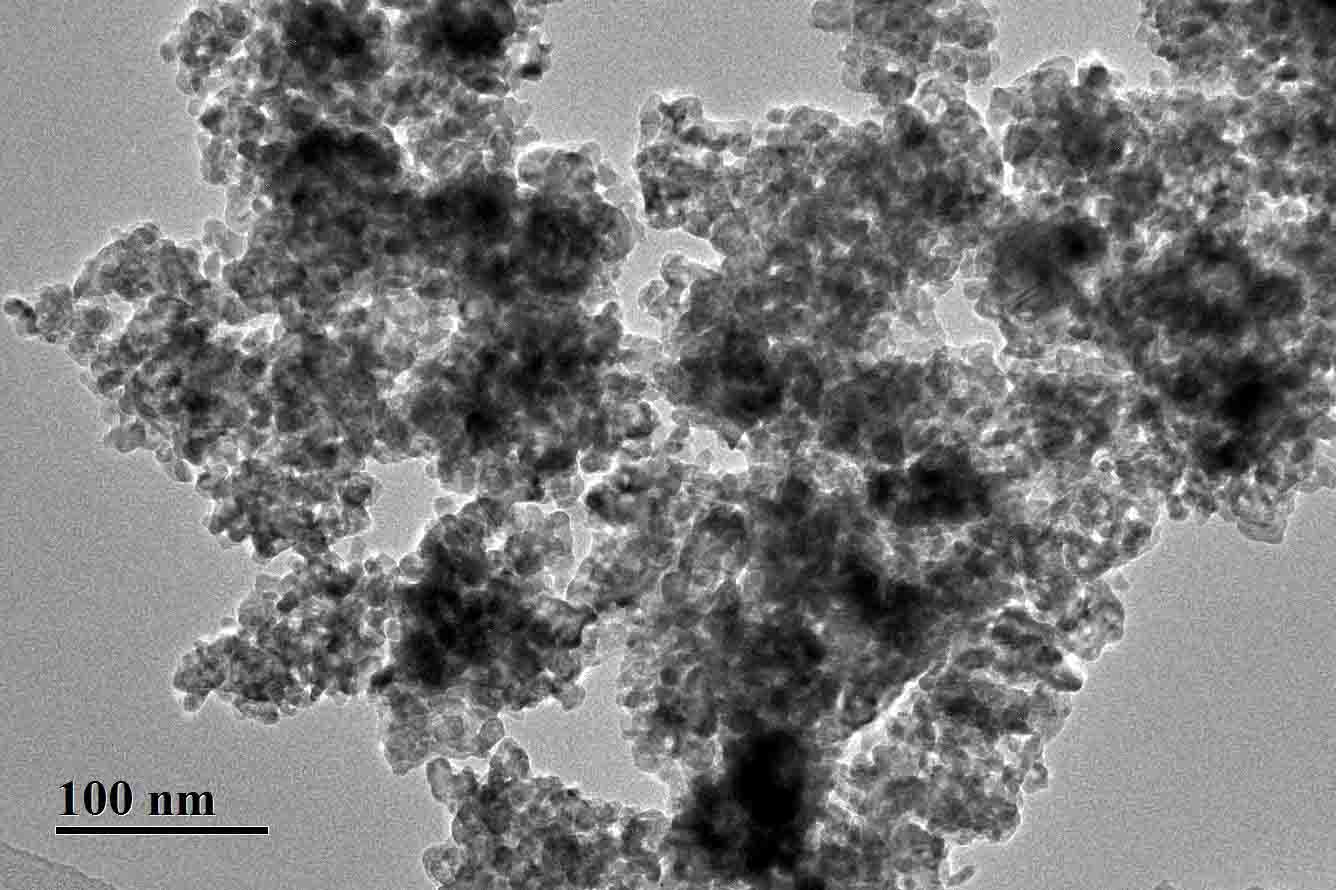

Supplement: Supplementary file 3 — Source Data [file 41467_2023_38336_MOESM3_ESM.zip › Source_Data_for_Figures_in_Supplementary_Information/Source_Data_Supplementary_Figure_39/Supplementary_Figure_39g.jpg]

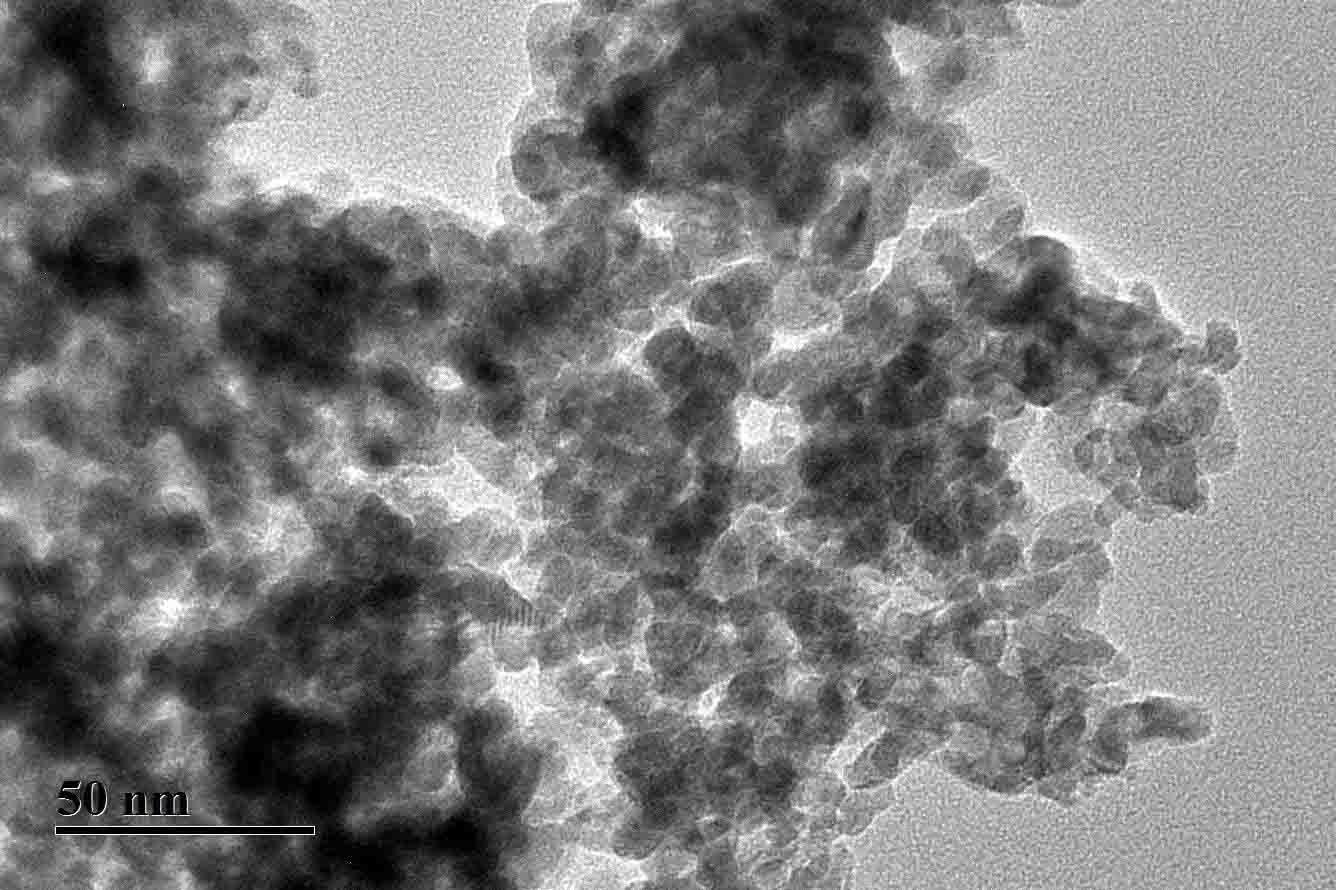

Supplement: Supplementary file 3 — Source Data [file 41467_2023_38336_MOESM3_ESM.zip › Source_Data_for_Figures_in_Supplementary_Information/Source_Data_Supplementary_Figure_39/Supplementary_Figure_39h.jpg]

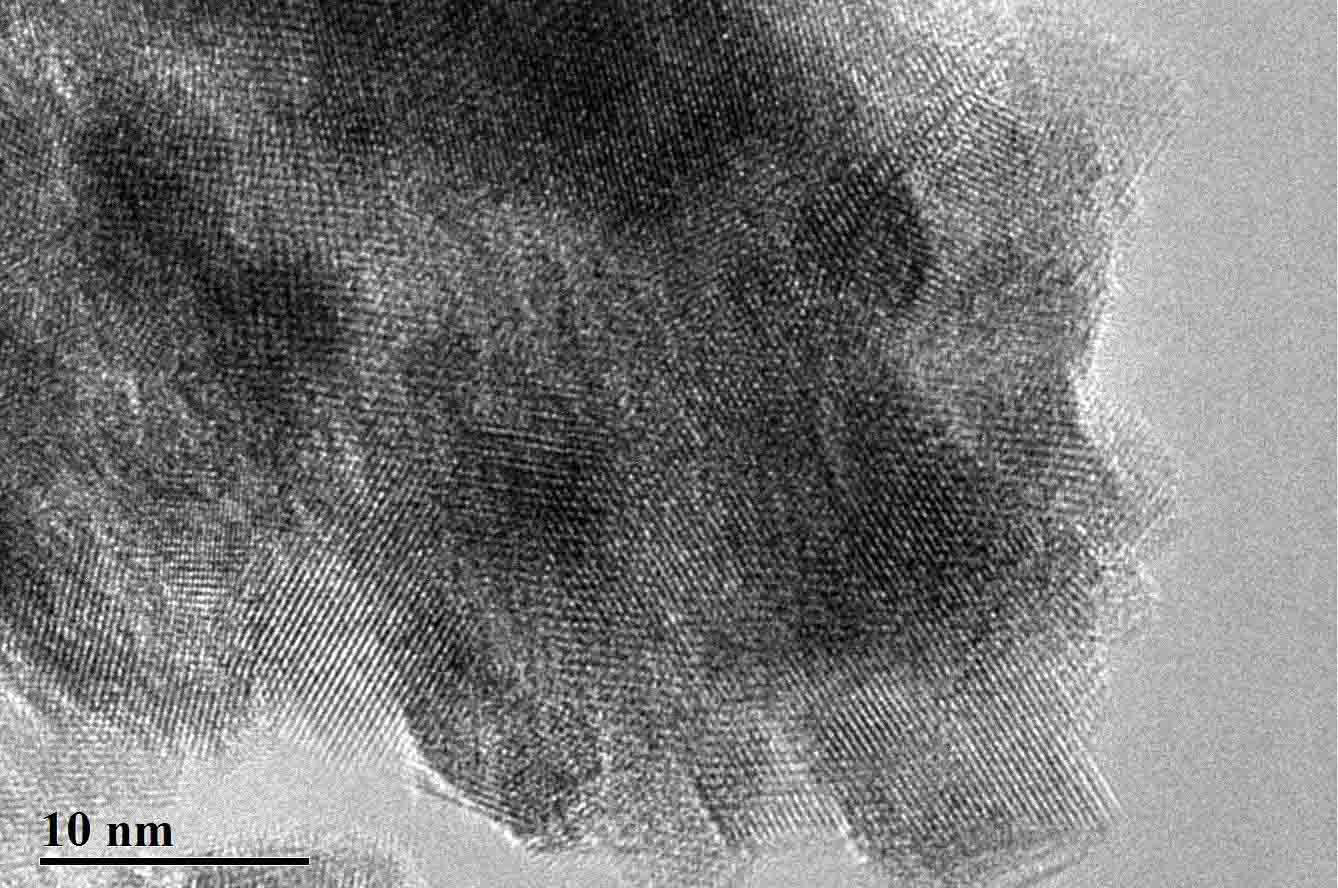

Supplement: Supplementary file 3 — Source Data [file 41467_2023_38336_MOESM3_ESM.zip › Source_Data_for_Figures_in_Supplementary_Information/Source_Data_Supplementary_Figure_39/Supplementary_Figure_39j.jpg]

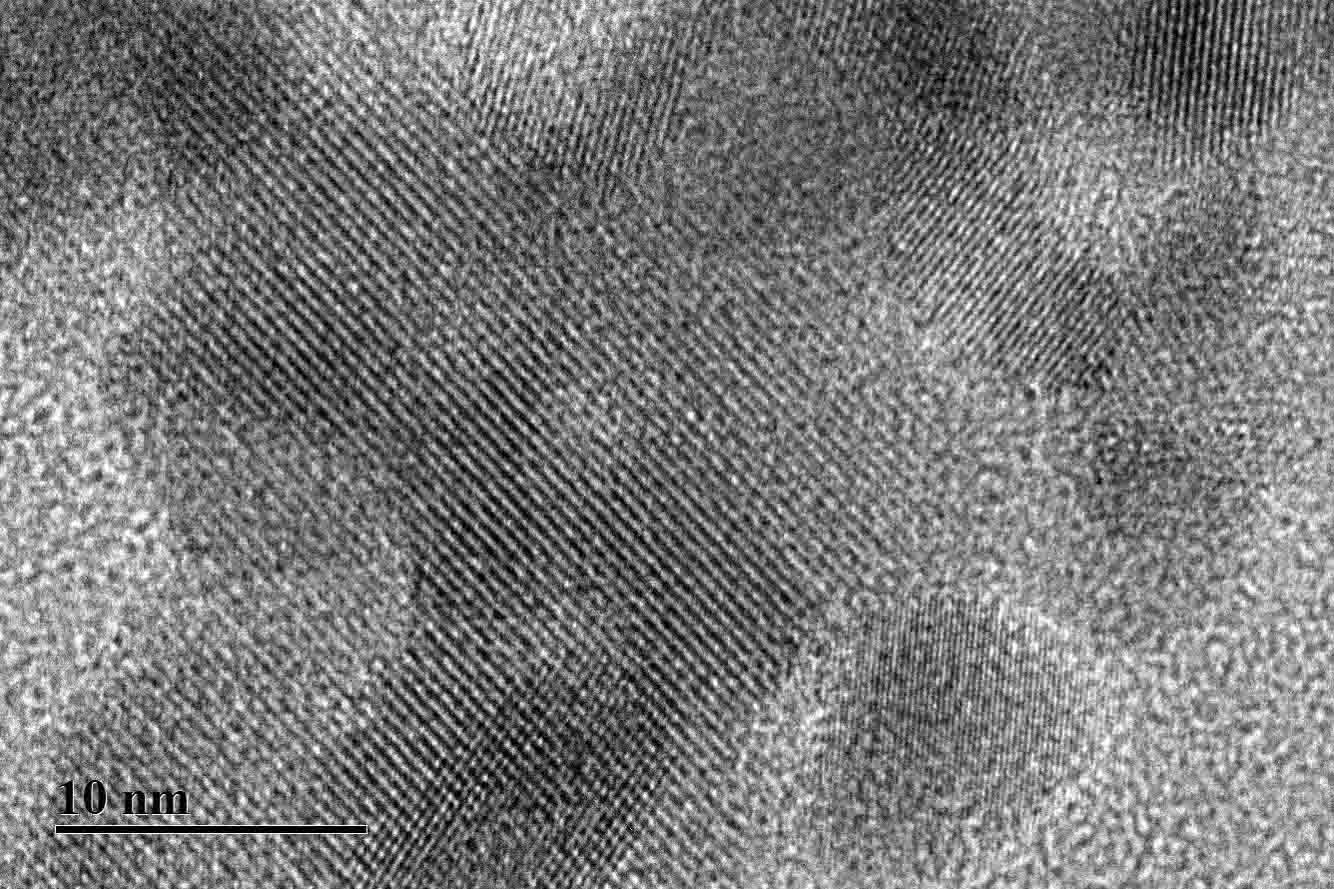

Supplement: Supplementary file 3 — Source Data [file 41467_2023_38336_MOESM3_ESM.zip › Source_Data_for_Figures_in_Supplementary_Information/Source_Data_Supplementary_Figure_39/Supplementary_Figure_39k.jpg]

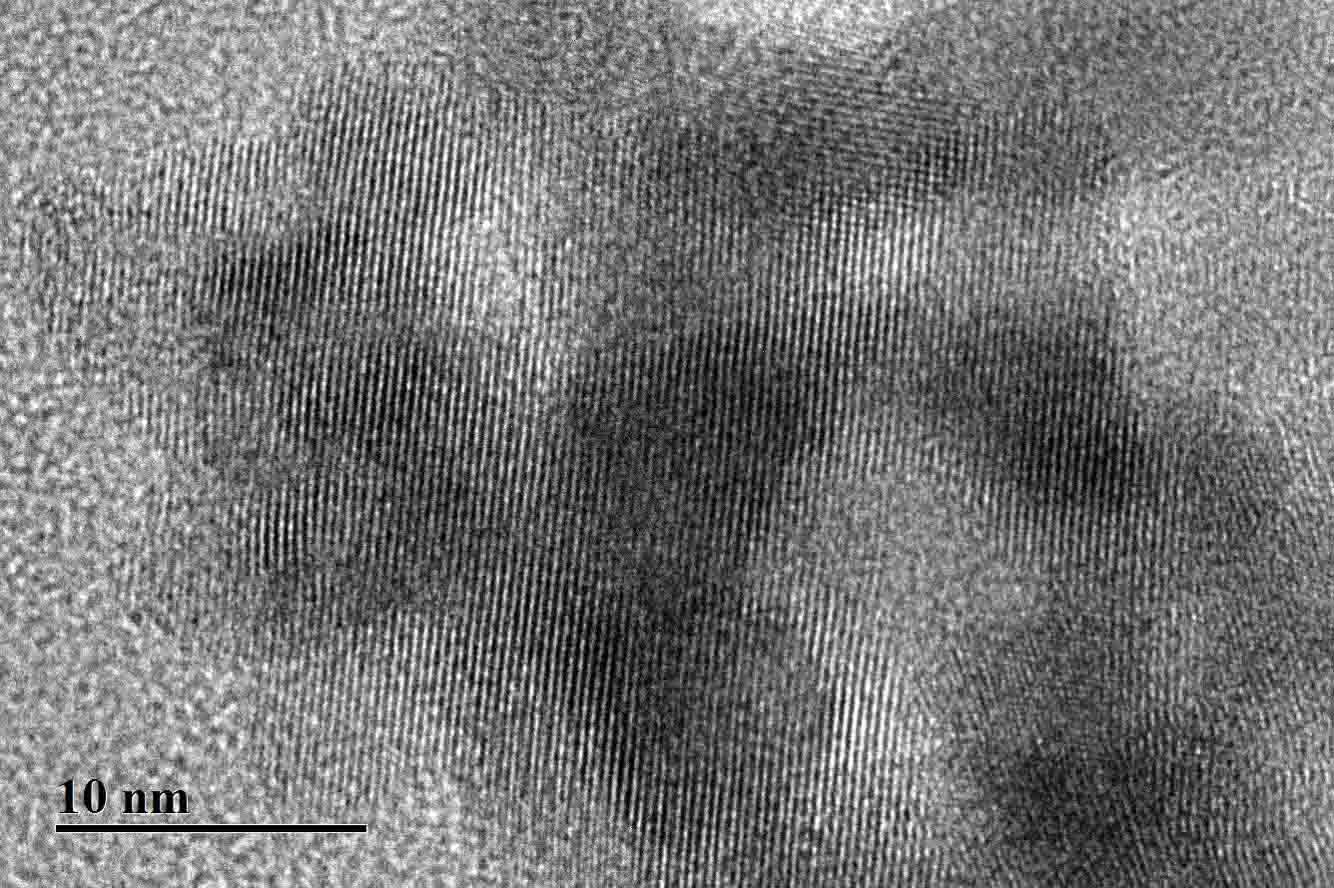

Supplement: Supplementary file 3 — Source Data [file 41467_2023_38336_MOESM3_ESM.zip › Source_Data_for_Figures_in_Supplementary_Information/Source_Data_Supplementary_Figure_39/Supplementary_Figure_39l.jpg]

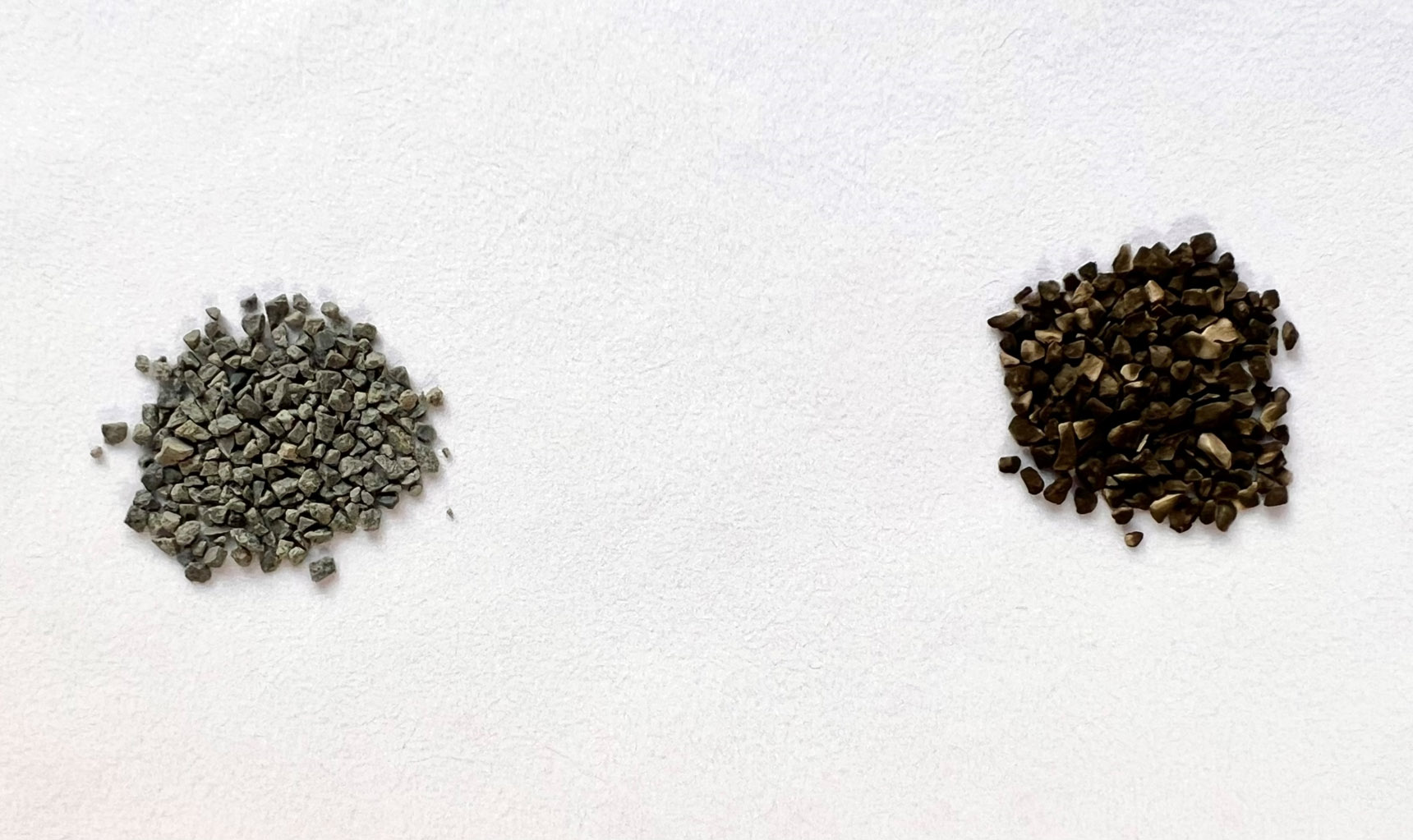

Supplement: Supplementary file 3 — Source Data [file 41467_2023_38336_MOESM3_ESM.zip › Source_Data_for_Figures_in_Supplementary_Information/Source_Data_Supplementary_Figure_41.jpg]

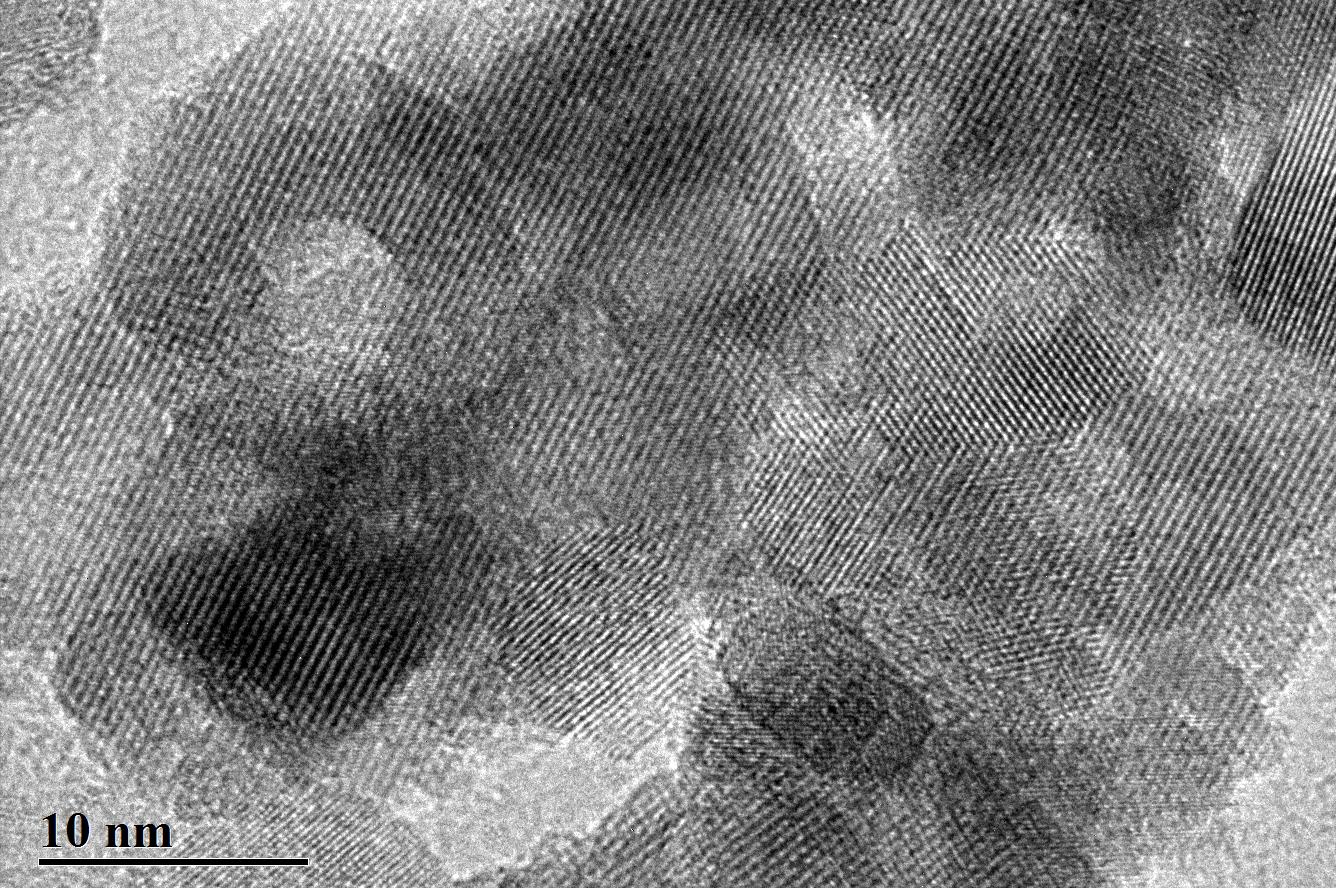

Supplement: Supplementary file 3 — Source Data [file 41467_2023_38336_MOESM3_ESM.zip › Source_Data_for_Figures_in_Main_Article/Source_data_Fig_1/Fig_1a.jpg]

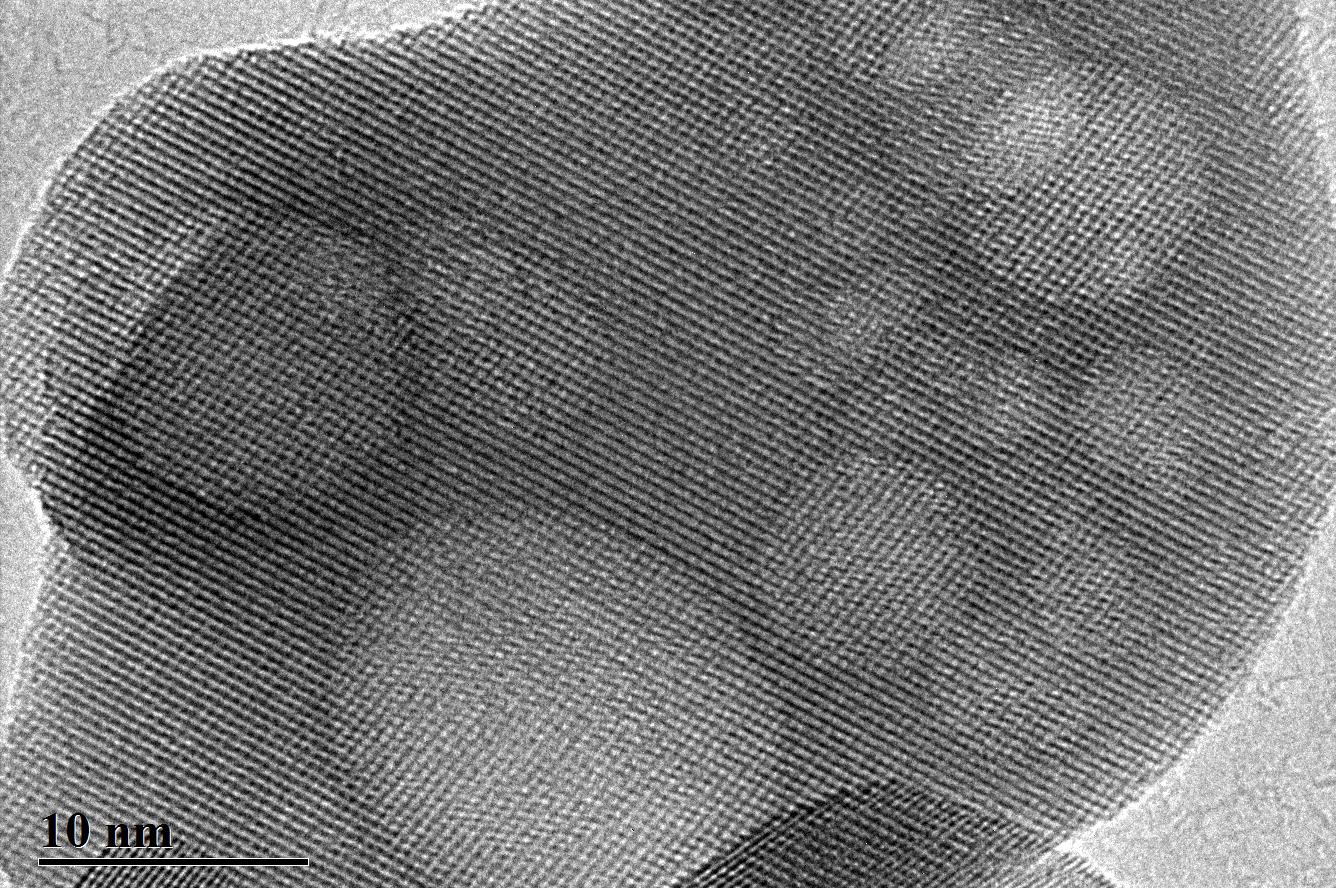

Supplement: Supplementary file 3 — Source Data [file 41467_2023_38336_MOESM3_ESM.zip › Source_Data_for_Figures_in_Main_Article/Source_data_Fig_1/Fig_1b.jpg]

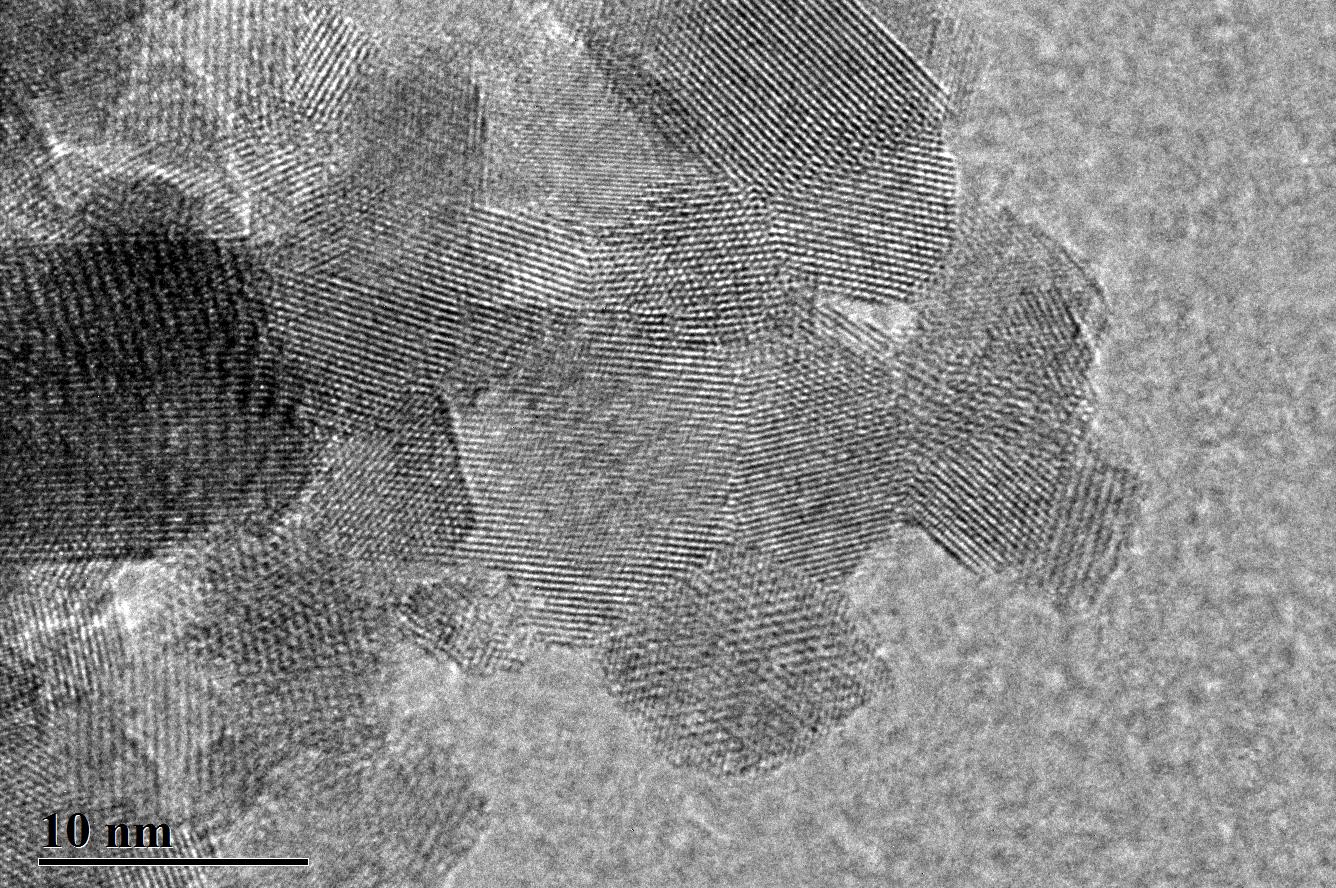

Supplement: Supplementary file 3 — Source Data [file 41467_2023_38336_MOESM3_ESM.zip › Source_Data_for_Figures_in_Main_Article/Source_data_Fig_1/Fig_1c.jpg]

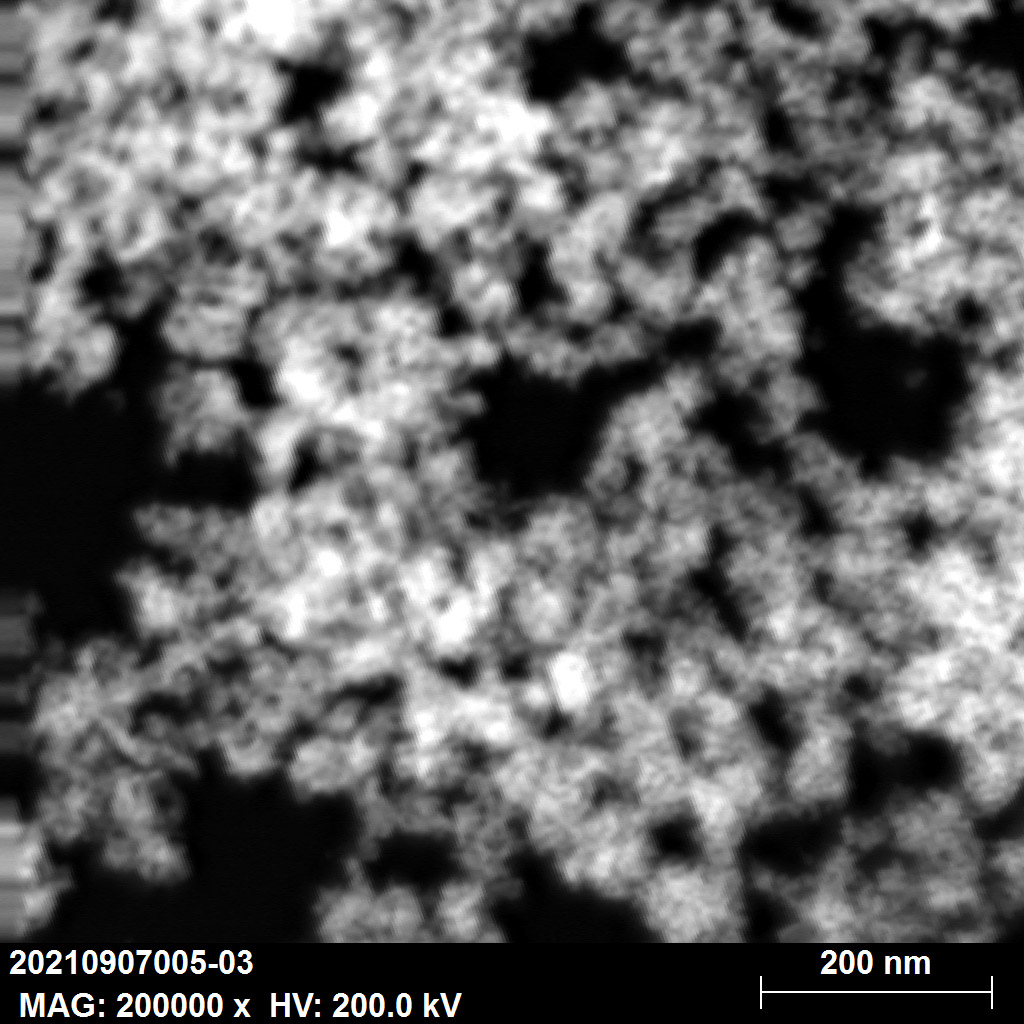

Supplement: Supplementary file 3 — Source Data [file 41467_2023_38336_MOESM3_ESM.zip › Source_Data_for_Figures_in_Main_Article/Source_data_Fig_1/Fig_1e.jpg]

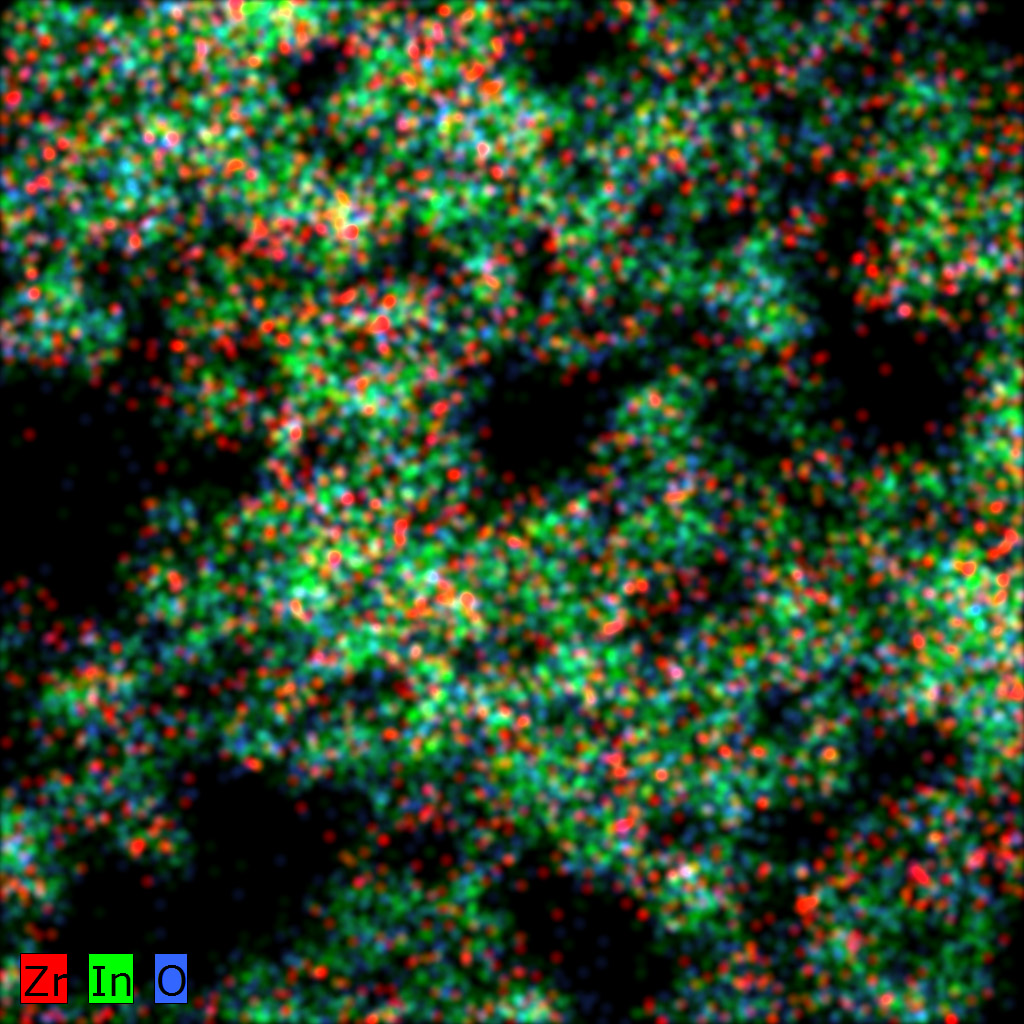

Supplement: Supplementary file 3 — Source Data [file 41467_2023_38336_MOESM3_ESM.zip › Source_Data_for_Figures_in_Main_Article/Source_data_Fig_1/Fig_1f.jpg]

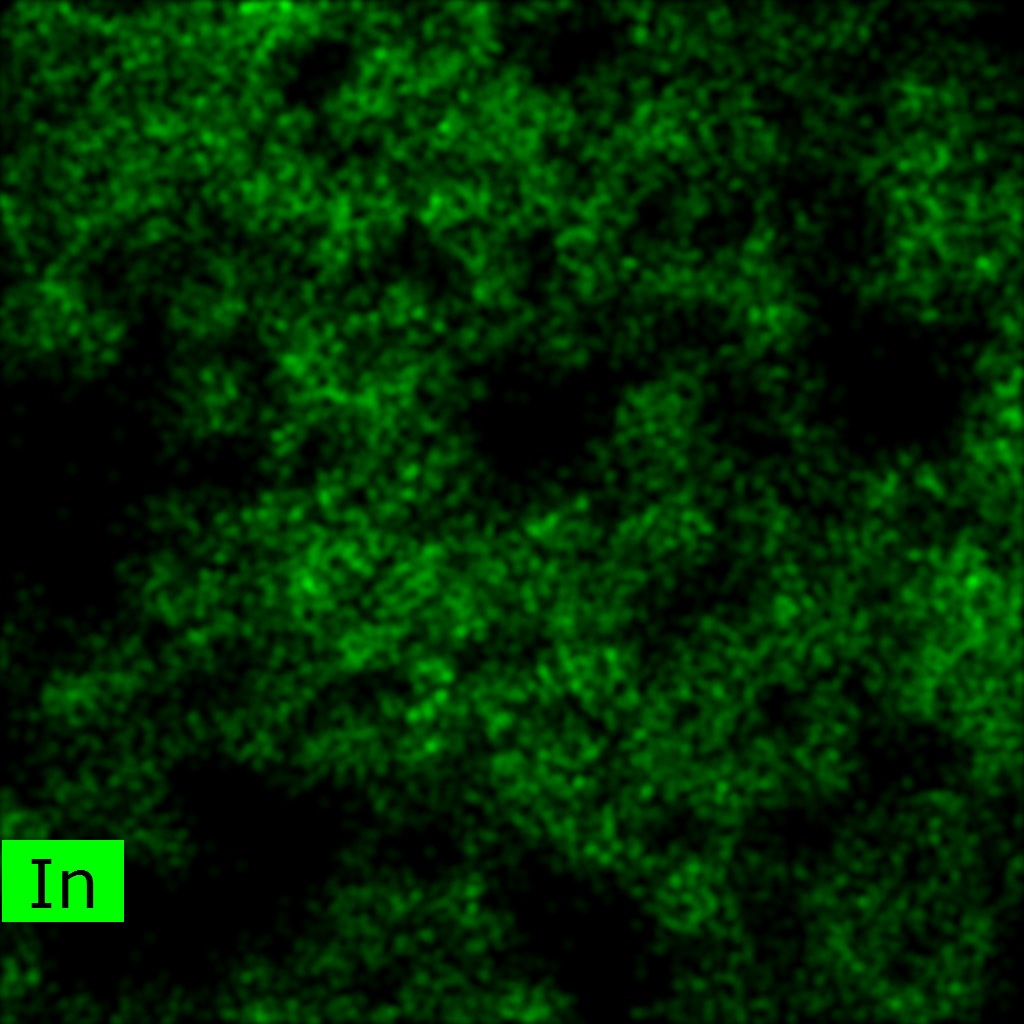

Supplement: Supplementary file 3 — Source Data [file 41467_2023_38336_MOESM3_ESM.zip › Source_Data_for_Figures_in_Main_Article/Source_data_Fig_1/Fig_1g.jpg]

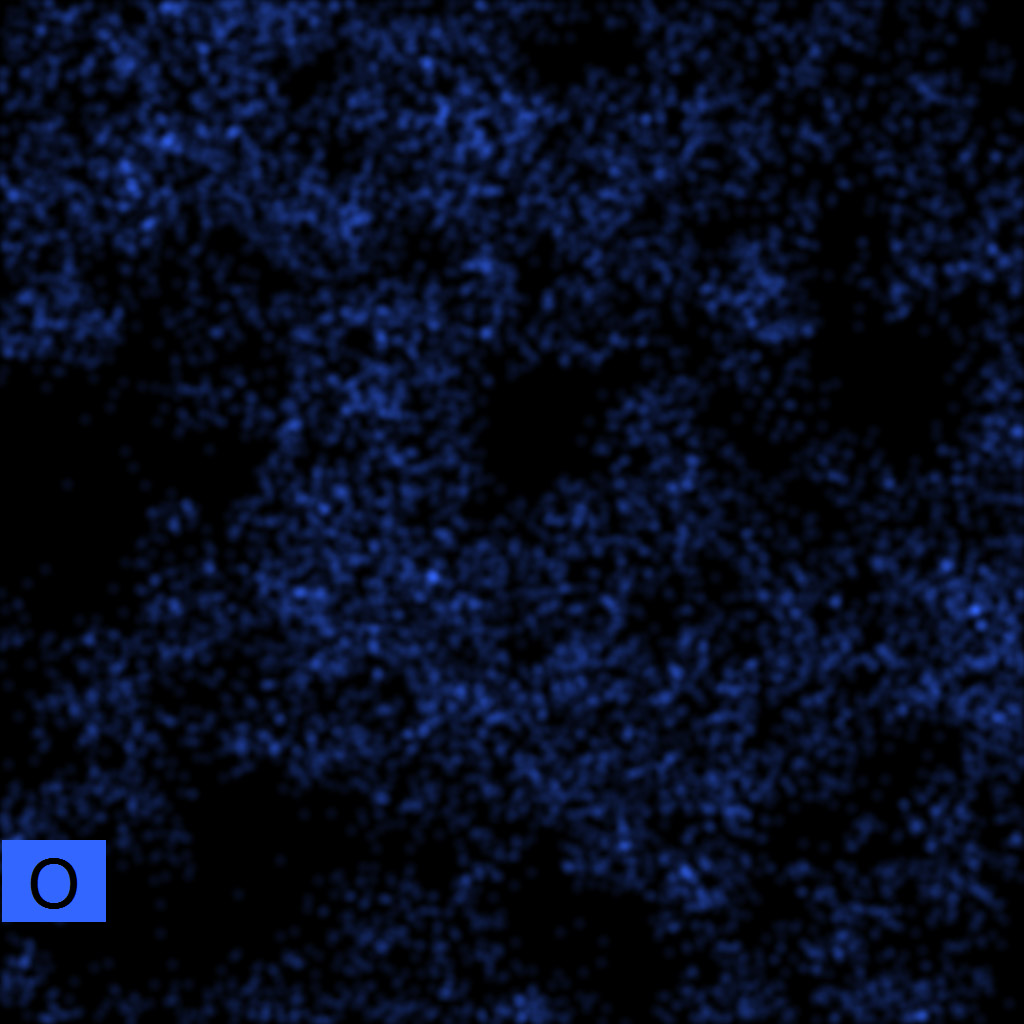

Supplement: Supplementary file 3 — Source Data [file 41467_2023_38336_MOESM3_ESM.zip › Source_Data_for_Figures_in_Main_Article/Source_data_Fig_1/Fig_1h.jpg]

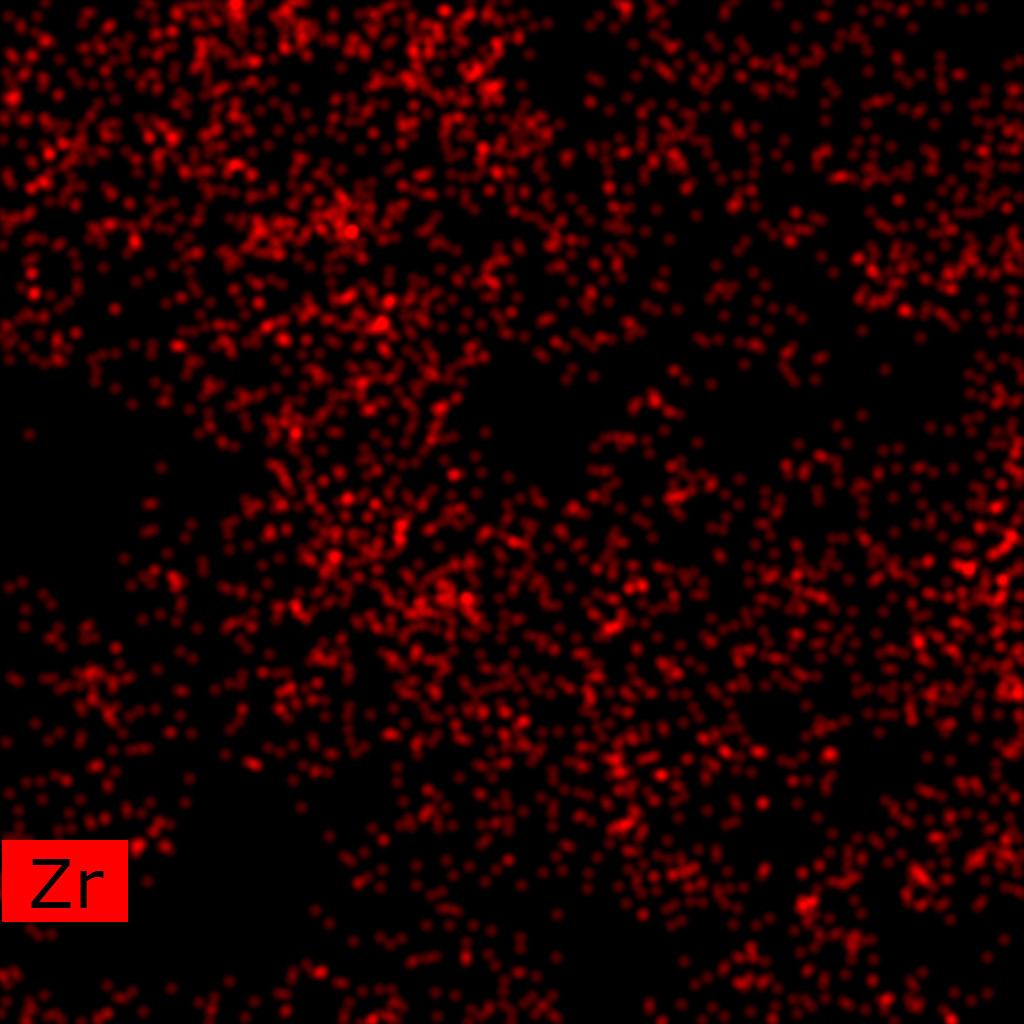

Supplement: Supplementary file 3 — Source Data [file 41467_2023_38336_MOESM3_ESM.zip › Source_Data_for_Figures_in_Main_Article/Source_data_Fig_1/Fig_1i.jpg]

## Slide 1
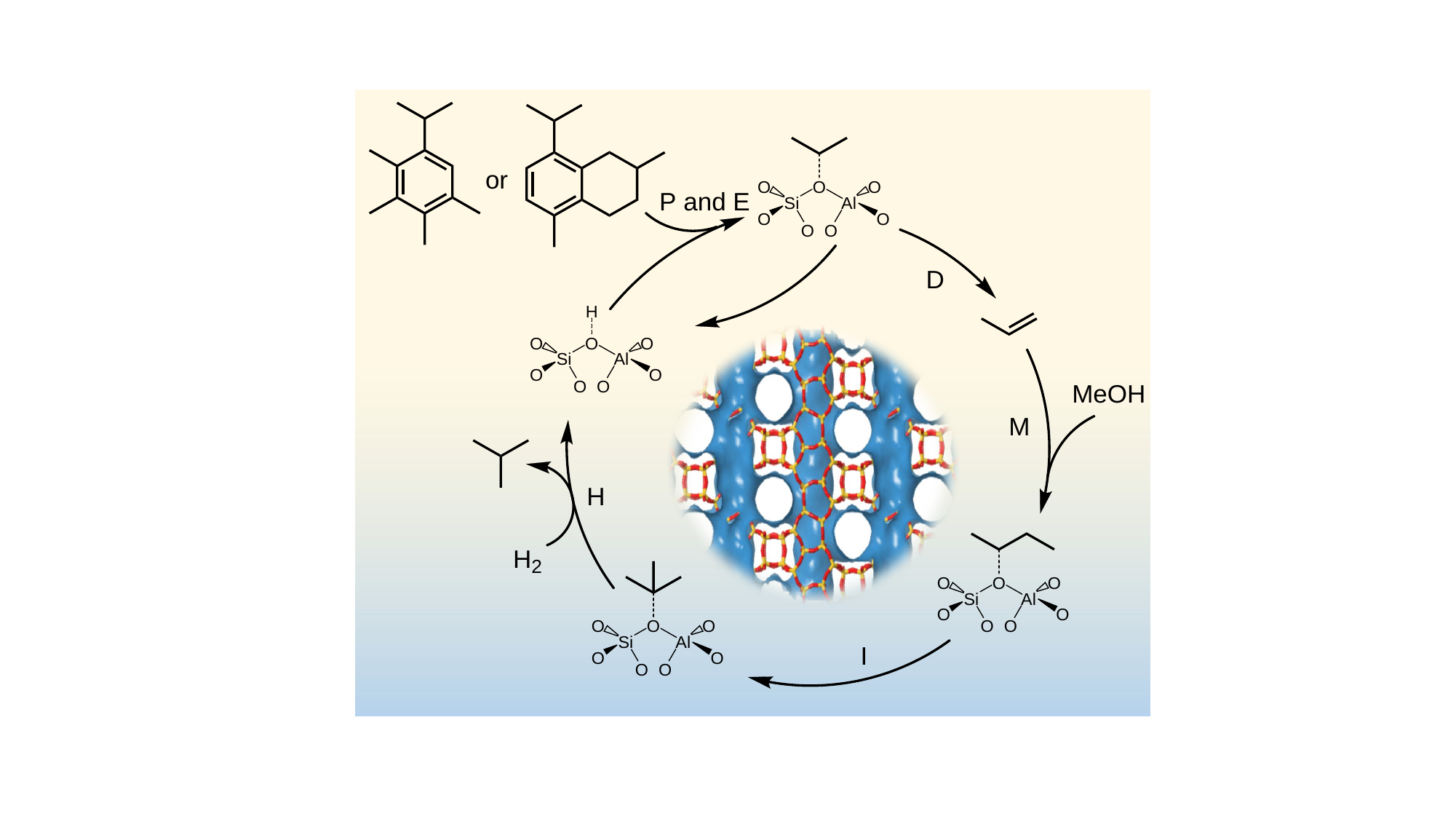

Supplement: Supplementary file 3 — Source Data [file 41467_2023_38336_MOESM3_ESM.zip › Source_Data_for_Figures_in_Main_Article/Source_data_Fig_7/Source_data_Fig_7a.pptx]

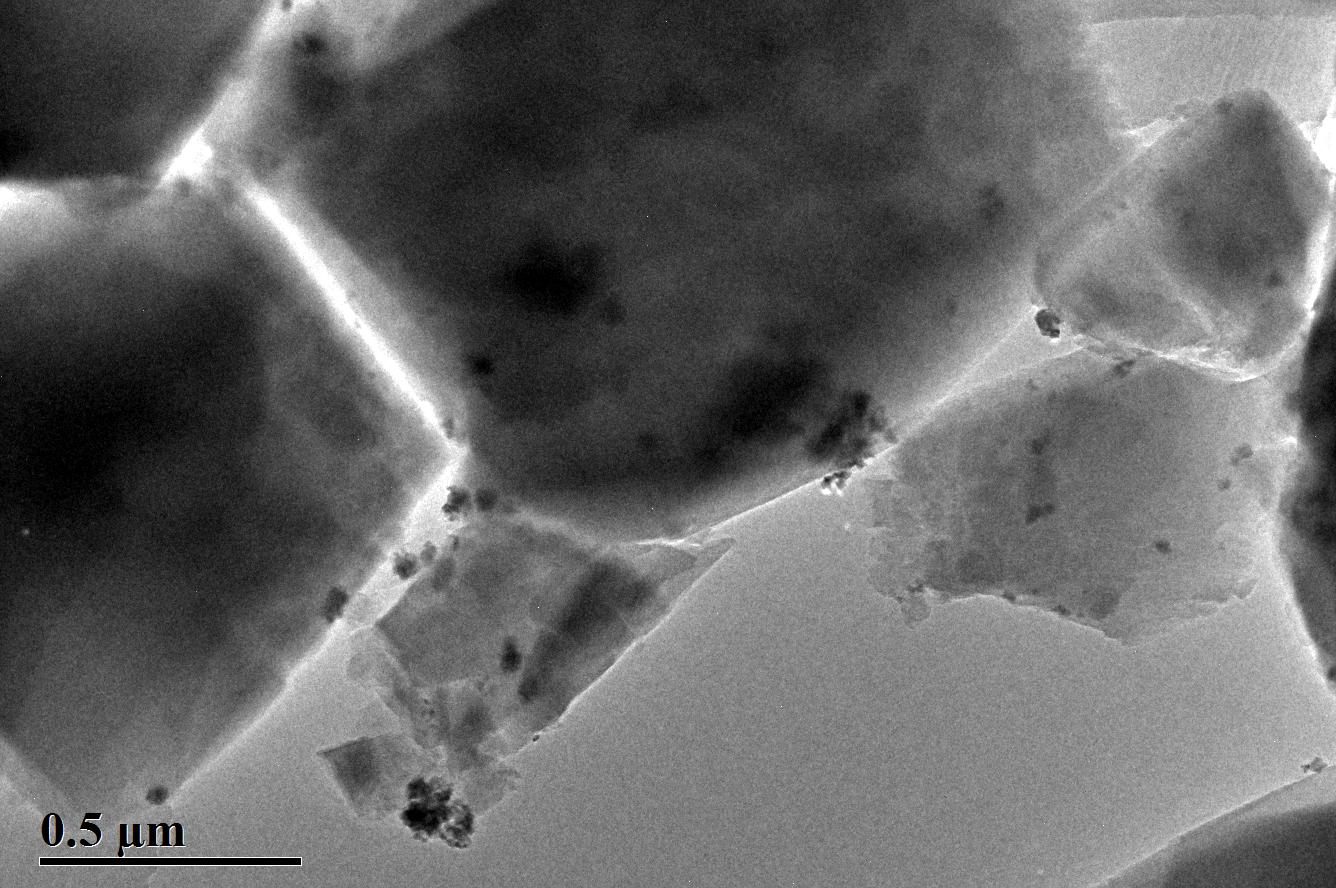

Supplement: Supplementary file 3 — Source Data [file 41467_2023_38336_MOESM3_ESM.zip › Source_Data_for_Figures_in_Main_Article/Source_data_Fig_9/Fig_9g1.jpg]

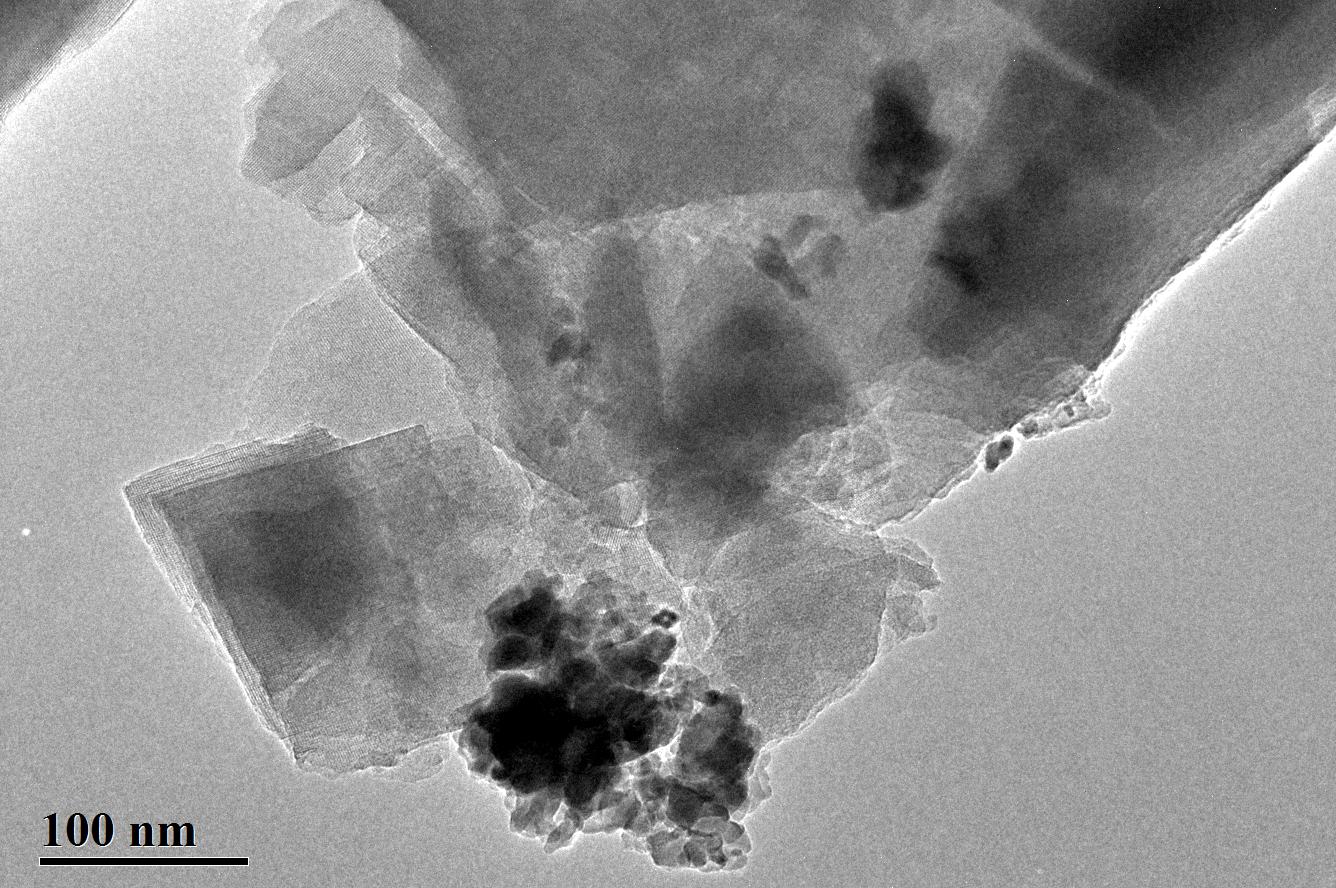

Supplement: Supplementary file 3 — Source Data [file 41467_2023_38336_MOESM3_ESM.zip › Source_Data_for_Figures_in_Main_Article/Source_data_Fig_9/Fig_9g2.jpg]

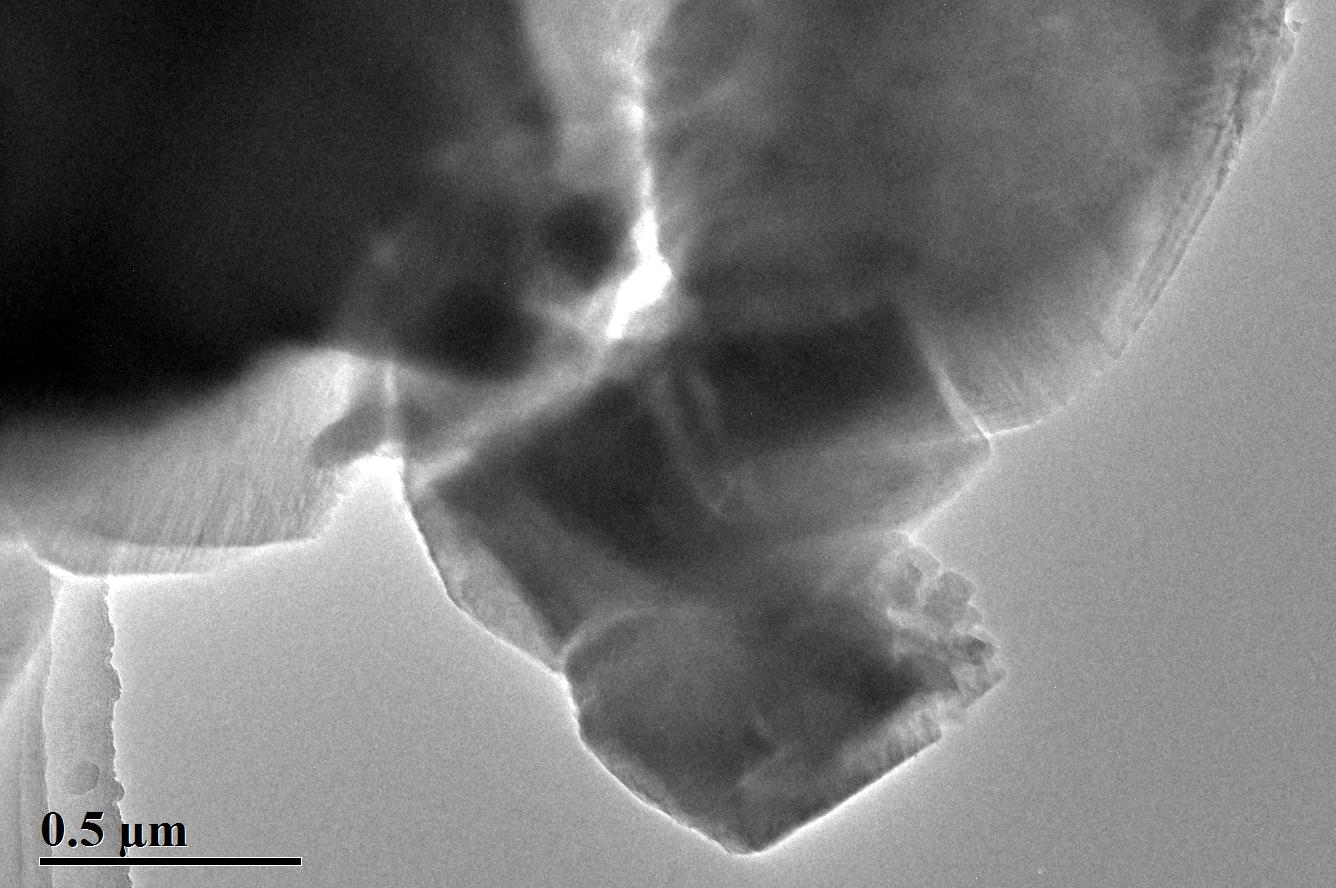

Supplement: Supplementary file 3 — Source Data [file 41467_2023_38336_MOESM3_ESM.zip › Source_Data_for_Figures_in_Main_Article/Source_data_Fig_9/Fig_9h1.jpg]

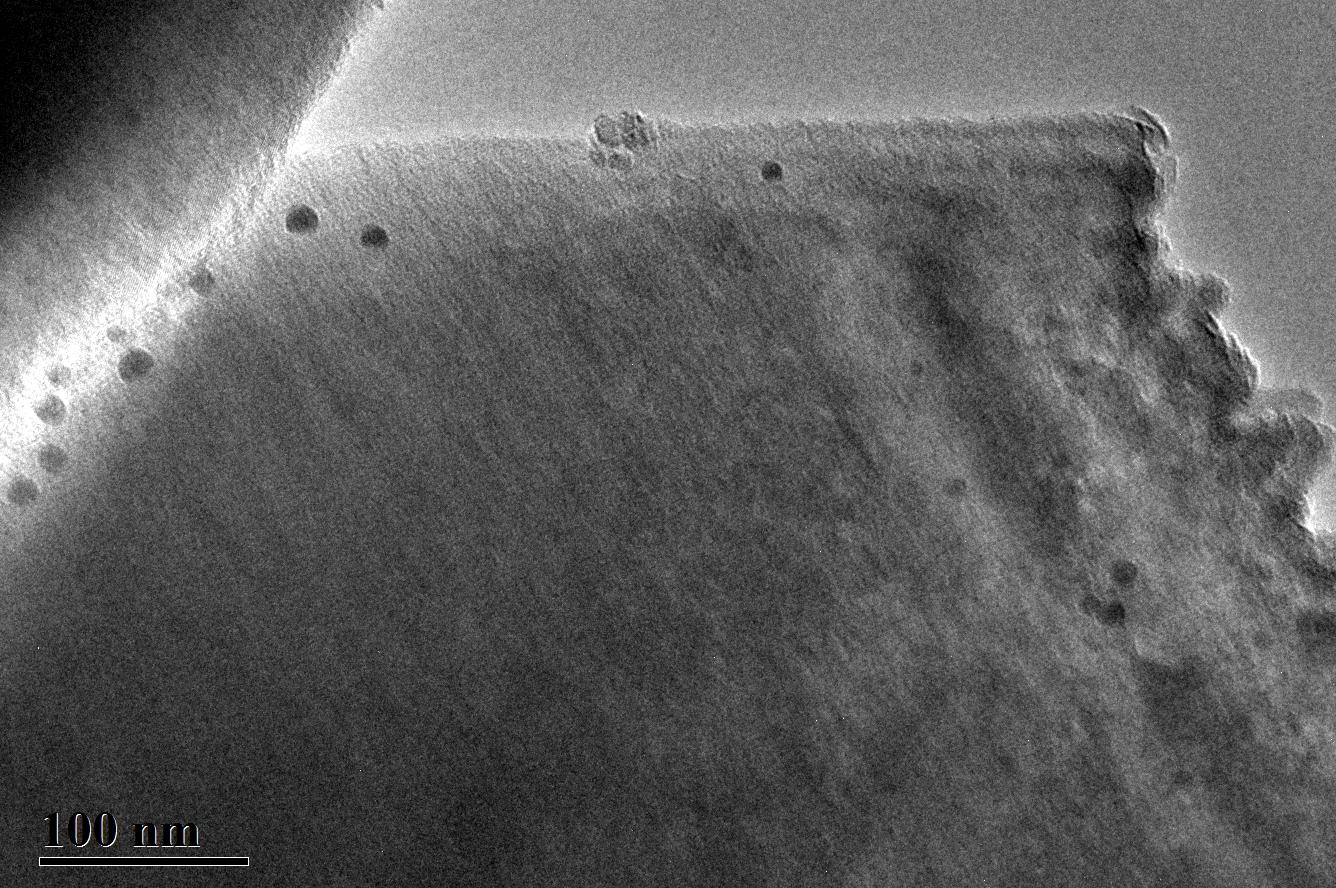

Supplement: Supplementary file 3 — Source Data [file 41467_2023_38336_MOESM3_ESM.zip › Source_Data_for_Figures_in_Main_Article/Source_data_Fig_9/Fig_9h2.jpg]

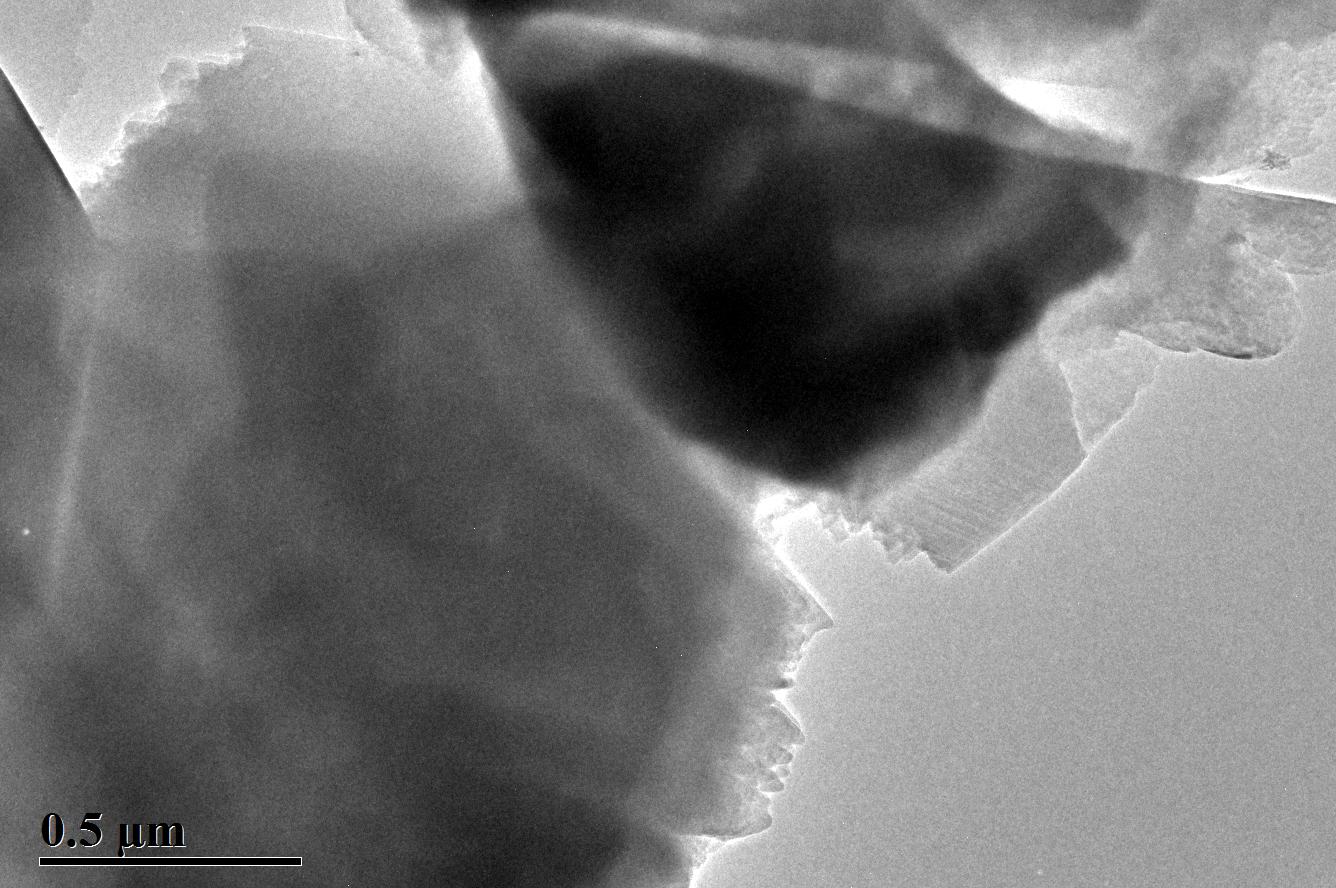

Supplement: Supplementary file 3 — Source Data [file 41467_2023_38336_MOESM3_ESM.zip › Source_Data_for_Figures_in_Main_Article/Source_data_Fig_9/Fig_9i1.jpg]

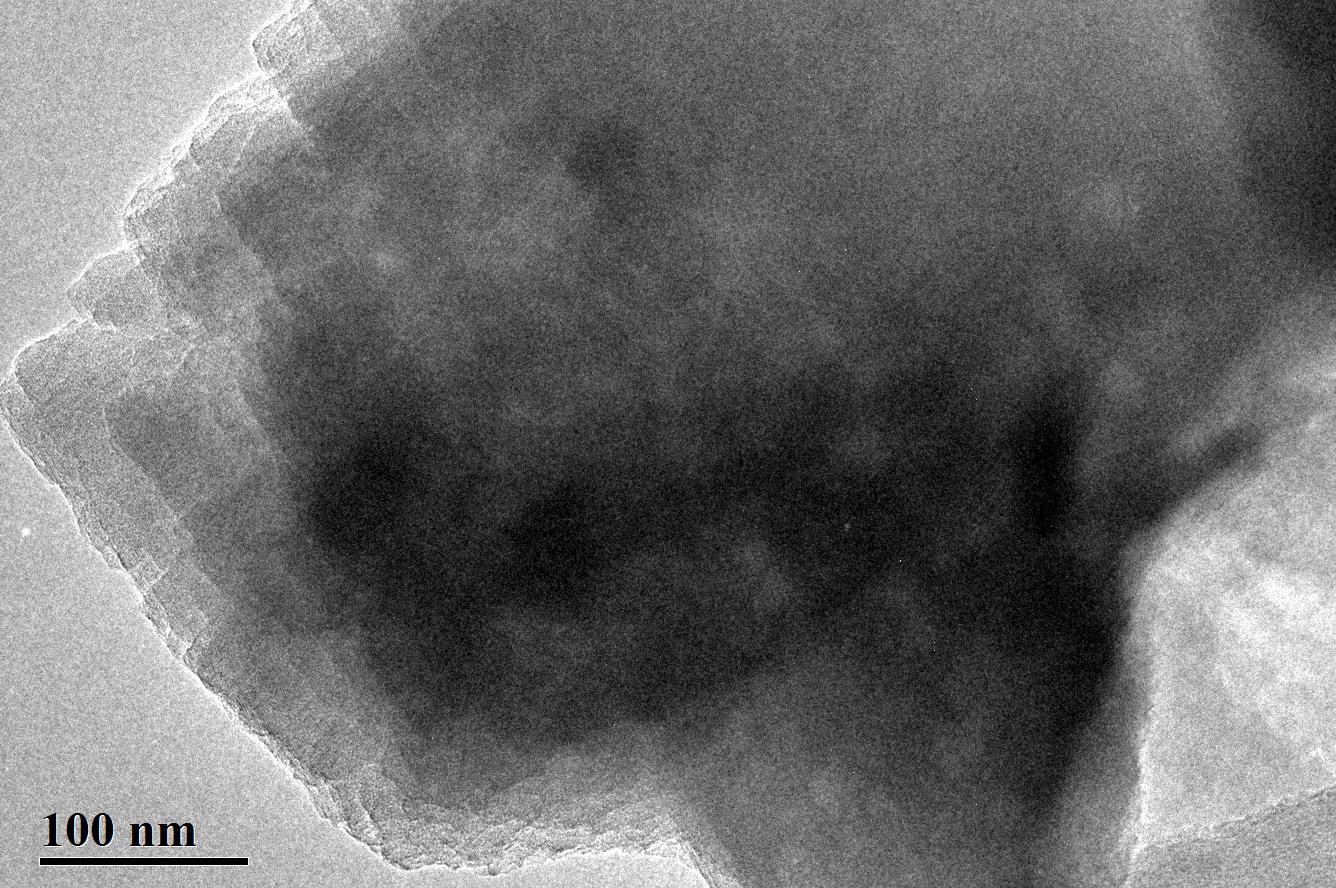

Supplement: Supplementary file 3 — Source Data [file 41467_2023_38336_MOESM3_ESM.zip › Source_Data_for_Figures_in_Main_Article/Source_data_Fig_9/Fig_9i2.jpg]
